# Supplementary material for: Catalytic hydrogenation of olefins by a multifunctional molybdenum-sulfur complex
Source: Nat Commun. 2024 Jan 27;15:797. doi: 10.1038/s41467-024-45018-3 (PMC10821942; doi:10.1038/s41467-024-45018-3)
Supplement: Supplementary file 1 — Supplementary Information [file 41467_2024_45018_MOESM1_ESM.docx]

Supplementary Information

**Catalytic Hydrogenation of Olefins by a Multifunctional Molybdenum-Sulfur Complex**

Minghui Xue^1^, Zhiqiang Peng^2^, Keyan Tao^2^, Jiong Jia^1^, Datong Song^3^*, Chen-Ho Tung^1^ & Wenguang Wang ^1,2^*

^1^School of Chemistry and Chemical Engineering, Shandong University, Jinan, 250100, China

^2^College of Chemistry, Beijing Normal University, Beijing 100875, China

^3^Davenport Chemical Research Laboratories, Department of Chemistry, University of Toronto, Toronto, Ontario M5S 3H6, Canada.

*Email: d.song@utoronto.ca; [wwg@bnu.edu.cn](mailto:wwg@bnu.edu.cn)

**Table of Contents**

[General Information 3](#_Toc155010751)

[Supplementary Methods 4](#_Toc155010752)

[Synthesis and Characterization 4](#_Toc155010753)

[Optimization Studies 7](#_Toc155010754)

[Catalytic Hydrogenation of Alkenes 8](#_Toc155010755)

[Hydrogenation of a mixture of 2-octene and 4-octene 13](#_Toc155010756)

[Scale-Up Experiment 14](#_Toc155010757)

[Catalytic Hydrogenation of 5a by Complex 4 15](#_Toc155010758)

[Deuterium Labeling Experiment 16](#_Toc155010759)

[Determination of the Kinetic Isotope Effect 17](#_Toc155010760)

[The Hammett-Analysis 22](#_Toc155010761)

[Supplementary Data 24](#_Toc155010762)

[HRMS Spectra 24](#_Toc155010763)

[IR Spectrum 26](#_Toc155010764)

[NMR Spectra 27](#_Toc155010765)

[Crystal Data and Structure Refinement Parameters 50](#_Toc155010766)

[DFT Calculations 54](#_Toc155010767)

[Supplementary References 56](#_Toc155010768)

## General Information

All procedures were performed in flame-dried glassware using standard Schlenk techniques or in a glovebox under a nitrogen atmosphere. Tetrahydrofuran and n-hexane were dried and degassed by Solvent Purification Systems (Innovation Technology). Fluorobenzene was dried over P_2_O_5_ for two days under nitrogen and degassed by the freeze-pump-thaw method. CDCl_3_, *d*_8_-THF and C_6_D_5_Cl were purchased from Cambridge Isotope Laboratories and dried with activated 4 Å molecular sieves. All reagents were purchased from Sigma-Aldrich and used without further purification unless otherwise noted. Complex Cp*Mo(1,2-Ph_2_PC_6_H_4_S)(*η*^2^-NCMe) was prepared according to the reported procedures.^1^

NMR spectra were recorded on Bruker 500 MHz spectrometer. The chemical shifts are reported in ppm relative to either the residual solvent peak or 1,3,5-trimethoxybenzene as an internal standard. IR spectra were recorded on PerkinElmer Spectrum Two FT-IR. The HRMS (ESI) was performed on a Q-TOF mass spectrometer, Bruker impact II Mass Spectrometer Q-TOF6510. Elemental analyses (C, N, and H) were performed on Elementar Vario Micro Cube analyzer, and samples were handled under N_2_ atmosphere wherever appropriate. Single crystals with appropriate dimensions were selected under an optical microscope and quickly coated with high vacuum grease (Dow Corning Corporation) to prevent decomposition. Crystallographic data were collected using a Bruker D8 VENTURE with Mo Kα radiation (λ = 0.71073 Å) and micro-focus Cu Kα radiation (λ = 1.5418 Å) at 173 K. Crystal data collection and refinement parameters were summarized in Supplementary Tables 3-6.

## Supplementary Methods

### Synthesis and Characterization

**Cp*Mo(1,2-Ph_2_PC_6_H_4_S)(*η*^2^-C_2_H_2_)**, **1**-C_2_H_2_**.** In a 100 mL Schlenk flask charged with a magnetic stir bar, Cp*Mo(1,2-Ph_2_PC_6_H_4_S)(*η*^2^-NCMe) (150 mg, 0.265 mmol) was dissolved in THF (15 mL). And then, the flask was taken out from the glovebox and immersed in a liquid nitrogen bath. The brown solution was frozen and gently degassed through freeze-pump-thaw cycle and then pressurized with 1 atm of C_2_H_2_. After stirring at room temperature for 10 min, a green solution was obtained. The solution was filtered through Celite in a N_2_ glovebox and dried under vacuum to give **1**-C_2_H_2_ (127 mg, 87% yield). Single crystals suitable for X-ray diffraction were obtained by evaporating a concentrated hexane solution of **1-**C_2_H_2_ at room temperature under N_2_ atmosphere. **^1^H NMR** (500 MHz, *d*_8_-THF, 273 K, ppm) 10.49 (s, 1H), 9.48 (d, *J* = 19.1 Hz, 1H), 7.59 (s, 3H), 7.51 (d, *J* = 6.8 Hz, 1H), 7.36 (s, 5H), 7.29 (s, 3H), 7.08 (t, *J* = 6.1 Hz, 1H), 6.93 (d, *J* = 6.8 Hz, 1H), 1.51 (s, 15H). **^13^C{^1^H} NMR** (126 MHz, *d*_8_-THF, 273 K, ppm) 190.3, 185.0, 184.8, 161.9, 161.6, 137.9, 137.5, 137.2, 137.0, 133.7, 133.4, 133.3, 133.2, 132.9, 132.7, 132.7, 129.8, 129.7, 129.3, 128.7, 128.6, 127.8, 127.7, 127.5, 127.4, 122.4, 122.4, 115.2, 103.4, 10.3. **^31^P{^1^H} NMR** (202 MHz, *d*_8_-THF, 273 K, ppm) 97.5. **HRMS (m/z)**: [**1**-C_2_H_2_]^+^ calcd for C_30_H_31_MoPS, 552.0938; found, 552.0937. **Analysis** (calcd., found for C_30_H_31_MoPS): C (65.45, 65.15), H (5.68, 5.88).

**[Cp*Mo(1,2-Ph_2_PC_6_H_4_S−CH=CH_2_)(Py)][BAr^F^_4_]**, **2.** Under N_2_ atmosphere, a green solution of **1-**C_2_H_2_ (30 mg, 0.054 mmol**)** in 3 mL of THF was cooled to -20 ℃. The resulting solution was treated with [C_5_H_5_NH][BAr^F^_4_] (51 mg, 0.053 mmol) in 2 mL of THF, which caused a change of the solution color from green to brown. After the removal of the solvent under vacuum, the product was washed with hexane (5 mL) and then recrystallized from PhF/hexane at -30 ℃. Yield: 61 mg, 75%. **^1^H NMR** (500 MHz, *d*_8_-THF, ppm) 8.37 (d, *J* = 5.3 Hz, 2H), 8.15 (d, *J* = 6.6 Hz, 1H), 7.80 (s, 8H), 7.72 (t, *J* = 7.5 Hz, 1H), 7.64 (t, *J* = 6.4 Hz, 1H), 7.58 (s, 5H), 7.53 (t, *J* = 6.2 Hz, 4H), 7.25 (t, *J* = 7.9 Hz, 3H), 7.17 (m, 4H), 7.08 (t, *J* = 6.8 Hz, 2H), 5.37 (m, 1H), 1.68 (t, *J* = 4.5 Hz, 1H), 1.31 (s, 15H), 1.09 (d, *J* = 3.7 Hz, 1H). **^13^C{^1^H} NMR** (126 MHz, *d*_8_-THF, ppm) 162.6 (q, *J* = 49.8 Hz), 157.7, 140.3, 139.8, 139.6, 135.4, 135.2, 134.9, 134.0, 133.9, 133.8, 133.7, 133.3, 133.3, 133.2, 133.1, 132.9, 132.9, 132.8, 132.1, 132.1, 131.3, 131.1, 130.22 (q, *J* = 2.8 Hz), 129.97 (q, *J* = 2.8 Hz). 129.8, 129.7, 129.7, 129.7, 129.6, 129.5 (q, *J* = 2.8 Hz), 129.3, 129.2, 128.6, 126.4, 126.4, 124.2, 122.1, 118.0, 104.1, 83.9, 83.8, 54.7, 32.5, 10.2. **^31^P{^1^H} NMR** (202 MHz, *d*_8_-THF, ppm) 80.1. **HRMS (m/z)**: [**2**]^+^ calcd. for C_35_H_37_MoNPS, 632.1438; found, 632.1438. **Analysis** (calcd., found for C_67_H_49_BF_24_MoNPS): C (53.87, 54.04), H (3.31, 3.46), N (0.94, 0.97).

**[Cp*Mo(1,2-Ph_2_PC_6_H_4_S(CH=CH)_2_CH=CH_2_)(HC≡CH)_2_][BAr^F^_4_]**, **3.** In a 25 mL Schlenk tube charged with a magnetic stir bar, **2** (10 mg, 0.007 mmol) was dissolved in 5 mL of THF. The tube was taken out from the glovebox and immersed in a liquid nitrogen bath. The solution was frozen and gently degassed through freeze-pump-thaw cycle and then pressurized with 1 atm of C_2_H_2_. After reaction at room temperature for 1 h, the solution turned to yellow, and a large amount of black solid was precipitated. The PA product (48 mg) was isolated by filtration and washed with THF (5 mL × 3), and then dried under vacuum. The filtrate was concentrated and then layered with hexane. The resulting solution was stored at -30 ℃, which produced yellow crystals of complex **3** (6 mg, 59% yield). Single crystals suitable for X-ray diffraction were obtained by liquid to liquid diffusion of hexane into a PhF solution of **3** at -30 ℃. **^1^H NMR** (500 MHz, *d*_8_-THF, ppm) 10.66 (s, 1H), 10.46 (s, 1H), 8.99 (d, *J* = 17.8 Hz, 1H), 8.73 (d, *J* = 18.7 Hz, 1H), 7.79 (s, 8H), 7.74 (s, 2H), 7.58 (s, 9H), 7.54 (m, 2H), 7.43 (m, 3H), 7.32 (m, 2H), 6.74 (m, 2H), 6.23 (d, *J* = 14.8 Hz, 1H), 5.96 (m, 2H), 5.23 (d, *J* = 16.7 Hz, 1H), 5.12 (d, *J* = 10.0 Hz, 1H), 1.61 (s, 15H). **^13^C{^1^H} NMR** (126 MHz, *d*_8_-THF, ppm) 179.8, 176.8, 162.6 (q, *J* = 50.2 Hz), 150.4, 148.8, 144.9, 140.3, 137.4, 135.4, 134.6, 133.6, 132.7, 132.0, 131.0, 129.9, 129.7, 129.5, 127.3, 126.4, 124.2, 122.5, 122.1, 119.0, 118.0, 110.9, 10.7. **^31^P{^1^H} NMR** (202 MHz, *d_8_*-THF, ppm) 61.7. **HRMS (m/z)**: [**3**]^+^ calcd for C_38_H_40_MoPS, 657.1642; found, 657.1635. **Analysis** (calcd., found for C_70_H_52_BF_24_MoPS): C (55.35, 54.26), H (3.45, 3.71).

**[Cp*Mo(H)_2_(1,2-Ph_2_PC_6_H_4_SCH_2_CH_3_)][BAr^F^_4_], 4.** A solution of **2** (25 mg, 0.017 mmol**)** in *d_8_*-THF (0.6 mL) was added to a J. Young tube. The J. Young tube was taken out from the glovebox and immersed in a liquid nitrogen bath, and gently degassed under vacuum. The solution was then warmed up to room temperature and pressurized with H_2_ gas (1 atm). After the reaction for 12 h, the sample was directly subjected to NMR analysis. After NMR analysis, the resulting solution was transferred to a small reaction vial (5 mL) and layered with hexane, and then stored at -30 ℃ for several days. Compound **4** was obtained as orange crystals. Yield: 17 mg, 68%. **^1^H NMR** (500 MHz, C_6_D_5_Cl, 253 K, ppm) 8.70 (m, 1H), 8.37 (m, 8H), 7.72 (s, 1H), 7.55 (m, 6H), 7.42 (s, 2H), 7.36 (m, 1H), 7.22 (m, 4H), 7.06 (m, 2H), 6.91 (d, *J* = 7.4 Hz, 2H), 6.80 (m, 2H), 6.63 (m, 1H), 6.28 (m, 1H), 5.16 (dd, *J*_H-H_ = 110 Hz, *J*_P-H_ = 55 Hz, 1H), 3.42 (m, 1H), 2.56 (m, 1H), 1.17 (m, 3H), 1.11 (s, 15H), -4.19 (d, *J*_H-H_ = 110 Hz, 1H). **^31^P{^1^H} NMR** (202 MHz, *d_8_*-THF, 253 K, ppm) 76.1. **^31^P NMR** (202 MHz, *d_8_*-THF, 253 K, ppm) δ 75.7 (d, *J*_P-H_ = 55 Hz). **HRMS (**m/z): [**4**]^+^ calcd. for C_35_H_41_MoNPS, 636.1751; found, 636.1735. **Analysis** (calcd., found for C_67_H_53_BF_24_MoNPS): C (53.72, 53.41), H (3.57, 3.55), N (0.94, 0.91).

### Optimization Studies

In an N_2_-filled glovebox, to a J. Young NMR tube charged with catalyst (1-2 mol%), **5a** (0.2 mmol) in *d*_8_-[THF](javascript:;) (0.6 mL), was added 1,3,5-trimethoxybenzene (11.2 mg, 0.067 mmol) as the internal standard. The tube was taken out from the glovebox and immersed in a liquid nitrogen bath, and gently degassed under vacuum. The solution was then warmed up to room temperature and pressurized with H_2_ gas (1 atm). After the indicated time, the yield was calculated by ^1^H NMR using 1,3,5-trimethoxybenzene as internal standard.

**Supplementary Table 1**. Optimization of reaction conditions for the hydrogenation of **5a**.

Reaction conditions: **5a** (0.2 mmol), 1,3,5-trimethoxybenzene (0.067 mmol), H_2_ (1 atm) in 0.6 mL *d*_8_-THF. *^a^*Yields were determined by ^1^H NMR spectroscopy using 1,3,5-trimethoxybenzene as internal standard.

### Catalytic Hydrogenation of Alkenes

In an N_2_-filled glovebox, to a J. Young NMR tube charged with **2** (6 mg, 0.004 mmol), alkenes (0.2 mmol) in *d*_8_-[THF](javascript:;) (0.6 mL), was added 1,3,5-trimethoxybenzene (11.2 mg, 0.067 mmol) as the internal standard. The tube was taken out from the glovebox and immersed in a liquid nitrogen bath, and gently degassed under vacuum. The solution was then warmed up to room temperature and pressurized with H_2_ gas (1 atm). After reaction at room temperature for 12 h, the solution was analyzed by ^1^H NMR to determine the yield of the hydrogenated product.

**1-Ethylbenzene (6a).** NMR yield: 92%. ^1^H NMR (500 MHz, *d_8_*-THF, ppm) 7.21 (t, *J* = 7.5 Hz, 2H), 7.16 (d, *J* = 6.9 Hz, 2H), 7.10 (t, *J* = 7.2 Hz, 1H), 2.61 (q, *J* = 7.6 Hz, 2H), 1.21 (t, *J* = 7.6 Hz, 3H).^2^

**1-Ethyl-4-methylbenzene (6b).** NMR yield: 88%. ^1^H NMR (500 MHz, *d_8_*-THF, ppm) 7.03 (s, 4H), 2.57 (q, *J* = 7.6 Hz, 2H), 2.26 (s, 3H), 1.18 (t, *J* = 7.6 Hz, 3H).^3^

**1-Ethyl-4-methoxybenzene (6c).** NMR yield: 96%. ^1^H NMR (500 MHz, *d_8_*-THF, ppm) 7.03 (s, 4H), 2.57 (q, *J* = 7.6 Hz, 2H), 2.26 (s, 3H), 1.18 (t, *J* = 7.6 Hz, 3H).^3^

**1-Ethyl-4-fluorobenzene (6d).** NMR yield: 77%. ^1^H NMR (500 MHz, *d_8_*-THF, ppm) 7.16 (dd, *J* = 8.7, 5.5 Hz, 2H), 6.96 (t, *J* = 8.8 Hz, 2H), 2.60 (q, *J* = 7.6 Hz, 2H), 1.19 (t, *J* = 7.6 Hz, 3H).^4^

**4-Ethylphenyl acetate (6e).** NMR yield: 59%. ^1^H NMR (500 MHz, *d_8_*-THF, ppm) 7.16 (d, *J* = 8.4 Hz, 2H), 6.96 (d, *J* = 8.4 Hz, 2H), 2.61 (q, *J* = 7.6 Hz, 2H), 2.18 (s, 3H), 1.21 (t, *J* = 7.6 Hz, 3H).^5^

**Methyl 4-ethylbenzoate (6f).** NMR yield: 61%. ^1^H NMR (500 MHz, *d_8_*-THF, ppm) 7.91 (d, *J* = 8.2 Hz, 2H), 7.28 (d, *J* = 8.2 Hz, 2H), 3.83 (s, 3H), 2.69 (q, *J* = 7.6 Hz, 2H), 1.23 (t, *J* = 7.6 Hz, 3H).^3^

**1-Ethyl-4-(trifluoromethyl)benzene (6g).** NMR yield: 47%. ^1^H NMR (500 MHz, *d_8_*-THF, ppm) 7.56 (d, *J* = 8.1 Hz, 2H), 7.37 (d, *J* = 8.0 Hz, 2H), 2.70 (q, *J* = 7.6 Hz, 2H), 1.24 (t, *J* = 7.6 Hz, 3H).^6^

**1-Ethyl-3-methylbenzene (6h).** NMR yield: 97%. ^1^H NMR (500 MHz, *d_8_*-THF, ppm) 7.09 (t, *J* = 7.5 Hz, 1H), 6.98 (s, 1H), 6.93 (t, *J* = 6.8 Hz, 2H), 2.57 (q, *J* = 7.6 Hz, 2H), 1.19 (t, *J* = 7.6 Hz, 3H).^6^

**Methyl-3-ethylbenzoate (6i).** NMR yield: 70%. ^1^H NMR (500 MHz, *d_8_*-THF, ppm) 7.81 (d, 2H), 7.43 – 7.32 (m, 2H), 2.69 (q, *J* = 7.6 Hz, 2H), 1.23 (t, *J* = 7.6 Hz, 3H).^7^

**1-Ethyl-3-fluorobenzene (6j).** NMR yield: 82%. ^1^H NMR (500 MHz, *d_8_*-THF, ppm) 7.22 (m, 1H), 6.97 (d, *J* = 7.6 Hz, 1H), 6.92 (d, *J* = 10.2 Hz, 1H), 6.85 (m, 1H), 2.63 (q, *J* = 7.6 Hz, 2H), 1.21 (t, *J* = 7.6 Hz, 3H).^8^

**1-Ethyl-3-(trifluoromethyl)benzene (6k).** NMR yield: 92%. ^1^H NMR (500 MHz, *d_8_*-THF, ppm) 7.51 (s, 1H), 7.47 – 7.41 (m, 3H), 2.71 (q, *J* = 7.6 Hz, 2H), 1.24 (t, *J* = 7.6 Hz, 3H).

**2,4-Dimethylethylbenzen (6l).** NMR yield: 97%. ^1^H NMR (500 MHz, *d_8_*-THF, ppm) 6.97 (d, *J* = 7.6 Hz, 1H), 6.91 – 6.84 (m, 2H), 2.56 (q, *J* = 7.5 Hz, 2H), 2.22 (s, 6H), 1.15 (t, *J* = 7.6 Hz, 3H).^9^

**Cumene (6m).** NMR yield: 20%. ^1^H NMR (500 MHz, *d_8_*-THF, ppm) 7.20 (m, 3H), 7.10 (m, 2H), 2.87 (m, *J* = 6.9 Hz, 1H), 1.23 (d, *J* = 6.9 Hz, 6H).^6^

**1-Propylbenzene (6n).** NMR yield: 91%. ^1^H NMR (500 MHz, *d_8_*-THF, ppm) 7.21 (t, *J* = 7.5 Hz, 2H), 7.16 – 7.07 (m, 3H), 2.63 – 2.50 (m, 2H), 1.68 – 1.57 (m, 2H), 0.92 (t, *J* = 7.4 Hz, 3H).^10^

**2-Ethylthiophene (6o)**. NMR yield: 84%. ^1^H NMR (500 MHz, *d_8_*-THF) 7.15 (m, 1H), 6.89 (m, 1H), 6.83 – 6.78 (m, 1H), 2.87 (q, *J* = 7.5 Hz, 2H), 1.31 (t, *J* = 7.5 Hz, 3H).^6^

**Ethylferrocene (6p).** NMR yield: 92%. ^1^H NMR (500 MHz, *d_8_*-THF, ppm) 4.06 (s, 5H), 4.04 – 4.02 (m, 2H), 4.00 – 3.98 (m, 2H), 2.33 (q, *J* = 7.5 Hz, 2H), 1.16 (t, *J* = 7.5 Hz, 3H).^11^

**9-Ethyl-9H-carbazole (6q)**. NMR yield: 99%. ^1^H NMR (500 MHz, *d_8_*-THF, ppm) 8.06 (d, *J* = 7.8 Hz, 2H), 7.45 (m, 2H), 7.42 – 7.36 (m, 2H), 7.18 – 7.12 (m, 2H), 4.39 (q, *J* = 7.2 Hz, 2H), 1.37 (t, *J* = 7.2 Hz, 3H).^3^

**Ethyldimethyl(phenyl)silane (6r)**. NMR yield: 85%. ^1^H NMR (500 MHz, *d_8_*-THF, ppm) 7.49 (d, *J* = 9.4 Hz, 2H), 7.30 (d, *J* = 9.8 Hz, 3H), 0.95 (t, *J* = 7.8 Hz, 3H), 0.74 (q, *J* = 7.8 Hz, 2H), 0.24 (s, 6H). ^29^Si NMR (99 MHz, *d_8_*-THF) -1.94.^3^

 **(Ethylsulfonyl)benzene (6s).** NMR yield: 38%. ^1^H NMR (500 MHz, *d_8_*-THF, ppm) 7.87 (m, 2H), 7.66 (m, 1H), 7.59 – 7.54 (m, 2H) 3.09 (q, *J* = 7.4 Hz, 2H), 1.16 (t, *J* = 7.4 Hz, 3H).^3^

**Propyloxybenzene (6t).** NMR yield: 98%. ^1^H NMR (500 MHz, *d_8_*-THF, ppm) 7.24 – 7.17 (m, 2H), 6.88 – 6.82 (m, 3H), 3.88 (t, *J* = 6.5 Hz, 2H), 1.76 (m, 2H), 1.02 (t, *J* = 7.4 Hz, 3H).^12^

**Myristicin (6u)**. NMR yield: 98%. ^1^H NMR (500 MHz, *d_8_*-THF, ppm) 6.35 (s, 1H), 6.33 (s, 1H), 5.83 (s, 2H), 3.82 (s, 3H), 2.50 – 2.43 (m, 2H), 1.63 – 1.54 (m, 2H), 0.91 (t, *J* = 7.4 Hz, 3H).^5^

**2-Ethyl-4,4,5,5-tetramethyl-1,3,2-dioxaborolane (6v).** NMR yield: 97%. ^1^H NMR (500 MHz, *d_8_*-THF, ppm) 1.44 – 1.37 (m, 2H), 1.19 (s, 12H), 0.90 (t, *J* = 7.4 Hz, 3H), 0.68 (t, *J* = 7.5 Hz, 2H). ^11^B NMR (160MHz, THF) *δ* 33.93.^13^

**N-Phenylpentanamide (6w).** NMR yield: 66%. ^1^H NMR (500 MHz, *d_8_*-THF, ppm) 8.90 (br, 1H), 7.60 (m, 2H), 7.23 – 7.15 (m, 2H), 6.95 (m, 1H), 2.28 (m, 2H), 1.69 – 1.59 (m, 2H), 1.37 (m, 2H), 0.92 (m, 3H).^14^

**Methyl hexanoate (6x).** NMR yield: 75%. ^1^H NMR (500 MHz, *d_8_*-THF, ppm) 3.62 (s, 3H), 2.31 (t, *J* = 7.8 Hz, 2H), 1.67 – 1.57 (m, 2H), 1.40 – 1.29 (m, 4H), 0.93 (t, *J* = 7.0 Hz, 3H).^15^

**5,5'-dipropyl-[1,1'-biphenyl]-2,2'-diol (6y).** NMR yield: 87%. ^1^H NMR (500 MHz, *d_8_*-THF, ppm) 8.17 (s, 2H), 7.07 (d, *J* = 2.2 Hz, 2H), 7.00 (m, 2H), 6.80 (d, *J* = 8.2 Hz, 2H), 2.58 – 2.45 (m, 4H), 1.67 – 1.57 (m, 4H), 0.94 (t, *J* = 7.3 Hz, 6H).^16^

**Hexan-1-ol (6z).** NMR yield: 83%. ^1^H NMR (500 MHz, *d_8_*-THF, ppm) 3.46 (t, *J* = 6.5 Hz, 2H), 1.46 – 1.50 (m, 2H), 1.39 – 1.22 (m, 6H), 0.90 (t, *J* = 6.6 Hz, 3H).^17^

**2-Oxiranepropanol (6aa).** NMR yield: 96%. ^1^H NMR (500 MHz, *d_8_*-THF, ppm) 3.02 (s, 1H), 1.91 – 1.74 (m, 3H), 1.70 – 1.40 (m, 4H), 1.11 (dd, *J* = 15.0, 5.6 Hz, 6H), 1.05 (d, *J* = 5.1 Hz, 3H), 0.89 (q, *J* = 7.4 Hz, 3H).^18^

**Ethylcyclohexane (6ab).** NMR yield: 68%. ^1^H NMR (500 MHz, *d_8_*-THF, ppm) 1.72 (m, *J* = 8.5 Hz, 5H), 1.23 – 1.18 (m, 6H), 0.87 (m, *J* = 7.4 Hz, 5H).^19^

**Octane (6ac).** NMR yield: 88%. ^1^H NMR (500 MHz, *d_8_*-THF, ppm) 1.29 (s, 12H), 0.89 (t, *J* = 6.9 Hz, 6H).^6^

**Estr-4-en-17-ol (6ad).** Eluent: petroleum ether/EtOAc (10:1). White solid. Isolated yield: 540.6 mg, 90%. ^1^H NMR (500 MHz, CDCl_3_, ppm) 5.38 (s, 1H), 2.20 – 2.17 (m, 1H), 2.02 – 1.91 (m, 5H), 1.83 – 1.80 (m, 1H), 1.75 – 1.74 (m, 1H), 1.68– 1.66 (m, 2H), 1.54 – 1.43 (m, 6H), 1.39 – 1.04 (m, 10H), 0.95 – 0.92 (t, *J* = 6.9 Hz, 3H), 0.89 (s, 3H), 0.65 – 0.57 (m, 1H).^20^

***d*_2_-Estr-4-en-17-ol (*d*_2_-6ad).** Eluent: petroleum ether/EtOAc (10:1). White solid. Isolated yield: 98.3 mg, 91%. ^1^H NMR (500 MHz, CDCl_3_, ppm) 5.37 (s, 1H), 2.20 – 2.17 (m, 1H), 2.02 – 1.90 (m, 5H), 1.83 – 1.80 (m, 1H), 1.75 – 1.74 (m, 1H), 1.68– 1.66 (m, 2H), 1.54 – 1.43 (m, 5H), 1.37 – 1.04 (m, 10H), 0.92 – 0.90 (t, *J* = 6.9 Hz, 2H), 0.89 (s, 3H), 0.65 – 0.57 (m, 1H). ^2^H NMR (77 MHz, CHCl_3_, ppm) *δ* 1.45, 0.94.

### Hydrogenation of a mixture of 2-octene and 4-octene

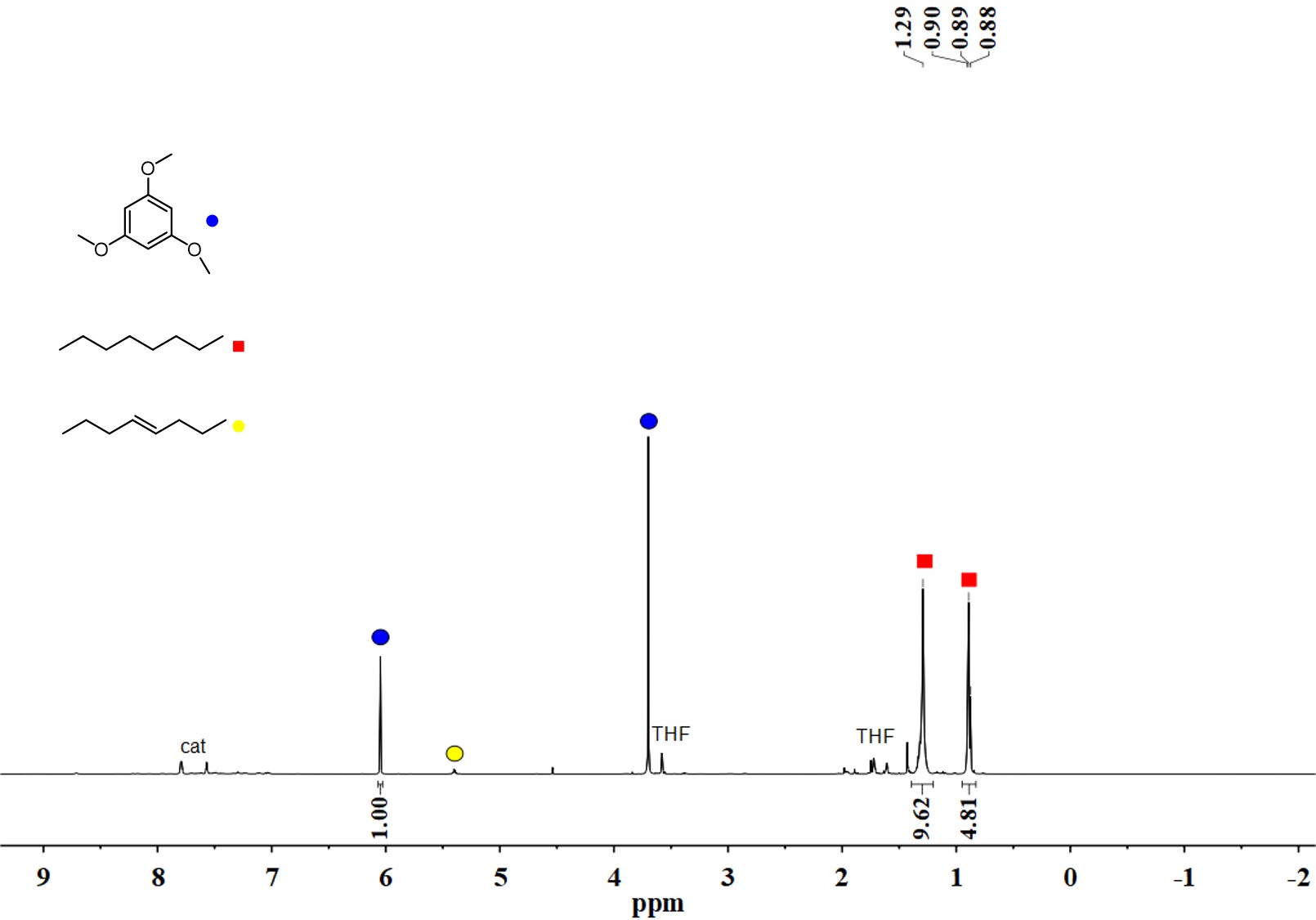


**Supplementary Figure 1.** ^1^H NMR (500 MHz, *d_8_*-THF) spectrum of the hydrogenation of 2-octene and 4-octene (*trans* and *cis*) reaction mixture.

### Scale-Up Experiment


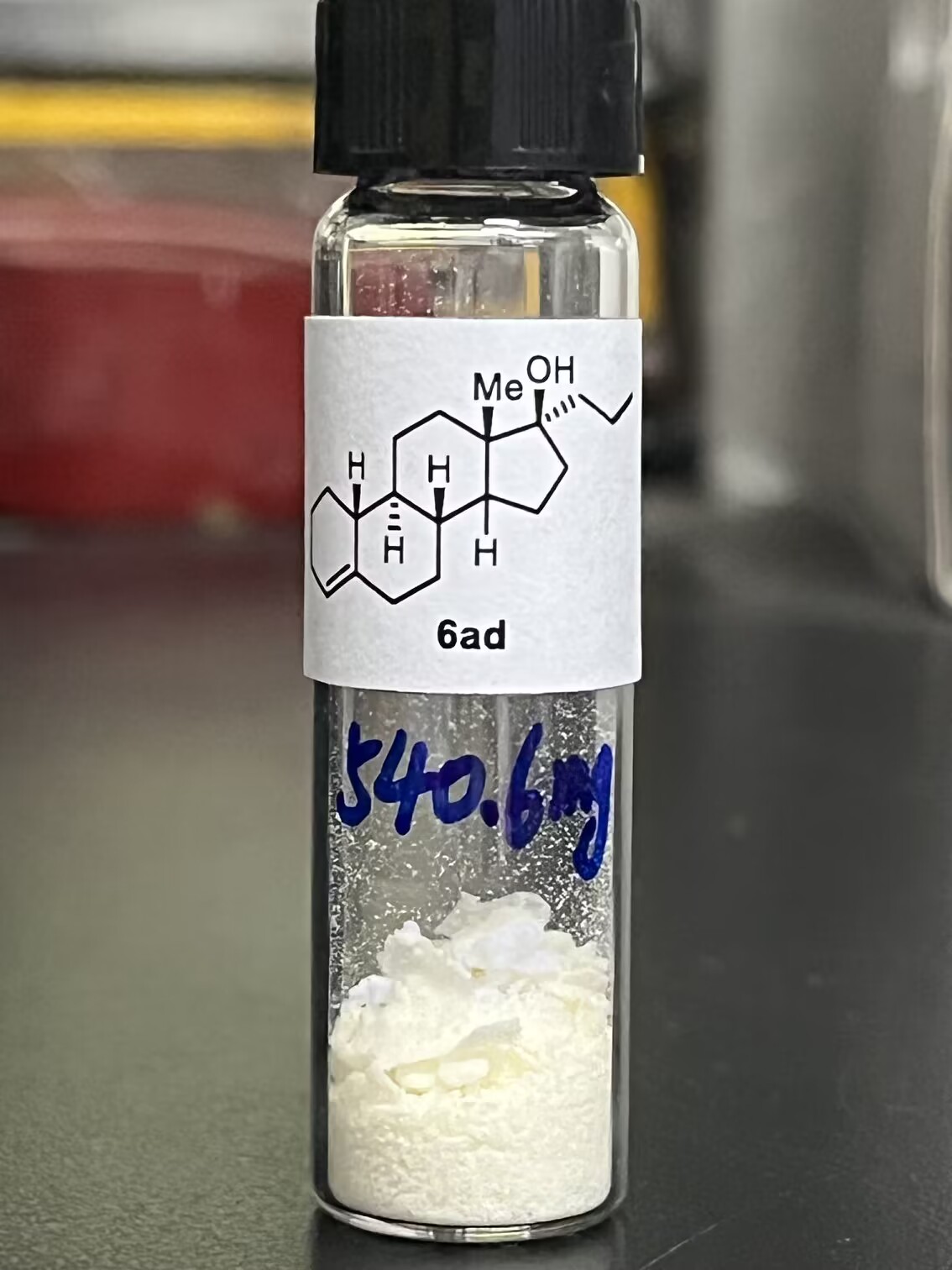

In an N_2_-filled glovebox, a flame-dried Schlenk tube, was charged with catalyst **2** (149.6 mg, 2 mol%), **5ad** (601.0 mg, 2.0 mmol) in *d*_8_-THF (3.0 mL). The tube was taken out from the glovebox and immersed in a liquid nitrogen bath, and gently degassed under vacuum. The solution was then warmed up to room temperature and pressurized with H_2_ gas (1 atm). After reaction at room temperature for 12 h, the mixture was evaporated to dryness, and the crude product was purified by column chromatograph on silica gel eluting with petroleum ether/EtOAc (10: 1) to give the corresponding product **6ad (**white solid, 540.6 mg, 90%).

The experimental procedure worked for the reaction of **5ad** with D_2_ is the same as that described for **5ad** with H_2_. The deuterated product was confirmed by the ^2^H NMR spectrum (Supplementary Figure 60).

### Catalytic Hydrogenation of 5a by Complex 4

In an N_2_-filled glovebox, to a J. Young NMR tube charged with **4** (6 mg, 0.004 mmol), **5a** (0.2 mmol) in *d*_8_-[THF](javascript:;) (0.6 mL), was added 1,3,5-trimethoxybenzene (11.2 mg, 0.067 mmol) as the internal standard. The tube was taken out from the glovebox and immersed in a liquid nitrogen bath, and gently degassed under vacuum. The solution was then warmed up to room temperature and pressurized with H_2_ gas (1 atm). After reaction at room temperature for 12 h, the solution was analyzed by ^1^H NMR to determine the yield of the hydrogenated product.

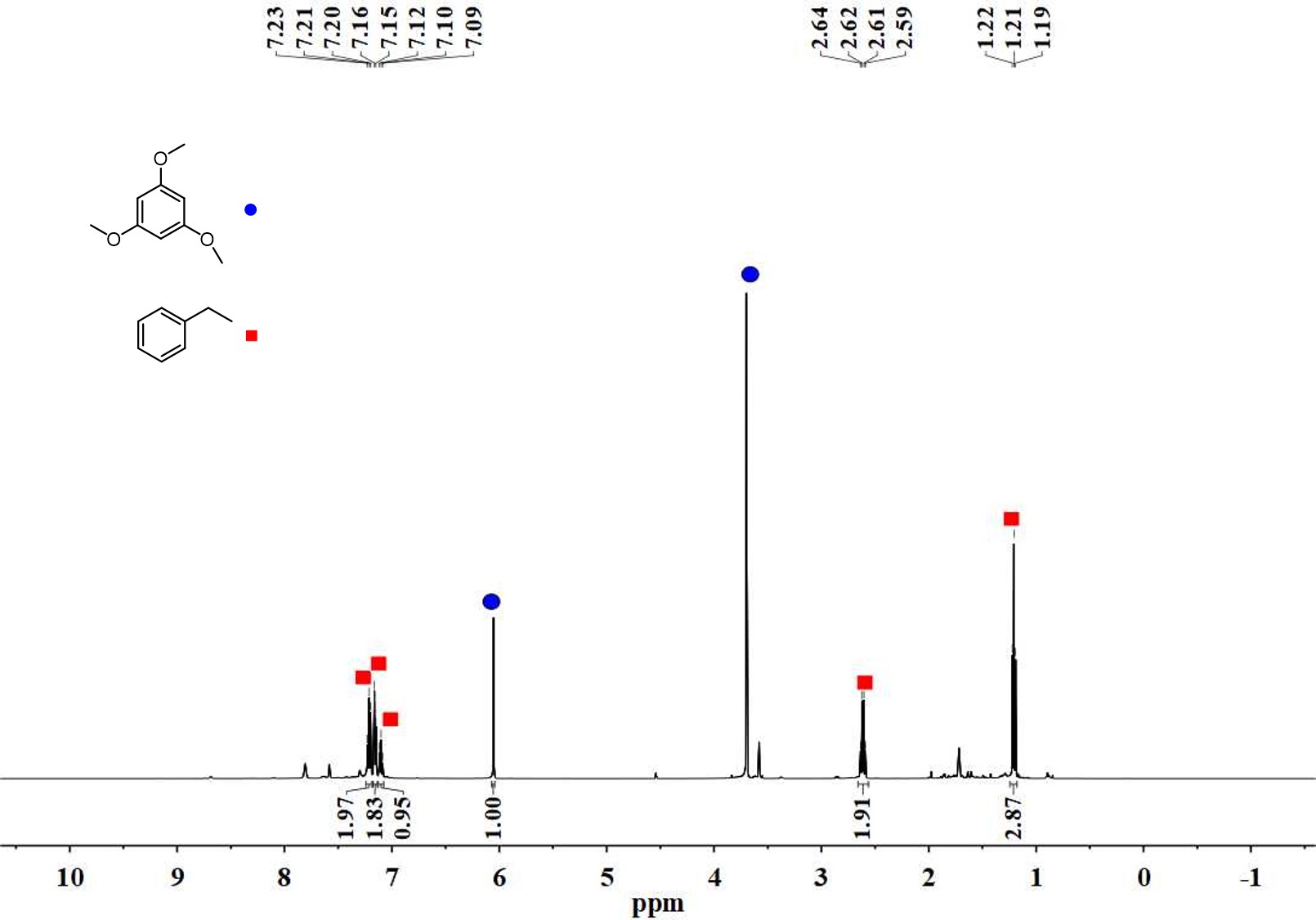


**Supplementary Figure 2.** ^1^H NMR (500 MHz, *d_8_*-THF) spectrum recorded for the hydrogenation of **5a** catalyzed by **4**.

### Deuterium Labeling Experiment

In an N_2_-filled glovebox, two J. Young NMR tubes were charged with **2** (6 mg, 0.004 mmol), **5a** (0.2 mmol) and 1,3,5-trimethoxybenzene (11.2 mg, 0.067 mmol) in *d*_8_-[THF](javascript:;) and THF (0.6 mL), respectively. The tubes were taken out from the glovebox and immersed in a liquid nitrogen bath, and gently degassed under vacuum. The solutions were then warmed up to room temperature and pressurized with D_2_ gas (1 atm). After reaction at room temperature for 12 h, the solutions were analyzed by ^1^H NMR and ^2^H NMR spectroscopy.


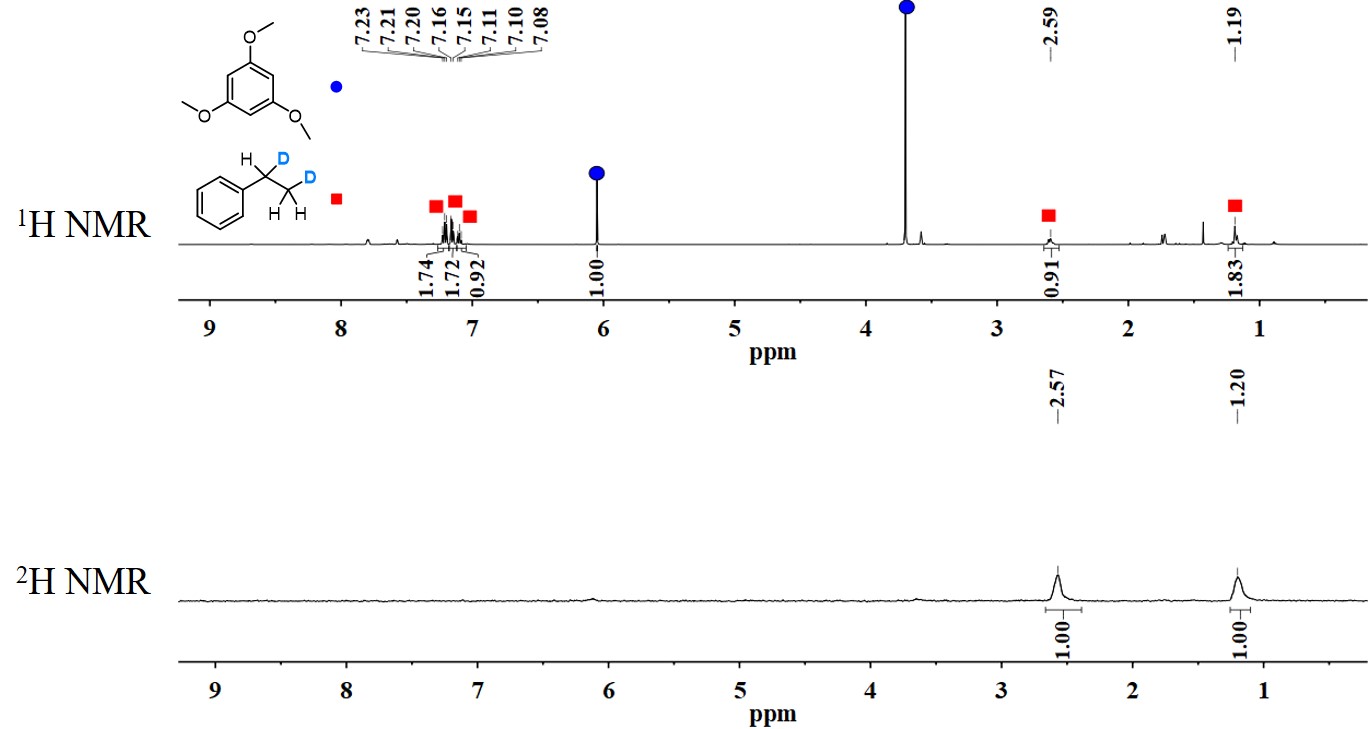


**Supplementary Figure 3.** ^1^H NMR (500 MHz, *d_8_*-THF) and ^2^H NMR (77 MHz, THF) spectra recorded for the hydrogenation of **5a** with H_2_ and D_2_ catalyzed by **2**.

### Determination of the Kinetic Isotope Effect

KIE was determined using standard condition, data points were collected at 900 s intervals over 4 h at room temperature, data points before 20% conversion were subjected to the linear regression analysis.

| **2** | **5a** | 1,3,5-trimethoxybenzene |
| --- | --- | --- |
| 0.0040 mmol  0.0067 M | 0.20 mmol  0.33 M | 0.067 mmol  0.111 M |


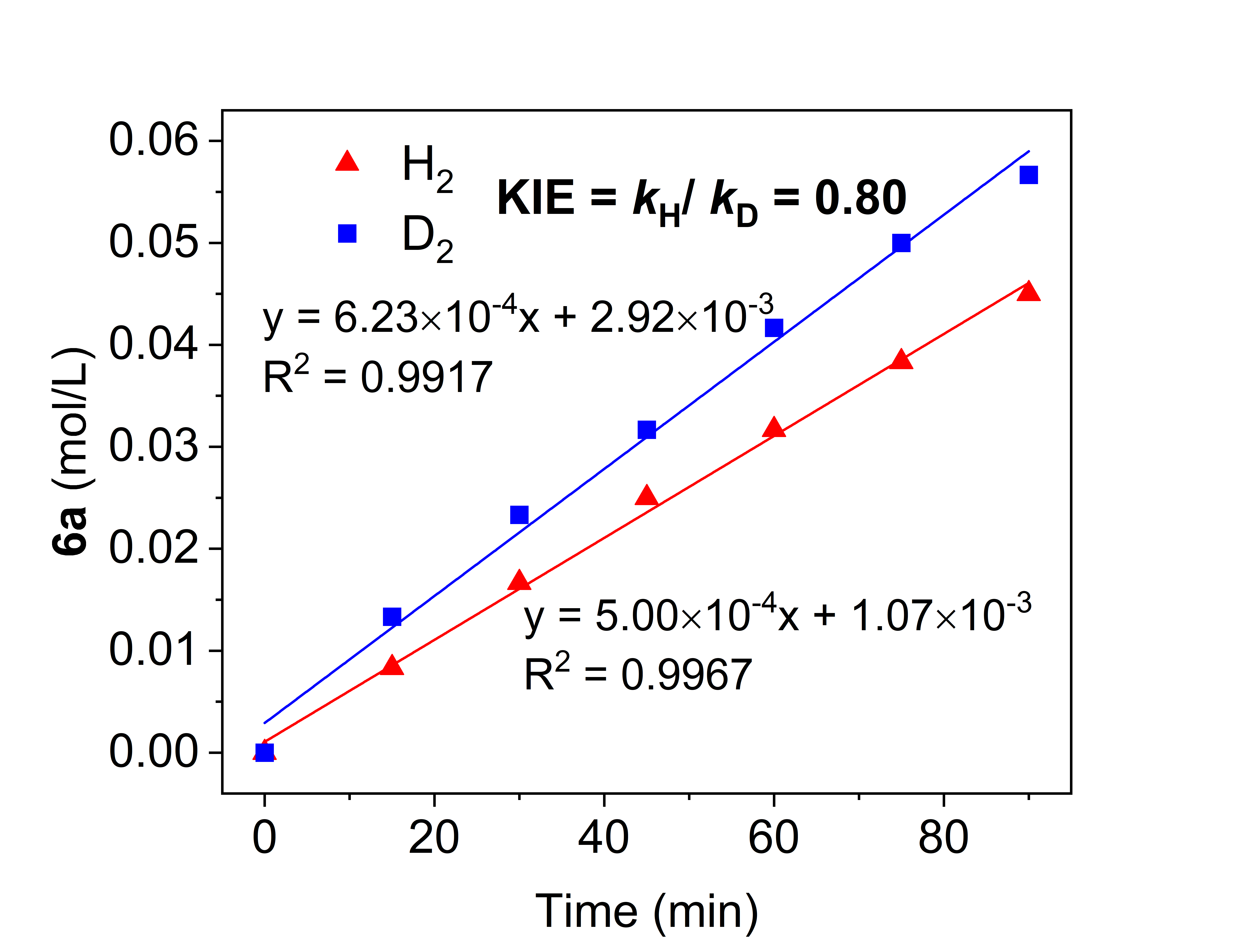


**Supplementary Figure 4.** KIE for hydrogenation of **5a**.


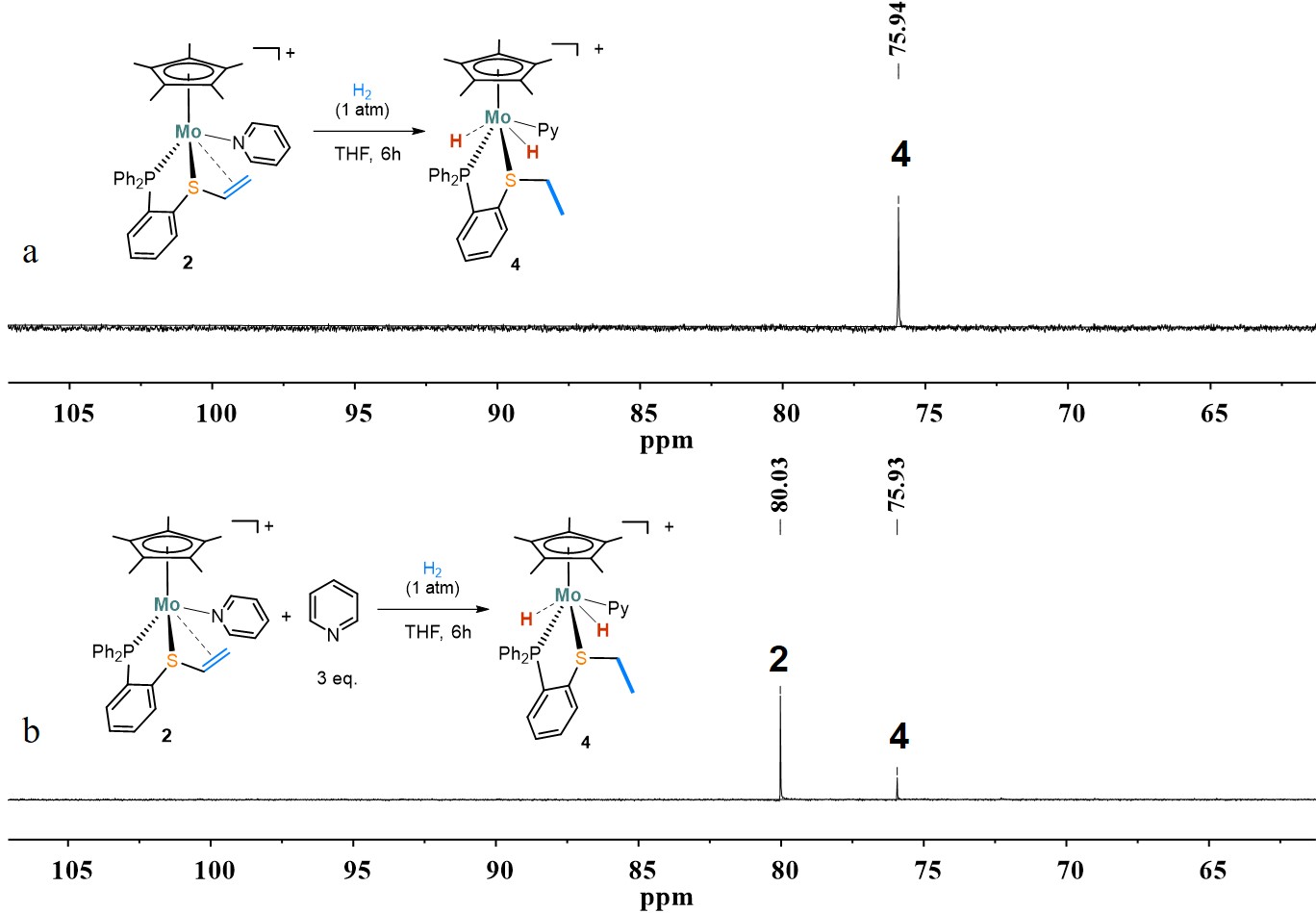


**Supplementary Figure 5.** ^31^P{^1^H} NMR (202 MHz, *d_8_*-THF) spectra for (a) the reaction of **2** with H_2_, and (b) the reaction of **2** with H_2_ in the presence of pyridine (3 equiv).

*Note: These results indicated that the reaction of* ***2*** *with H_2_ is inhibited by pyridine.*


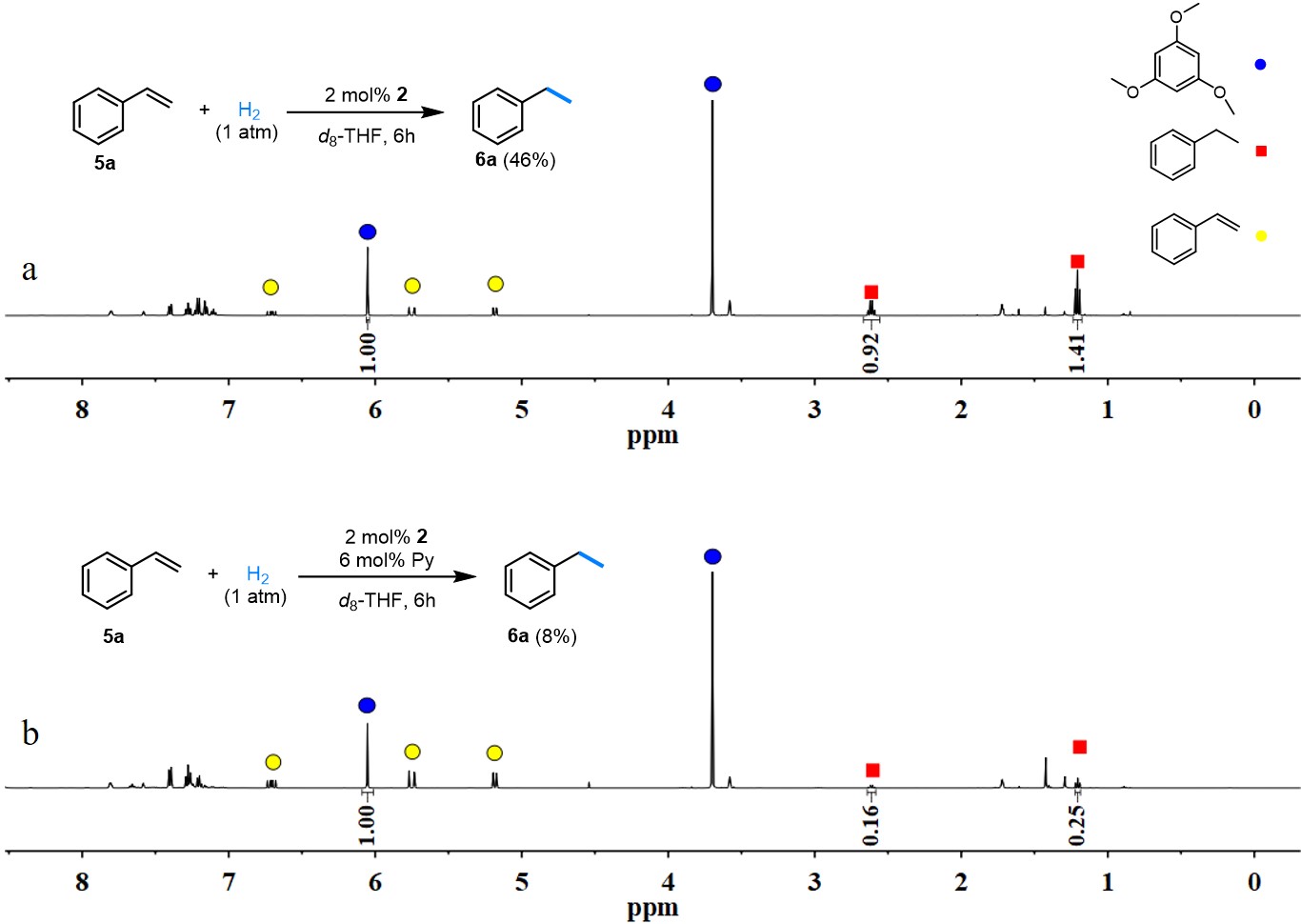


**Supplementary Figure 6.** ^1^H NMR (500 MHz, *d_8_*-THF) spectra recorded for the (a) hydrogenation of **5a** and (b) hydrogenation of **5a** in the presence of pyridine.

*Note: These results indicate that additional pyridine retards the rate of catalytic hydrogenation.*

Synthesis of complexes **2** and **2′**: Under N_2_ atmosphere, a green solution of **1-**C_2_H_2_ (30 mg, 0.054 mmol**)** in 3 mL of THF was cooled to -20 ℃. The resulting solution was treated with the corresponding pyridylic acid derivatives (0.053 mmol) in 2 mL of THF, which caused a change of the solution color from green to brown. After the removal of the solvent under vacuum, the solid was washed with hexane (5-10 mL). The corresponding complexes **2** and **2′** were precipitated and separately isolated as brown powder.

Hydrogenation of **5a** catalyzed by **2** and **2′**: In an N_2_-filled glovebox, to three J. Young NMR tubes charged separately with catalyst **2** and **2′** (0.004 mmol), **5a** (0.2 mmol) in *d*_8_-[THF](javascript:;) (0.6 mL), was added 1,3,5-trimethoxybenzene (11.2 mg, 0.067 mmol) as the internal standard. The tube was taken out from the glovebox and immersed in a liquid nitrogen bath, and gently degassed under vacuum. The solution was then warmed up to room temperature and pressurized with H_2_ gas (1 atm). After reaction at room temperature for 3 h, the solution was analyzed by ^1^H NMR to determine the yield of **6a**.


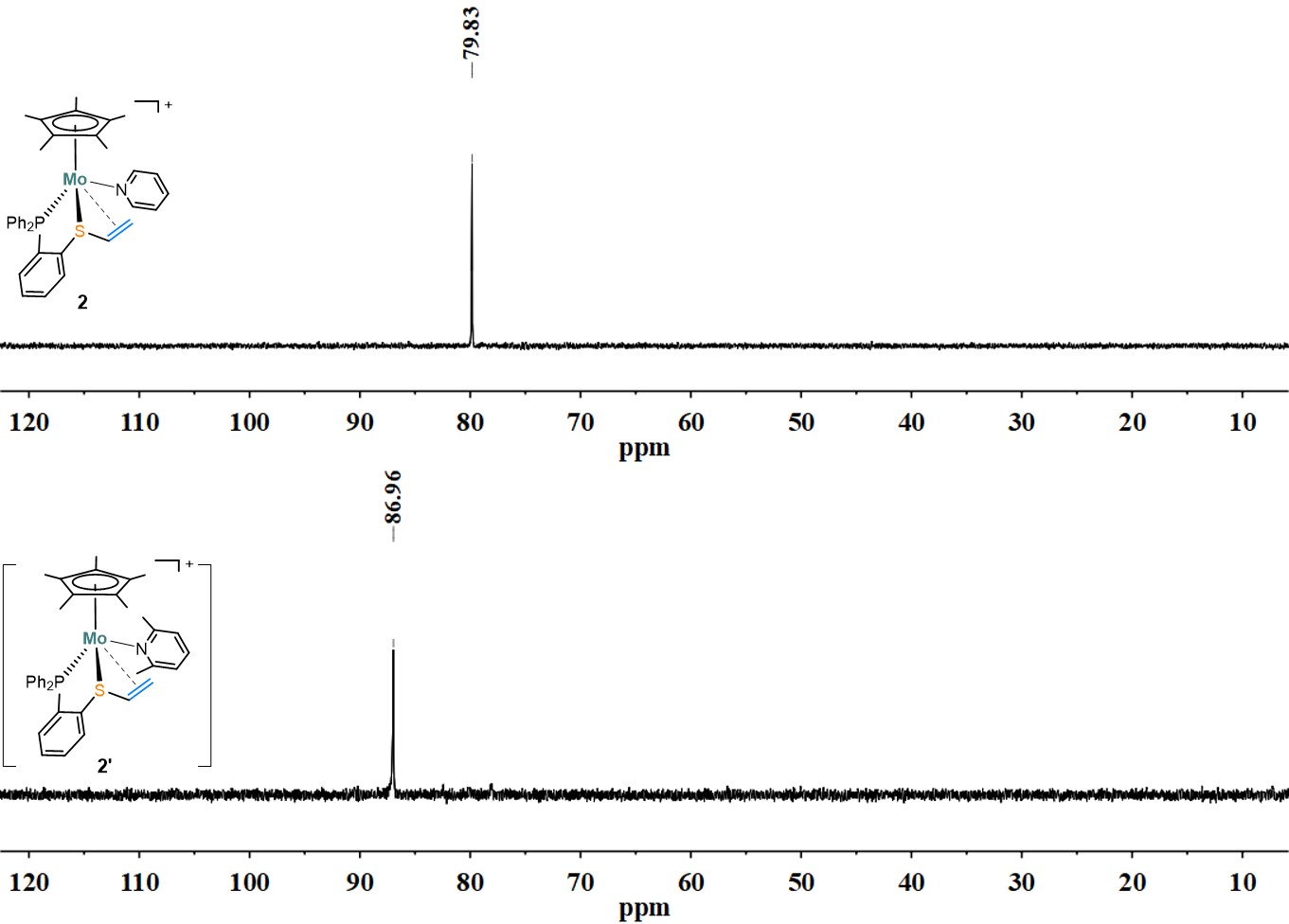


**Supplementary Figure 7.** ^31^P{^1^H} NMR spectrum of **2** and **2’**.


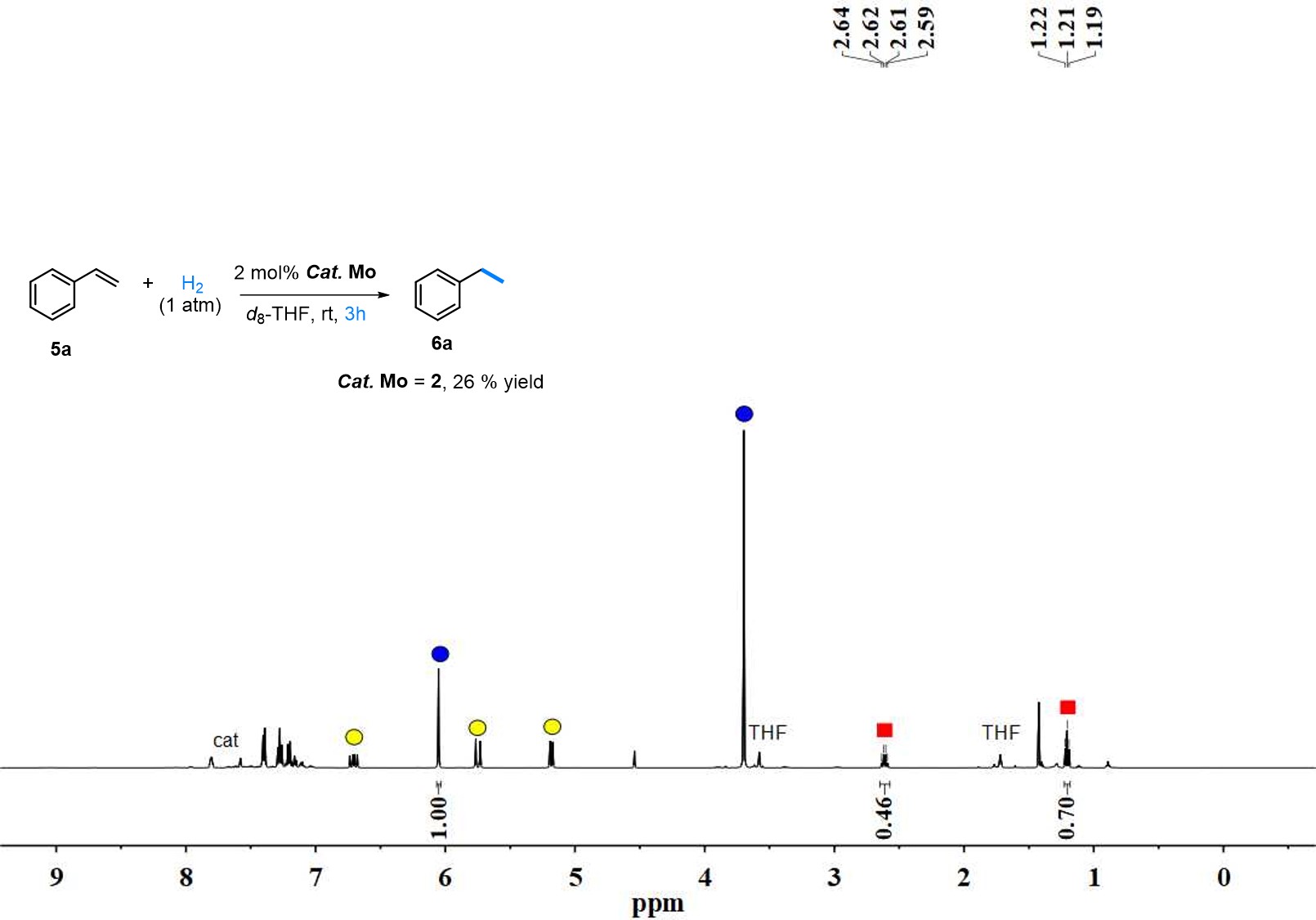


**Supplementary Figure 8.** ^1^H NMR (500 MHz, *d_8_*-THF) spectrum recorded for the hydrogenation of **5a** catalyzed by **2** for 3h.


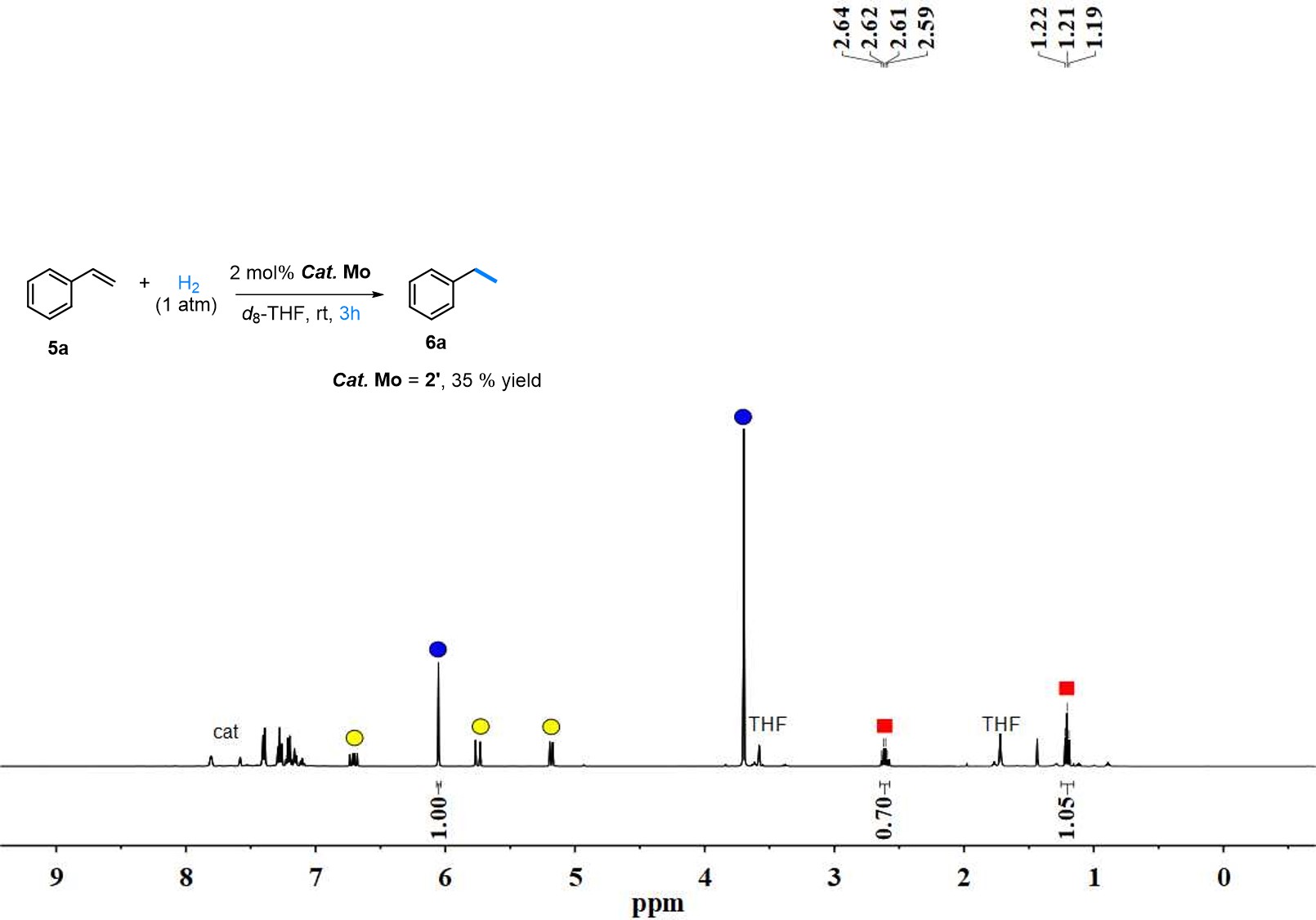


**Supplementary Figure 9.** ^1^H NMR (500 MHz, *d_8_*-THF) spectrum recorded for the hydrogenation of **5a** catalyzed by **2′** for 3h.

### The Hammett-Analysis

Under a nitrogen atmosphere, the corresponding alkenes **5a**-**5g** (0.2 mmol), **2** (6 mg, 0.004 mmol) and 1,3,5-trimethoxybenzene (11.2 mg, 0.067 mmol) were added to J. Young NMR tubes. The tubes were taken out from the glovebox and immersed in a liquid nitrogen bath, and gently degassed under vacuum. The solution was then warmed up to room temperature and pressurized with H_2_ (1 atm). After reaction at room temperature for 4 h, the yields were determined by ^1^H NMR using 1,3,5-trimethoxybenzene as the internal standard.

**Hammett equation in catalytic hydrogenation:**

$$\log\left( \frac{\boldsymbol{k}_{\boldsymbol{X}}}{\boldsymbol{k}_{\boldsymbol{H}}} \right)\boldsymbol{= -0.64}\boldsymbol{\sigma}$$

**Supplementary Table 2. Data for Hammett-plot.**

| Entry | Substituent X | σ | kx | k_X_/k_H_ | log (k_X_/k_H_) |
| --- | --- | --- | --- | --- | --- |
| 1 | *p-*OMe | -0.27 | 3.88×10^-4^ | 1.23 | 0.09 |
| 2 | *p-*Me | -0.17 | 3.46×10^-4^ | 1.10 | 0.04 |
| 3 | *p-*H | 0 | 3.15×10^-4^ | 1 | 0 |
| 4 | *p-*F | 0.06 | 2.67×10^-4^ | 0.85 | -0.07 |
| 5 | *p-*OCOMe | 0.31 | 1.60×10^-4^ | 0.51 | -0.29 |
| 6 | *p-*COOMe | 0.45 | 1.45×10^-4^ | 0.46 | -0.34 |
| 7 | *p-*CF_3_ | 0.54 | 3.89×10^-4^ | 0.40 | -0.39 |


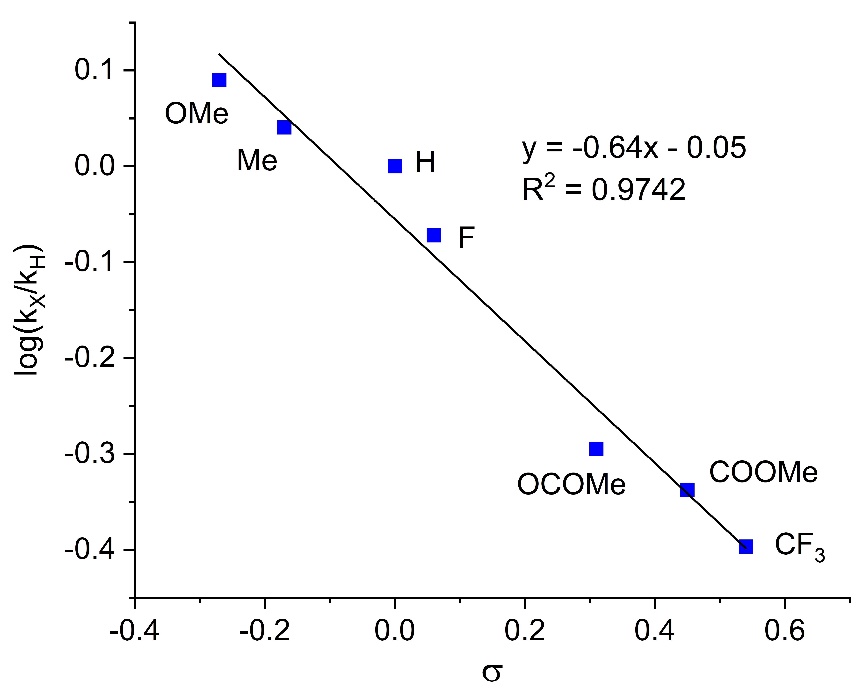


**Supplementary Figure 10. Hammett-plot for hydrogenation.**

## Supplementary Data

### HRMS Spectra

(a)
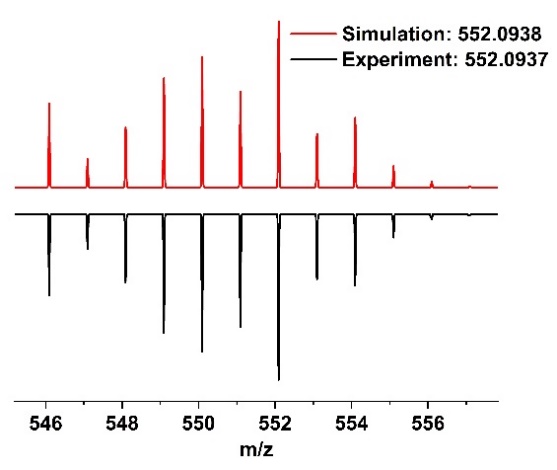
 (b)
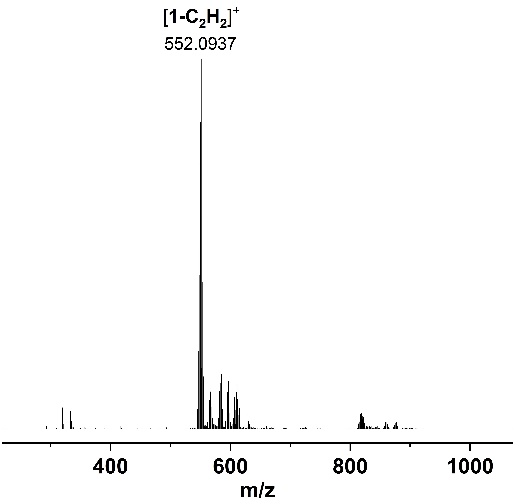


**Supplementary Figure 11.** HRMS spectrum of **1-**C_2_H_2_. (a) the zoom in mass spectrum of **1**-C_2_H_2_. (b) the whole mass spectrum of **1**-C_2_H_2_.

*Result:* calcd for C_30_H_31_MoPS ([**1-**C_2_H_2_]^+^): 552.0938; found, 552.0937.

(a)
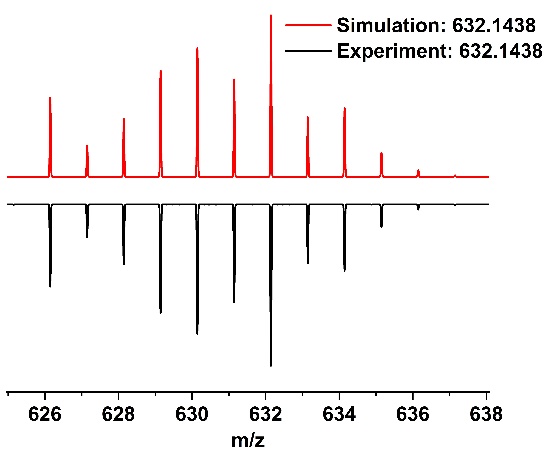
 (b)
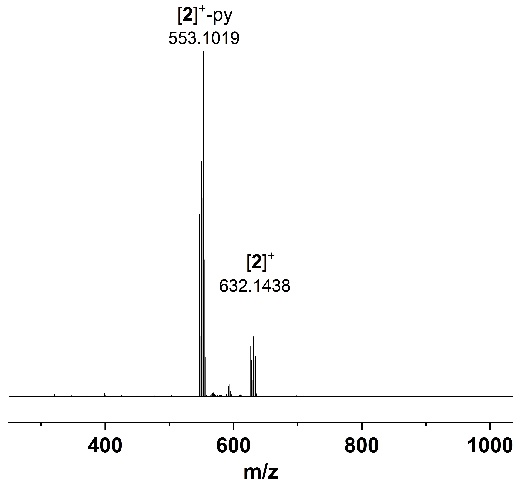


**Supplementary Figure 12.** HRMS spectrum of **2**. (a) the zoom in mass spectrum of **2**. (b) the whole mass spectrum of **2**. *Note:* the ionic peak at 553.1019 corresponds to [Cp*Mo(1,2-Ph_2_PC_6_H_4_S−CH=CH_2_)]^+^ resulting from **2** with the loss of a pyridyl fragment.

*Result:* calcd for C_35_H_37_MoNPS ([**2**]^+^): 632.1438; found, 632.1438.

(a)
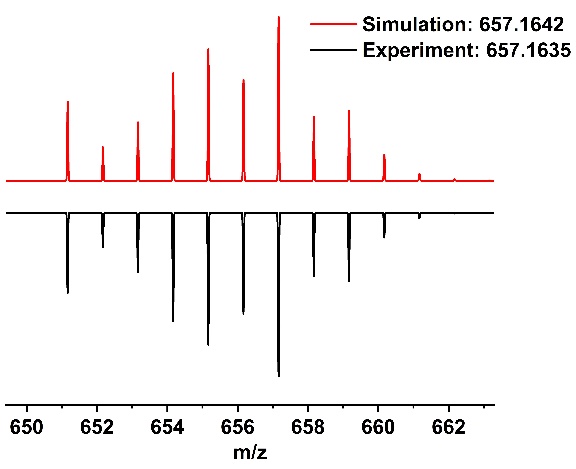
 (b)
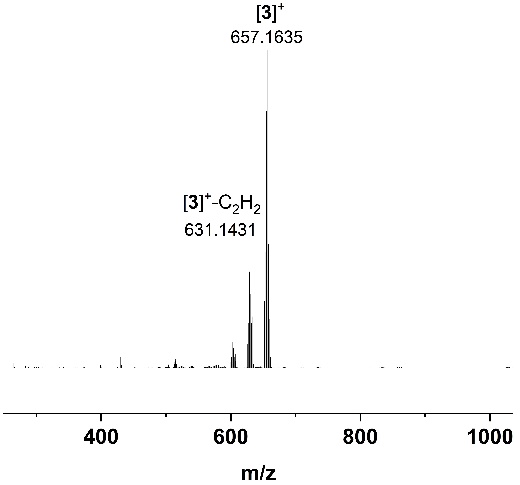


**Supplementary Figure 13.** HRMS spectrum of **3**. (a) the zoom in mass spectrum of **3**. (b) the whole mass spectrum of **3**. *Note:* the ionic peak at 631.1431 corresponds to the species resulting from **3** with the loss of a C_2_H_2_ fragment.

*Result:* calcd for C_38_H_40_MoPS ([**3**]^+^): 657.1642; found, 657.1635.

(a)
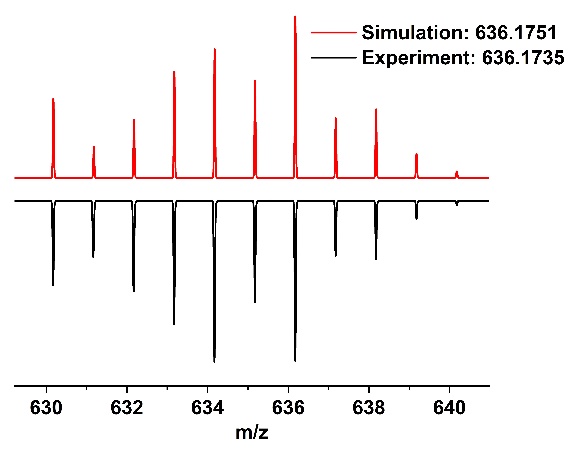
 (b)
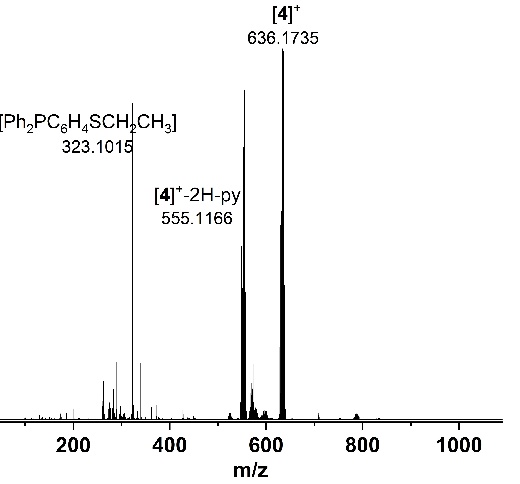


**Supplementary Figure 14.** HRMS spectrum of **4**. (a) the zoom in mass spectrum of **4**. (b) the whole mass spectrum of **4**. *Note:* the ionic peak at 555.1166 corresponds to [Cp*Mo(1,2-Ph_2_PC_6_H_4_S−CH_2_CH_3_)]^+^ resulting from **4** with the loss of a pyridyl fragment and two hydride ligands, while the peak at 323.1015 corresponds to the phosphine moiety Ph_2_PC_6_H_4_SCH_2_CH_3_.

*Result:* calcd for C_35_H_41_MoNPS ([**4**]^+^): 636.1751; found, 636.1735.

(a)
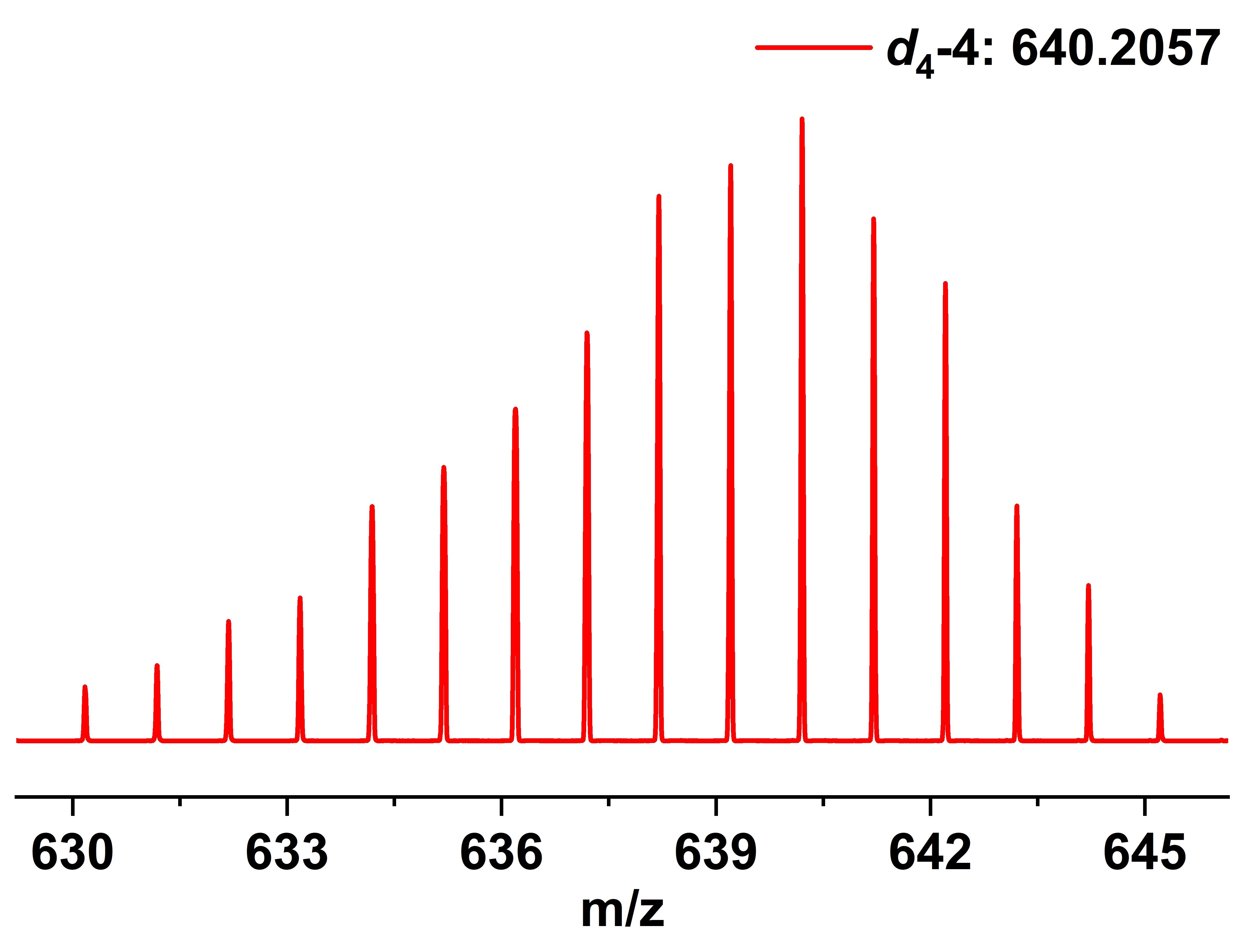
 (b)
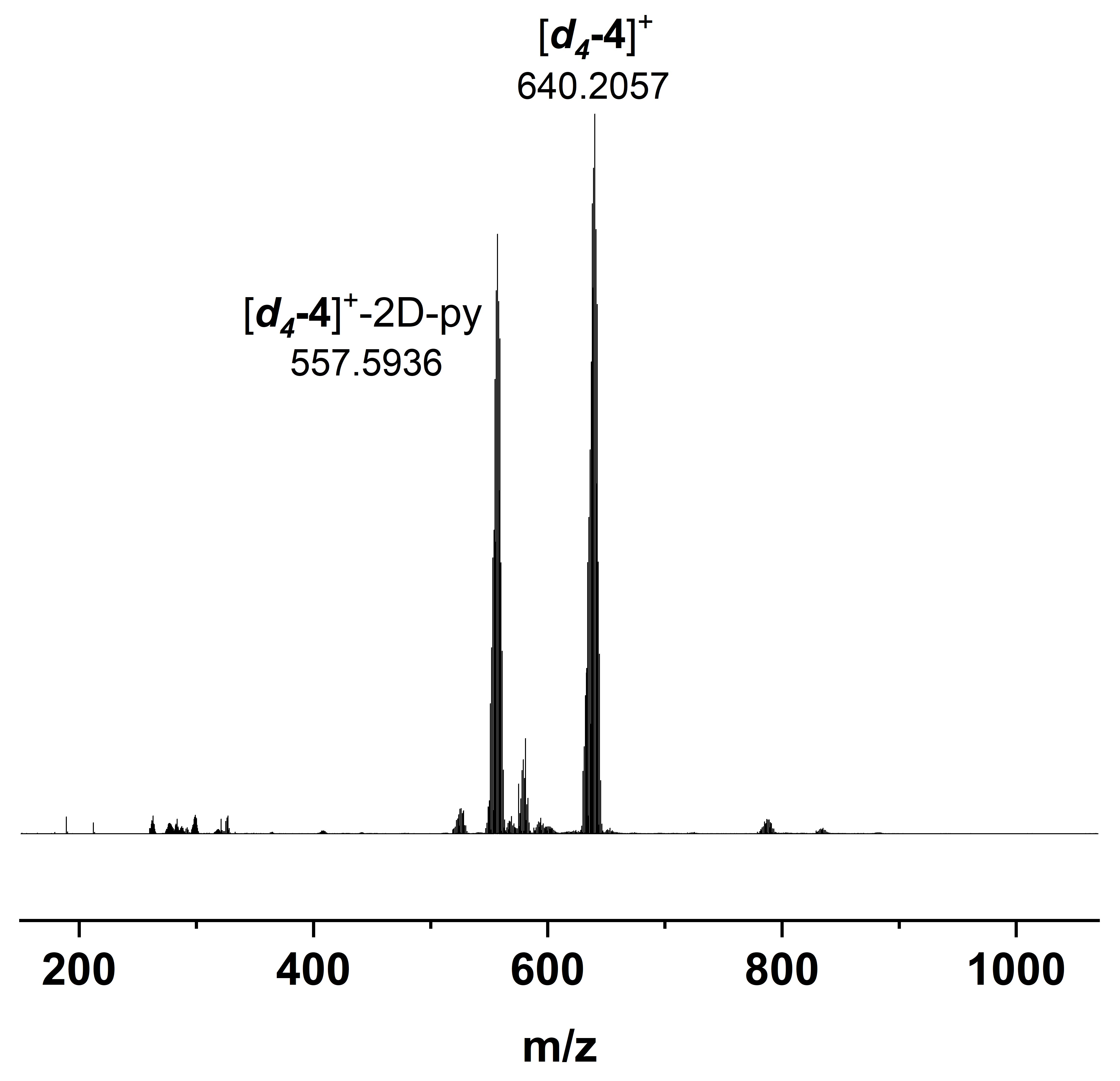


**Supplementary Figure 15.** HRMS spectrum of ***d*_4_-4**. (a) the zoom in mass spectrum of ***d*_4_-4**. (b) the whole mass spectrum of ***d*_4_-4**. *Note:* the ionic peak at 557.5936 corresponds to [Cp*Mo(1,2-Ph_2_PC_6_H_4_S−CHDCH_2_D)]^+^ resulting from **4** with the loss of a pyridyl fragment and two deuterium ligands.

*Result:* calcd for C_35_H_37_D_4_MoNPS ([***d*_4_-4**]^+^): 640.2057.

### IR Spectrum





**Supplementary Figure 16.** IR spectrum of PA.

*Result:* ν_C-H_: 2921 cm^−1^, 1373 cm^−1^, 1016 cm^−1^, 734 cm^−1^; ν_C=C_: 1651 cm^−1^, 1461 cm^−1^.

### NMR Spectra


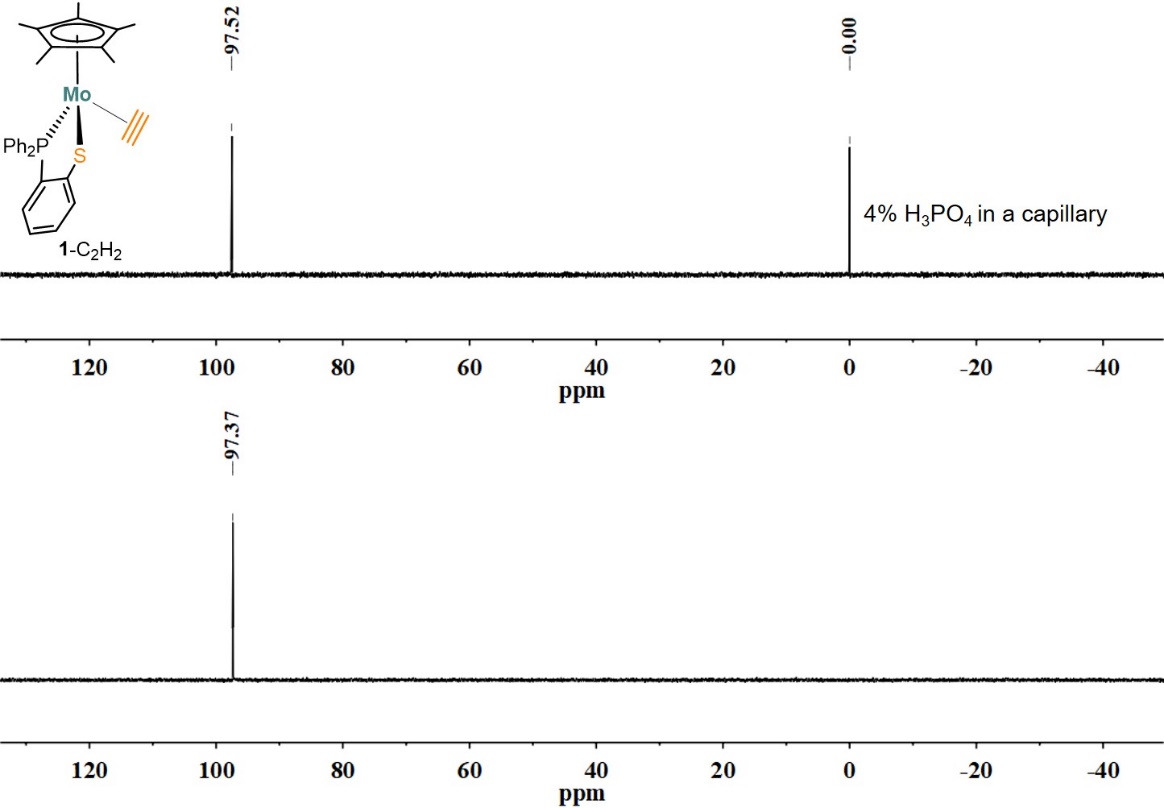


**Supplementary Figure 17.** ^31^P{^1^H} NMR (202 MHz, *d_8_*-THF) spectrum of **1**-C_2_H_2_ with and without 4% H_3_PO_4_ used as internal standard in the capillary at 273 K.


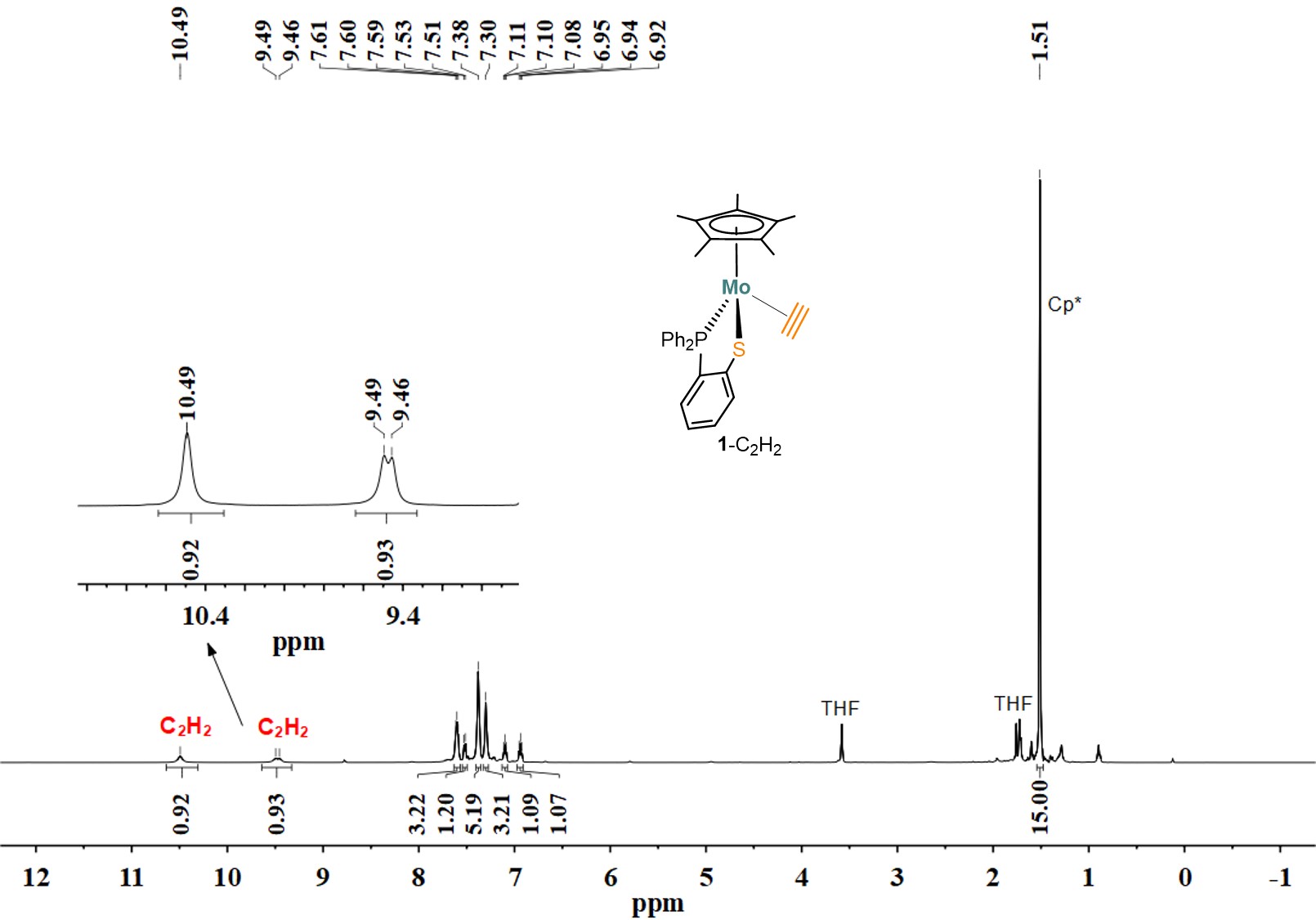


**Supplementary Figure 18.** ^1^H NMR (202 MHz, *d_8_*-THF) spectrum of **1**-C_2_H_2_ at 273 K.


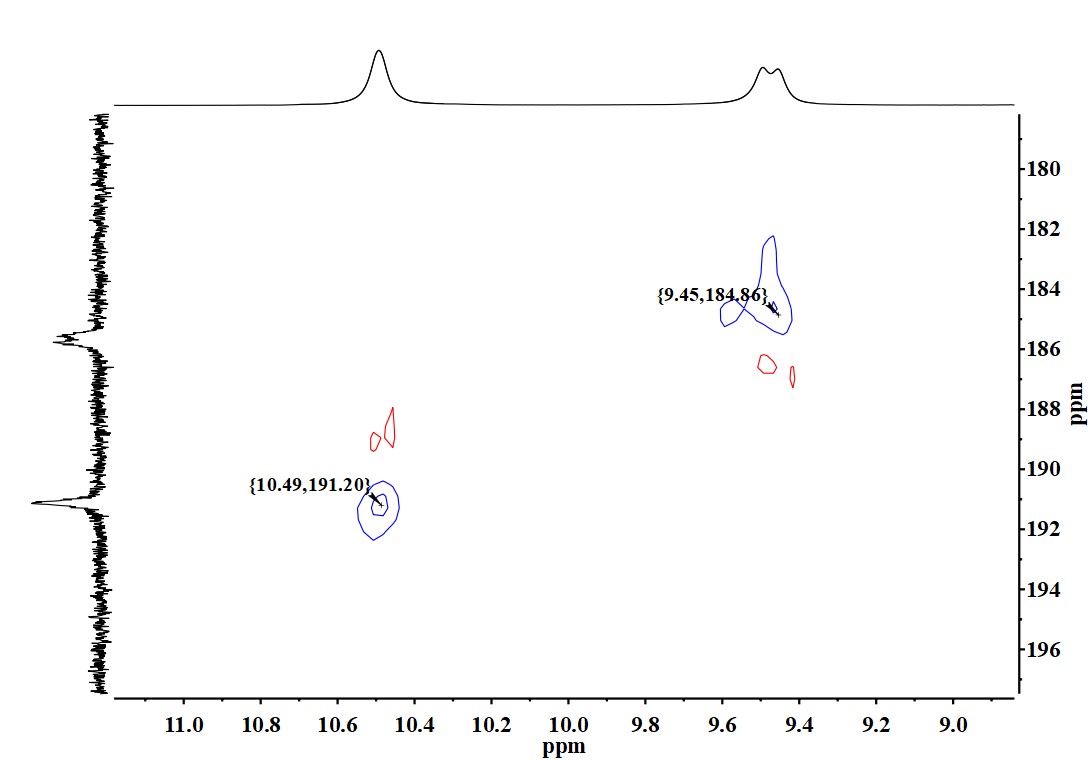


**Supplementary Figure 19.** ^1^H, ^13^C{^1^H}-HSQC spectrum of **1**-C_2_H_2_ in *d_8_*-THF at 273 K.


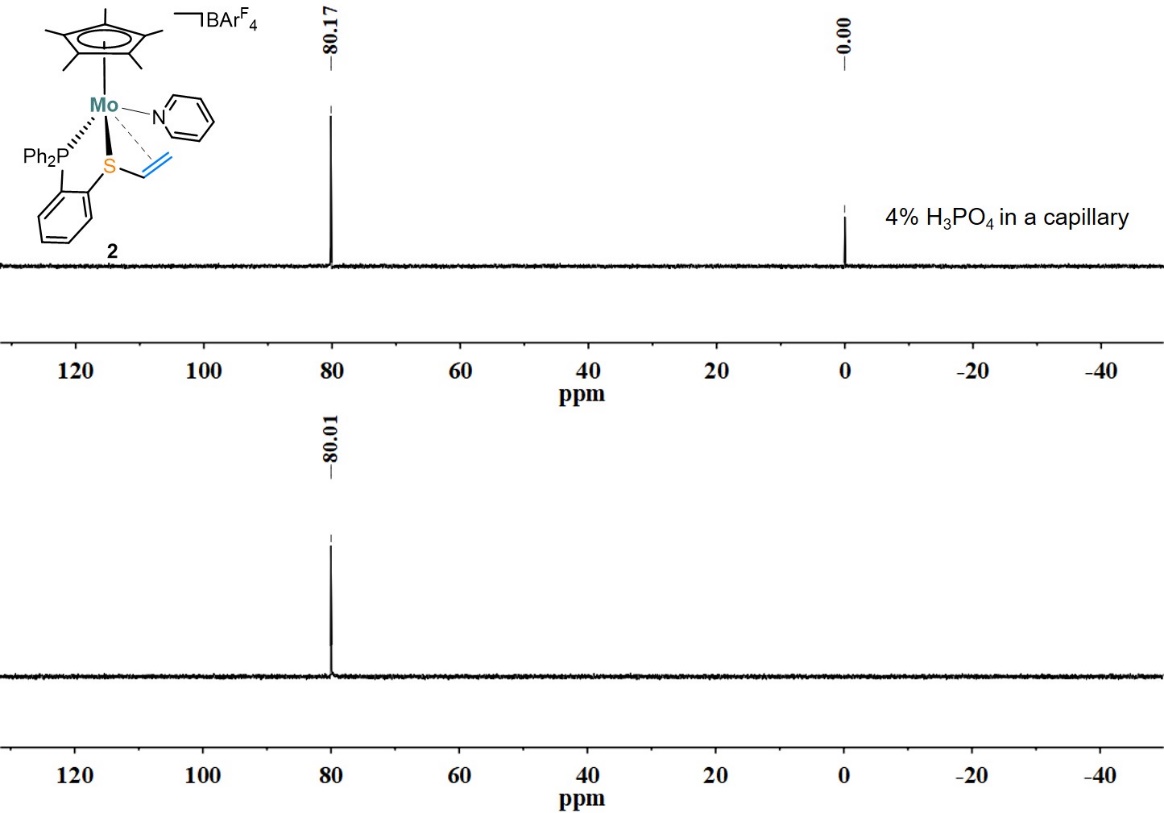


**Supplementary Figure 20.** ^31^P{^1^H} NMR (202 MHz, *d_8_*-THF) spectrum of **2** with and without 4% H_3_PO_4_ used as internal standard in the capillary.


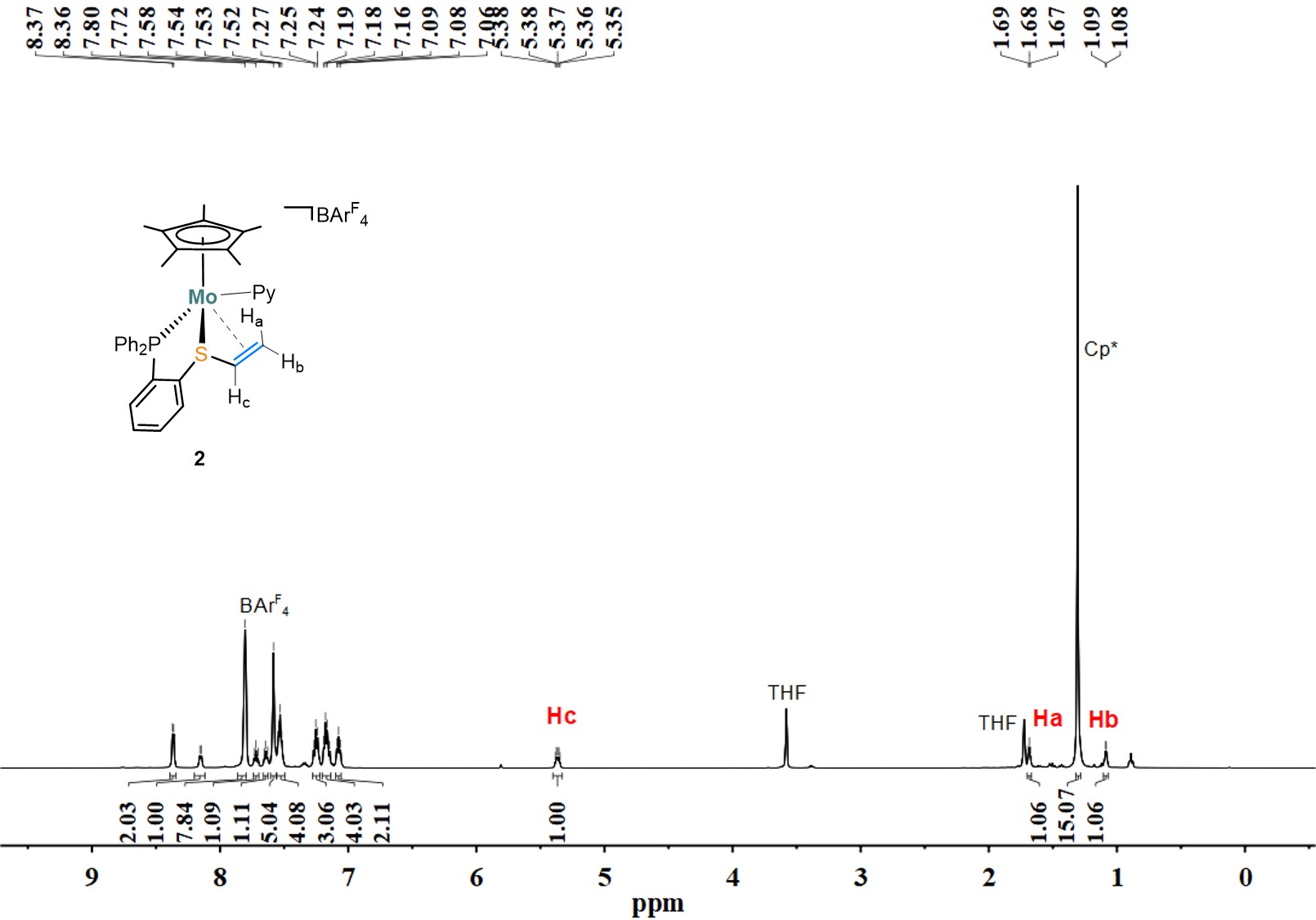


**Supplementary Figure 21.** ^1^H NMR (202 MHz, *d_8_*-THF) spectrum of **2**.


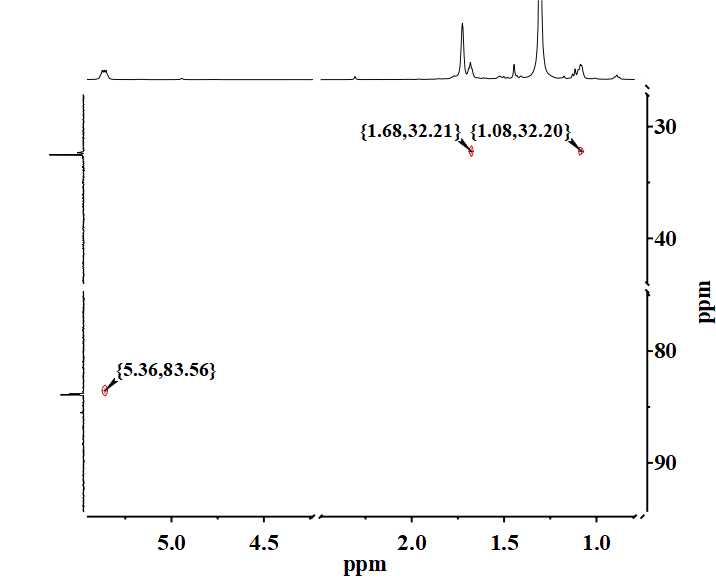


**Supplementary Figure 22.** ^1^H, ^13^C{^1^H}-HSQC spectrum of **2** in *d_8_*-THF.


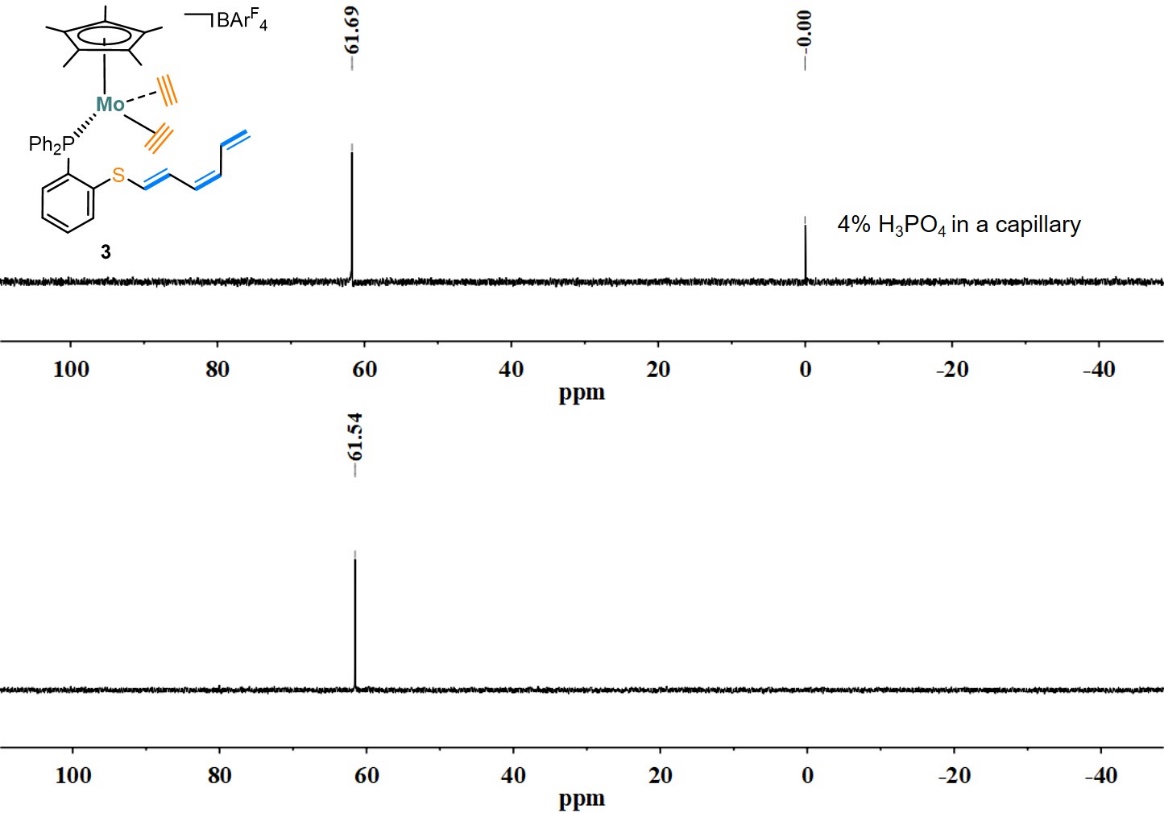


**Supplementary Figure 23.** ^31^P{^1^H} NMR (202 MHz, *d_8_*-THF) spectrum of **3** with and without 4% H_3_PO_4_ used as internal standard in the capillary.


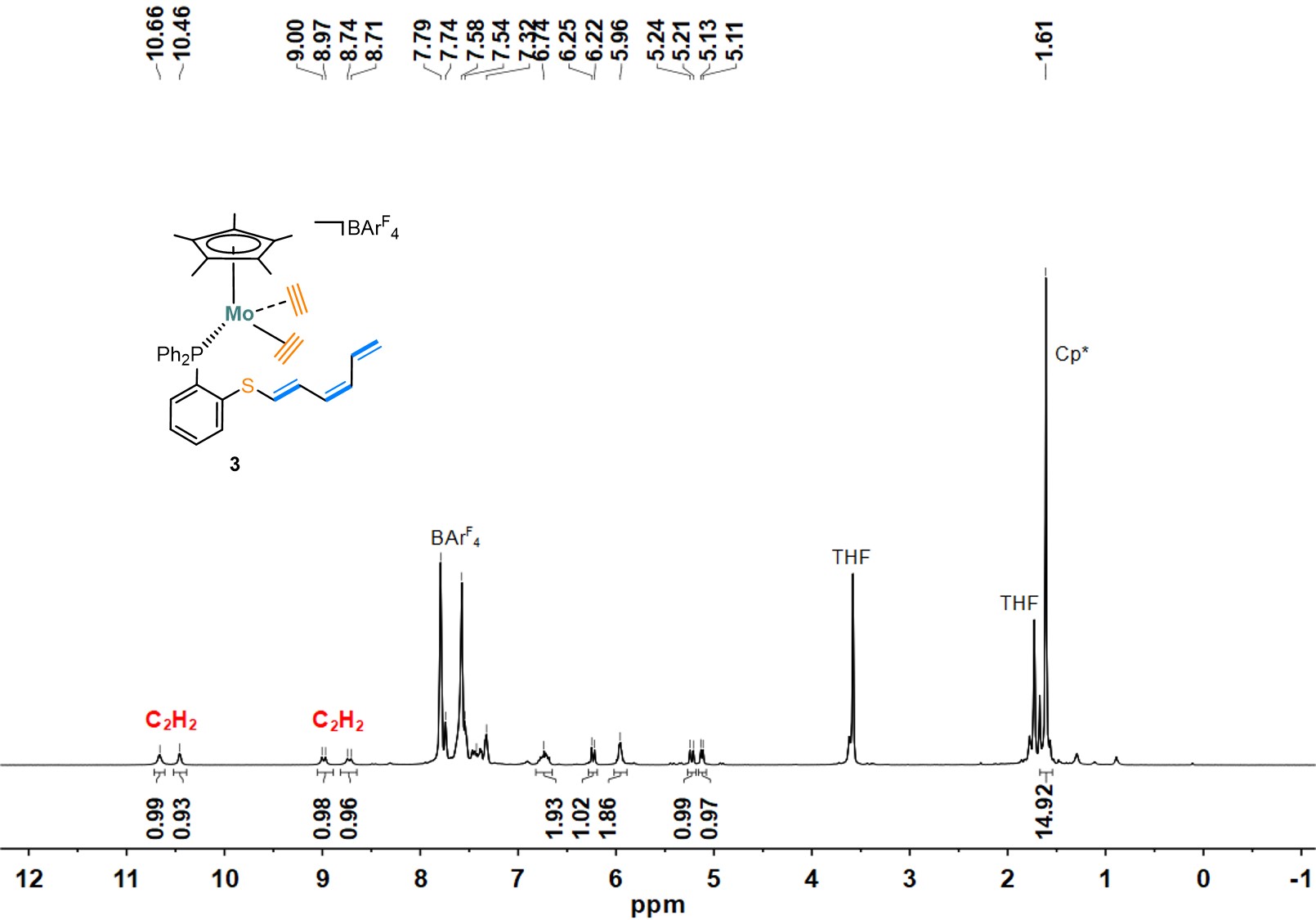


**Supplementary Figure 24.** ^1^H NMR (202 MHz, *d_8_*-THF) spectrum of **3**.


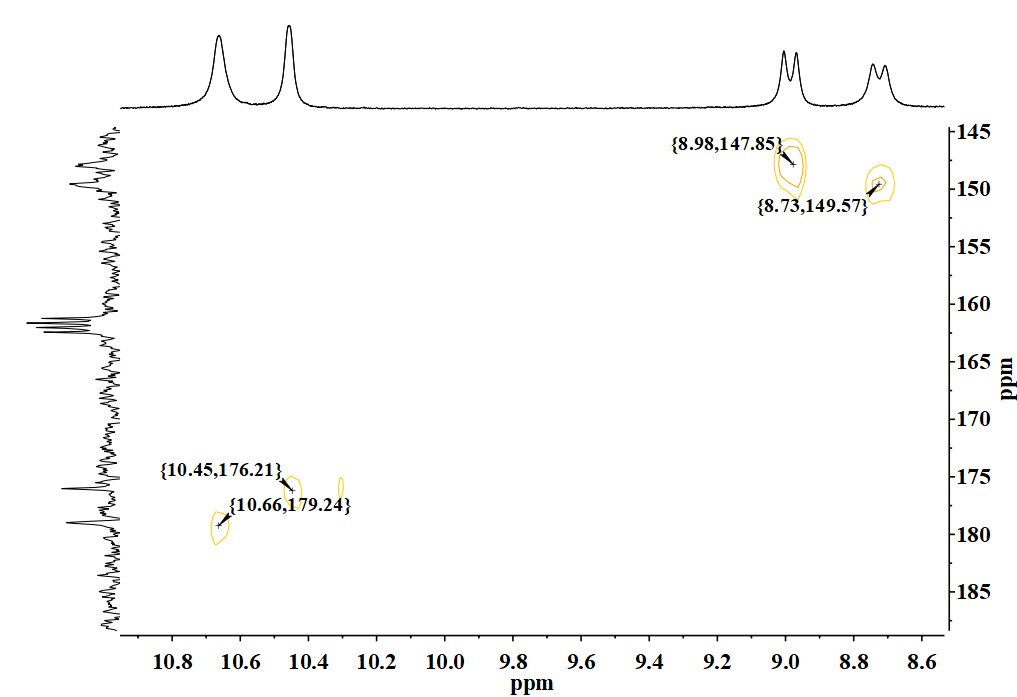


**Supplementary Figure 25.** ^1^H, ^13^C{^1^H}-HSQC spectrum of **3** in *d_8_*-THF.


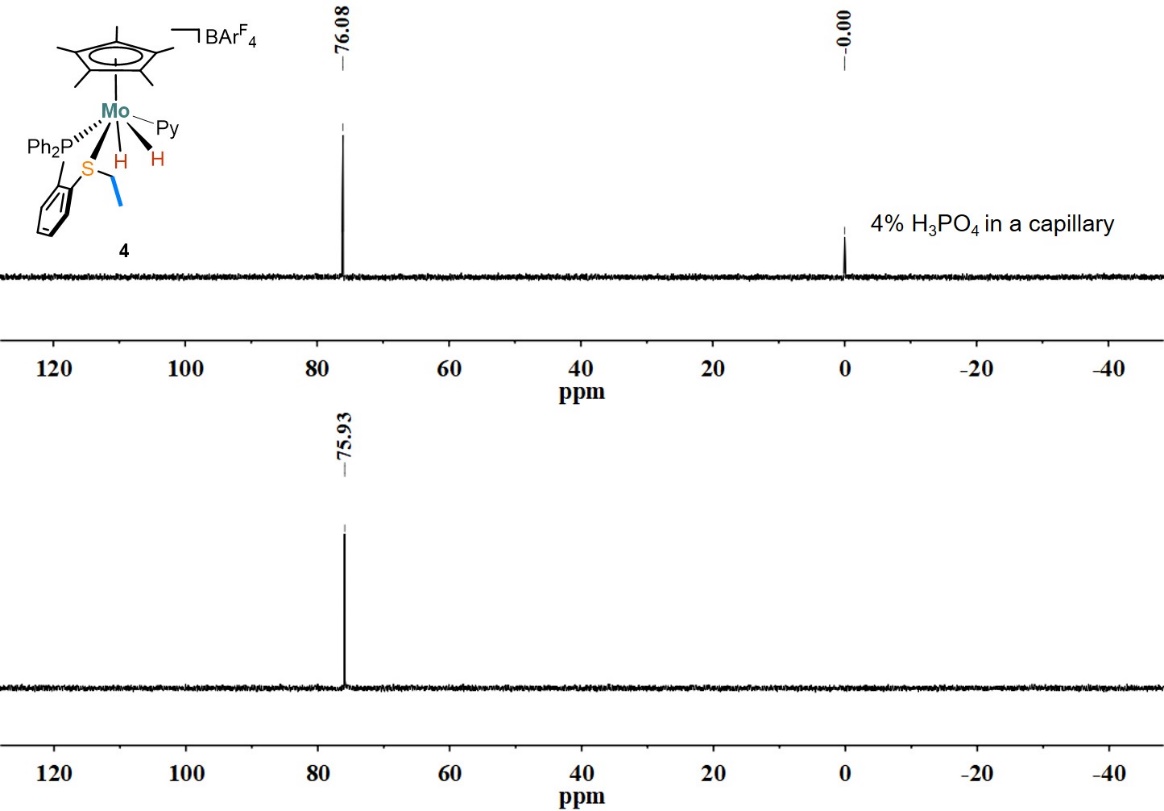


**Supplementary Figure 26.** ^31^P{^1^H} NMR (202 MHz, *d_8_*-THF) spectrum of **4** with and without 4% H_3_PO_4_ used as internal standard in the capillary at 253 K.


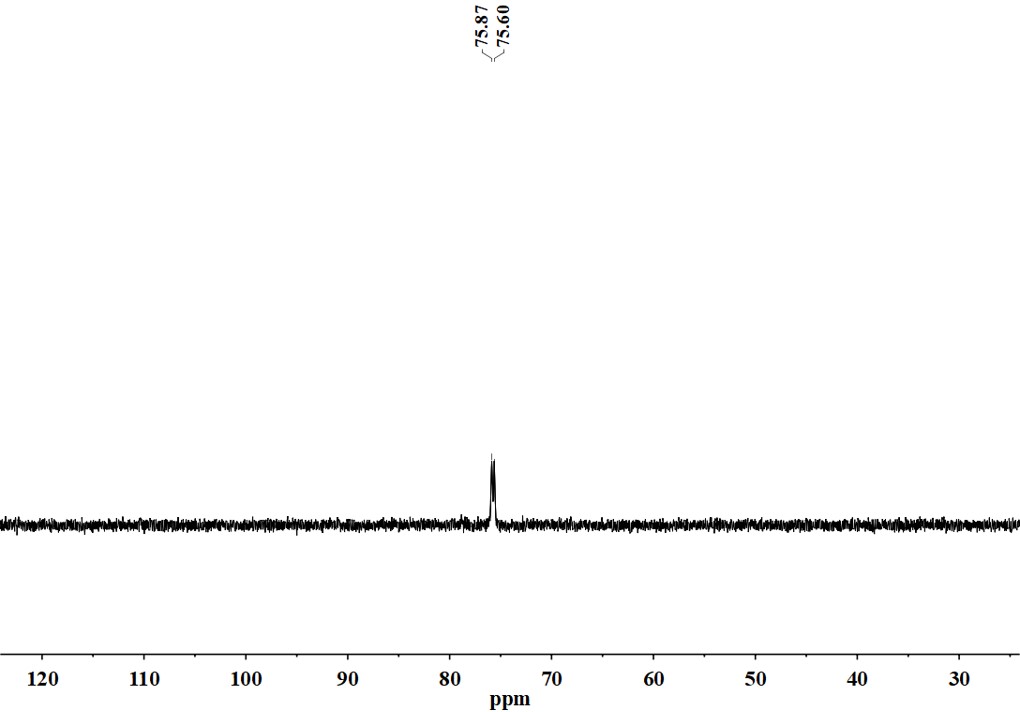


**Supplementary Figure 27.** ^31^P NMR (202 MHz, C_6_D_5_Cl) spectrum of **4** at 253 K.


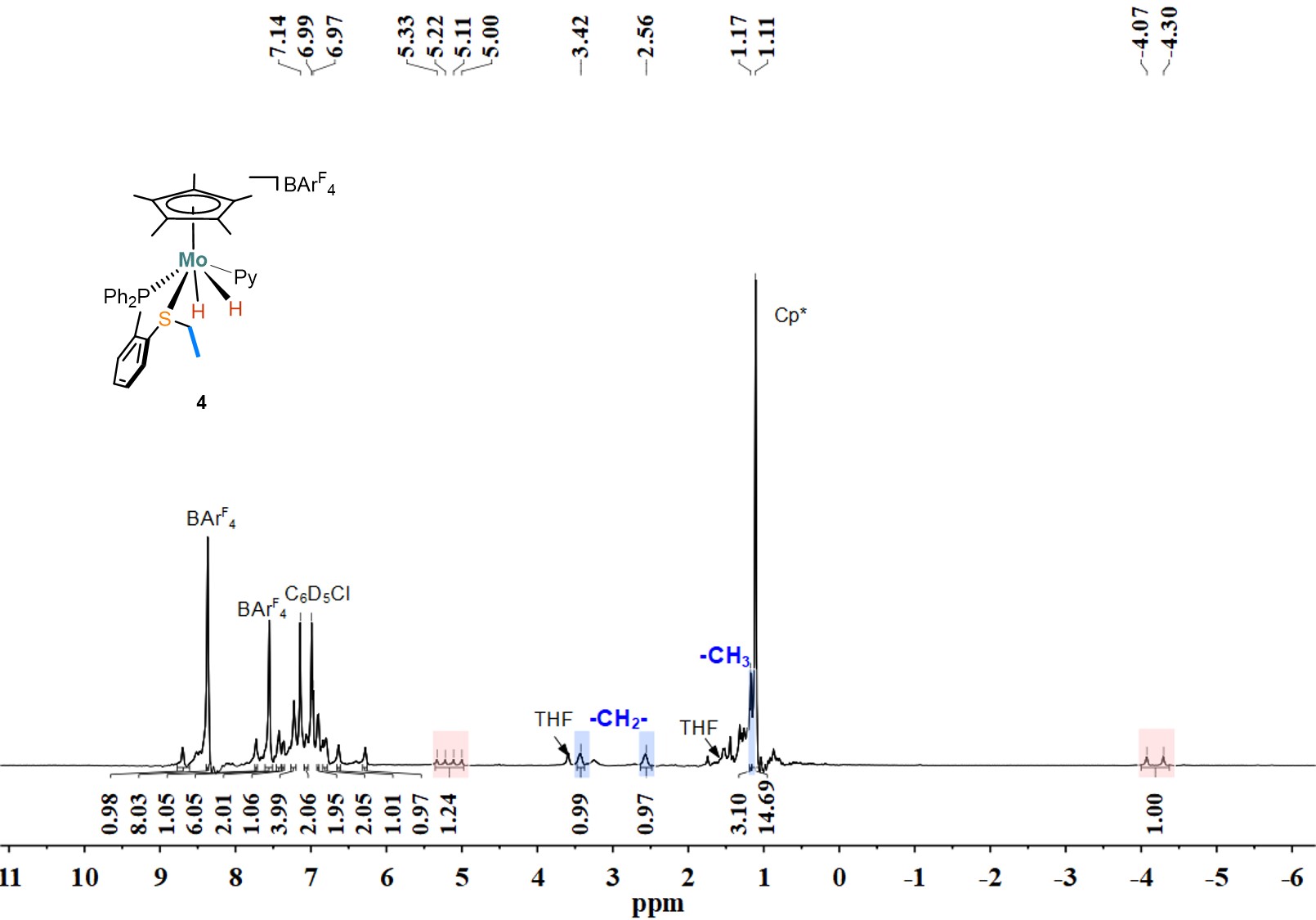


**Supplementary Figure 28.** ^1^H NMR (202 MHz, C_6_D_5_Cl) spectrum of **4** at 253 K.


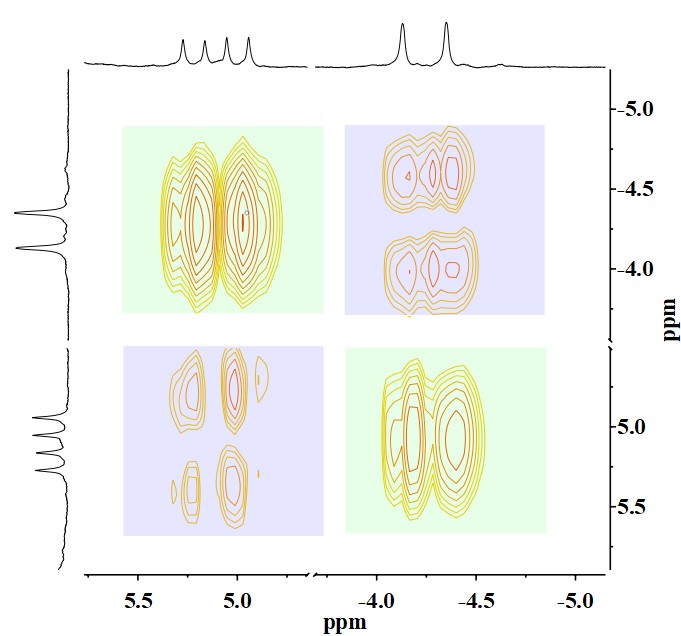


**Supplementary Figure 29.** ^1^H, ^1^H -COSY spectrum of **4** in C_6_D_5_Cl at 253 K.

**^1^H NMR Spectra of the Catalytic Reaction Mixtures**


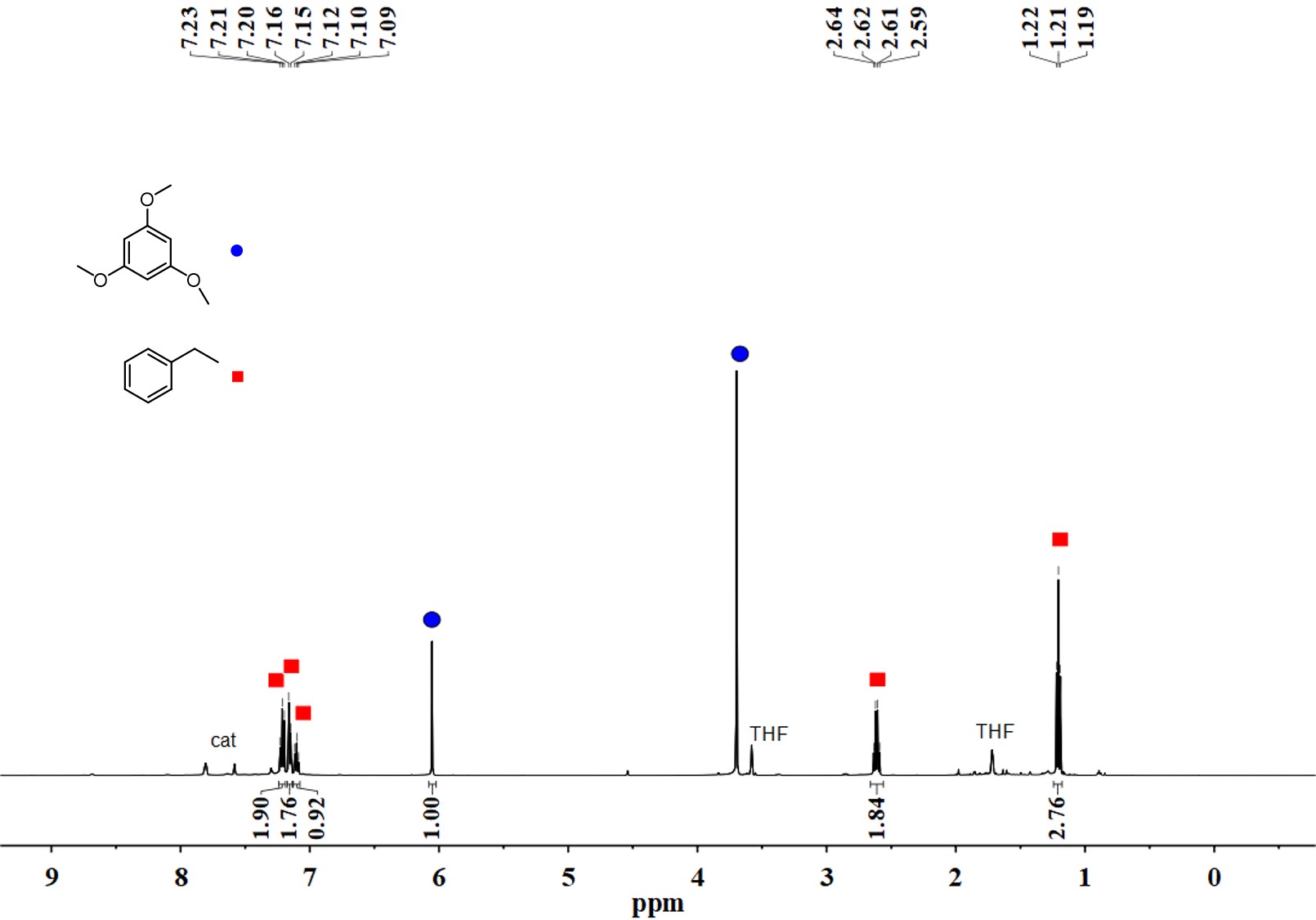


**Supplementary Figure 30.** ^1^H NMR (500 MHz, *d_8_*-THF) spectrum recorded for the catalytic reaction of **5a**.


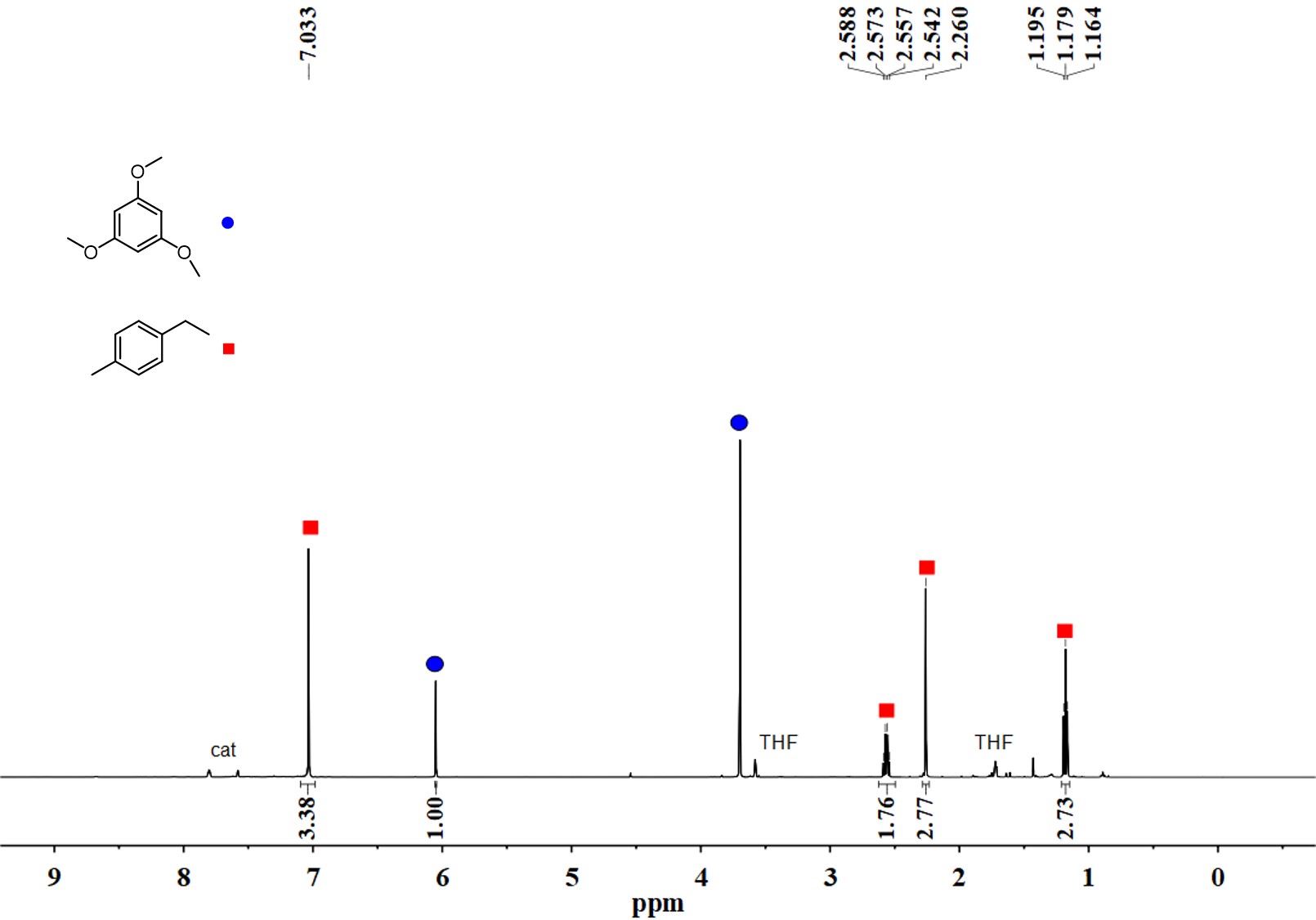


**Supplementary Figure 31.** ^1^H NMR (500 MHz, *d_8_*-THF) spectrum recorded for the catalytic reaction of **5b**.


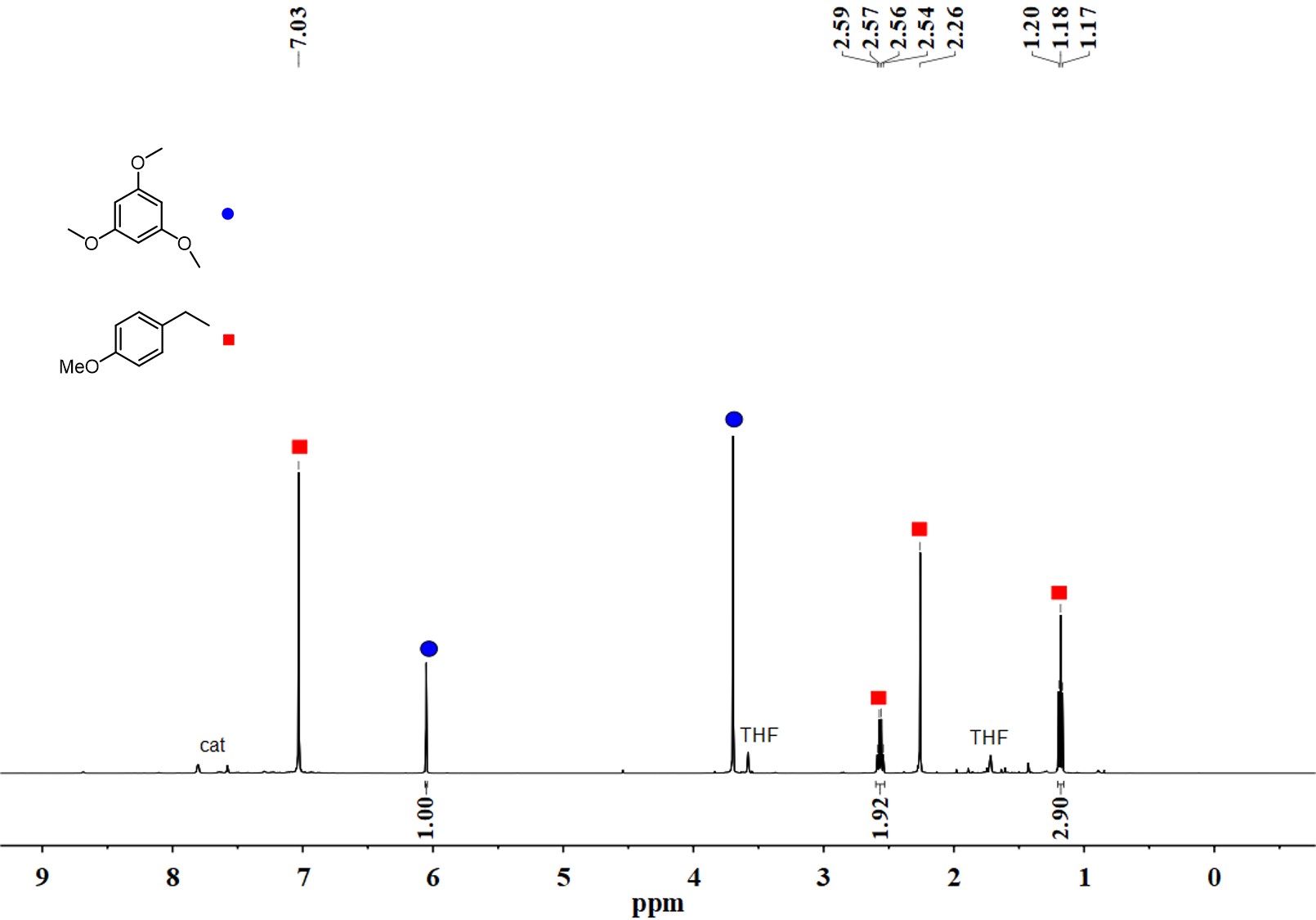


**Supplementary Figure 32.** ^1^H NMR (500 MHz, *d_8_*-THF) spectrum recorded for the catalytic reaction of **5c**.


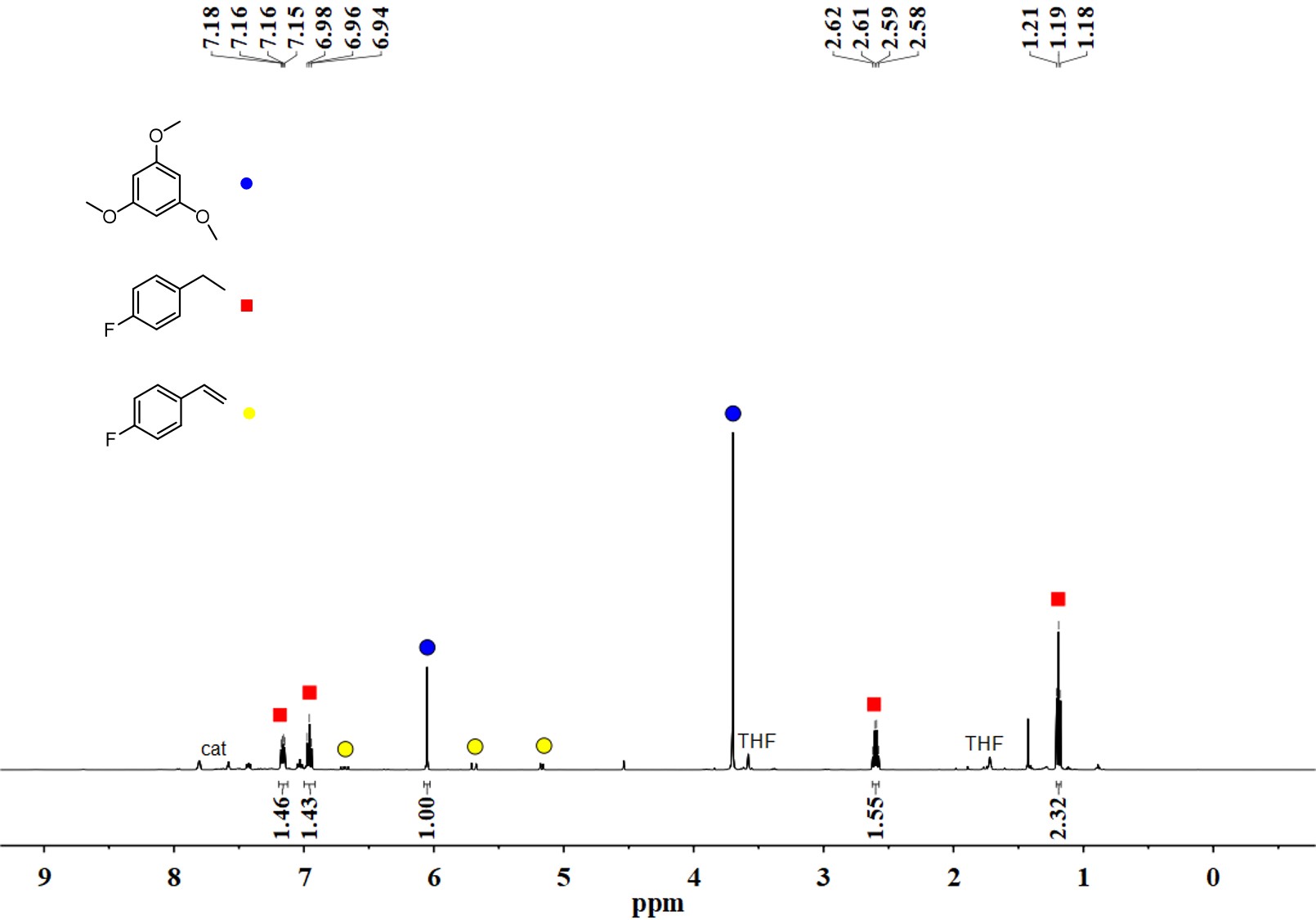


**Supplementary Figure 33.** ^1^H NMR (500 MHz, *d*_8_-THF) spectrum recorded for the catalytic reaction of **5d**.


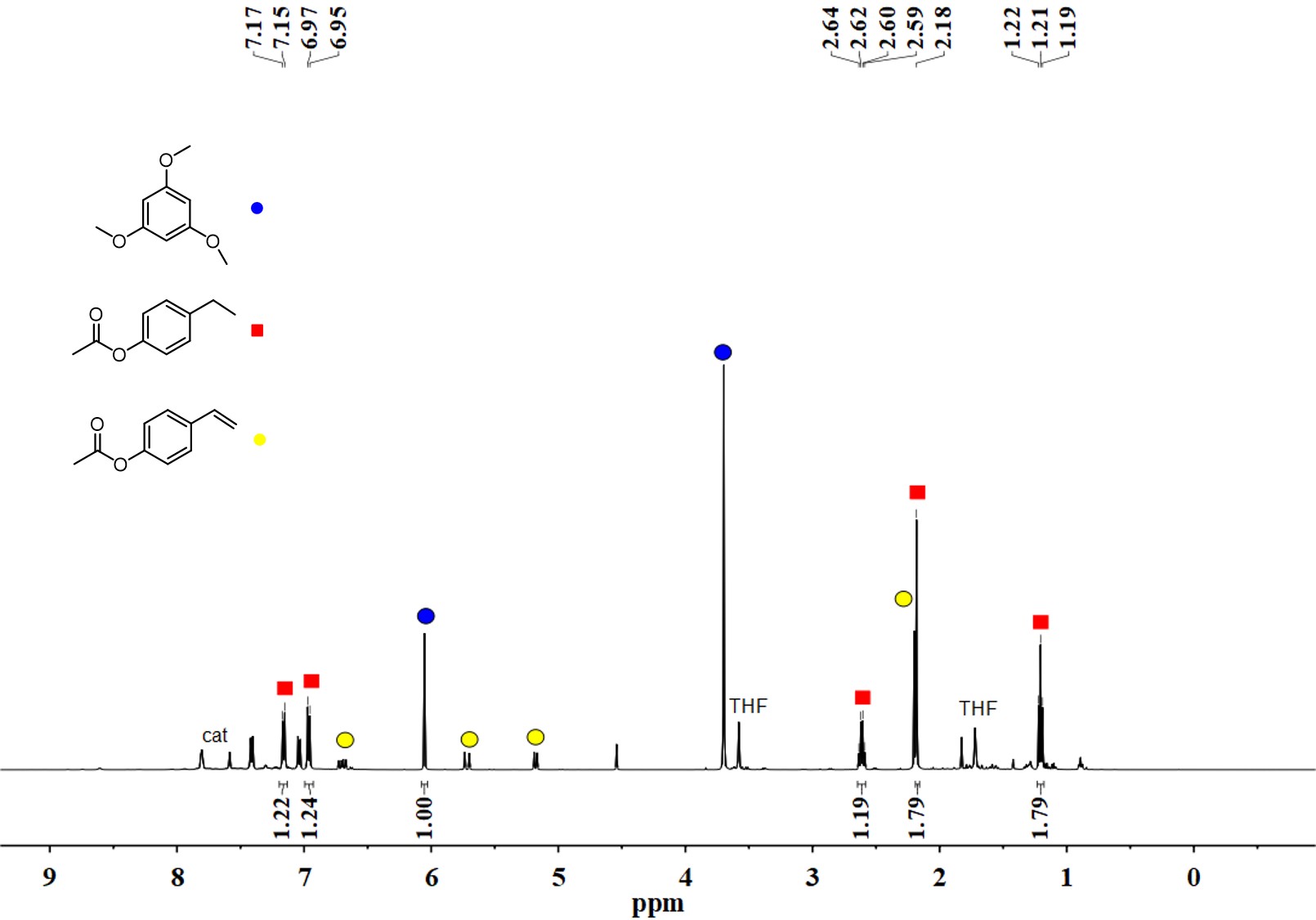


**Supplementary Figure 34.** ^1^H NMR (500 MHz, *d*_8_-THF) spectrum recorded for the catalytic reaction of **5e**.


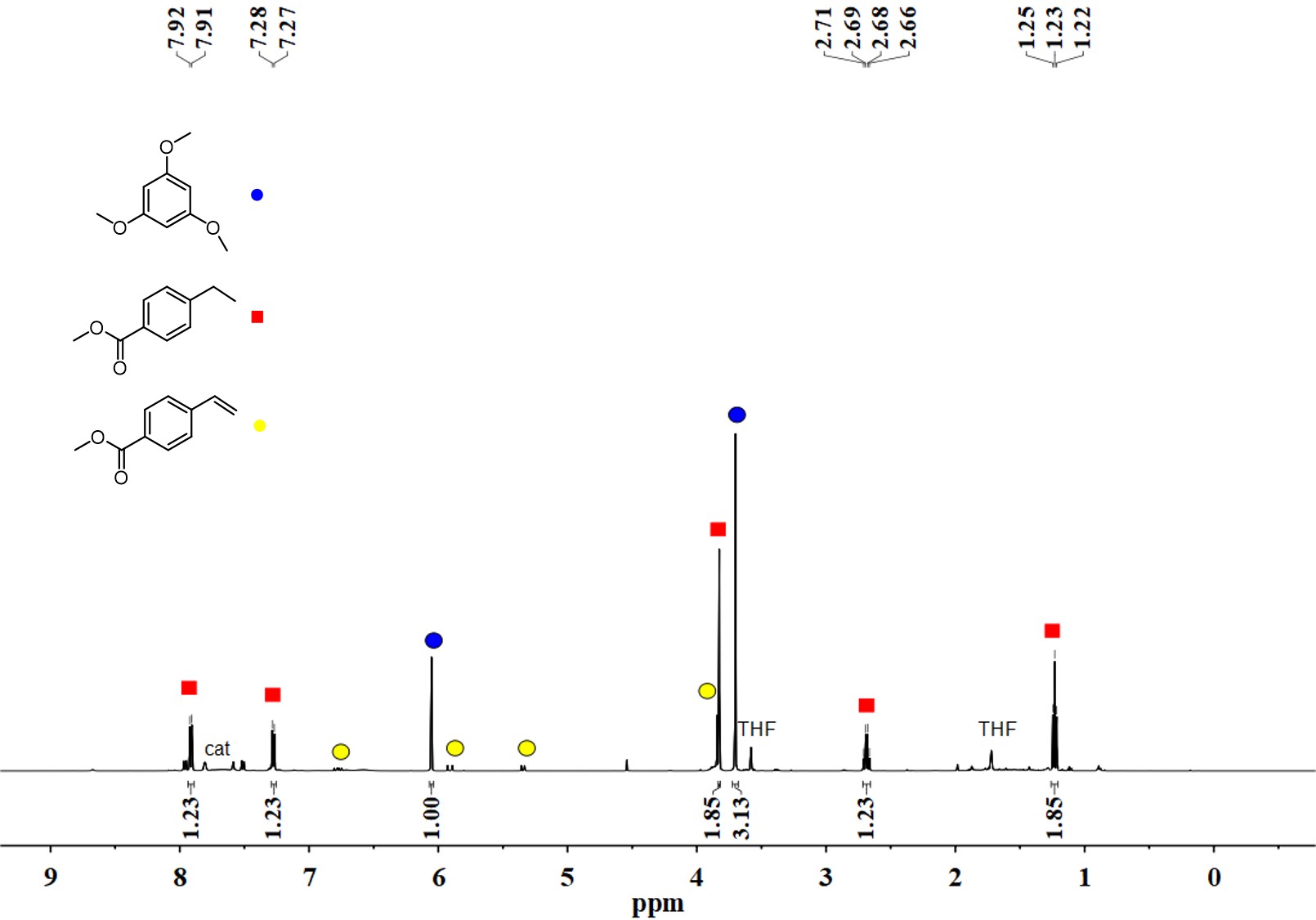


**Supplementary Figure 35.** ^1^H NMR (500 MHz, *d*_8_-THF) spectrum recorded for the catalytic reaction of **5f**.


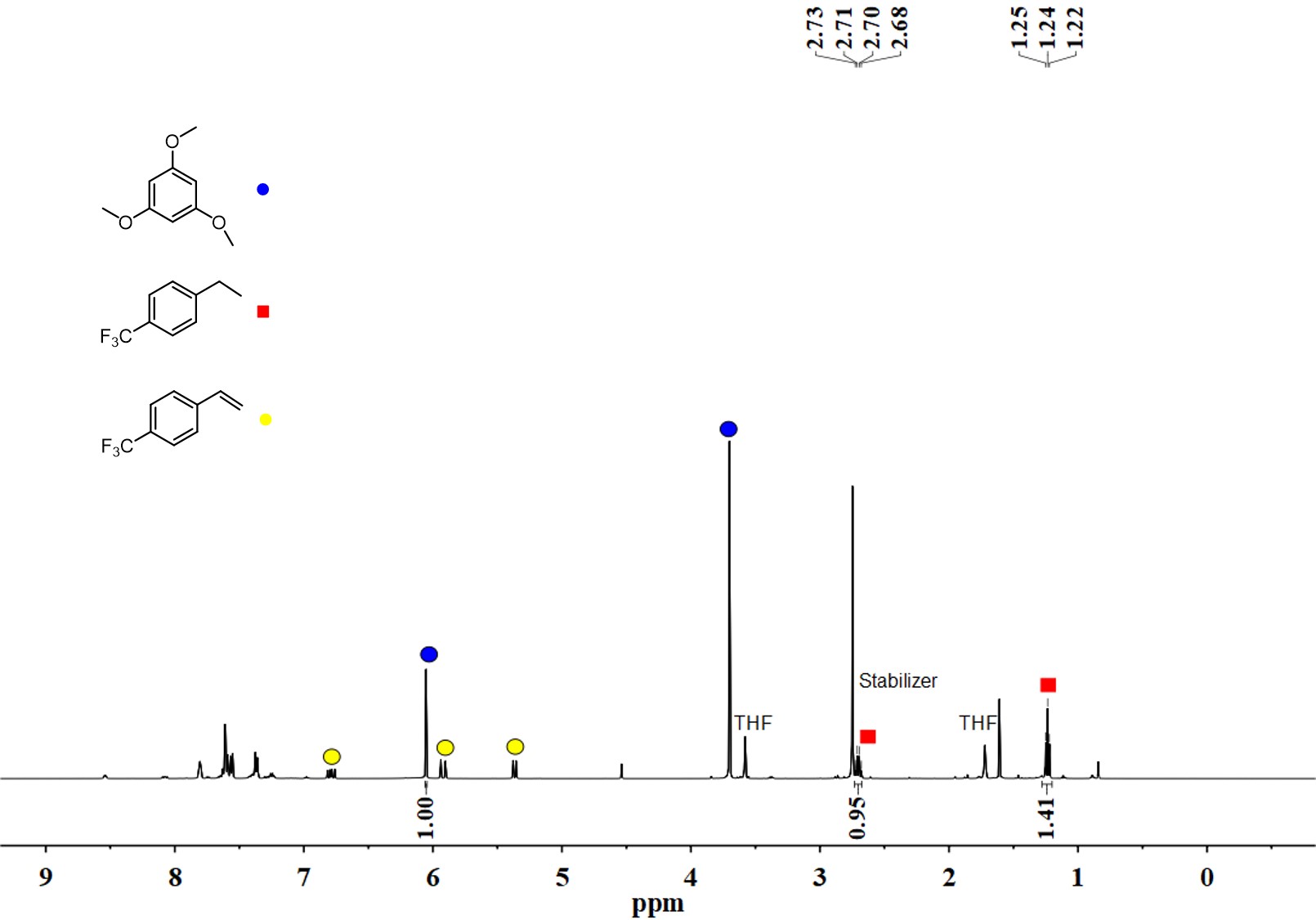


**Supplementary Figure 36.** ^1^H NMR (500 MHz, *d_8_*-THF) spectrum recorded for the catalytic reaction of **5g**.


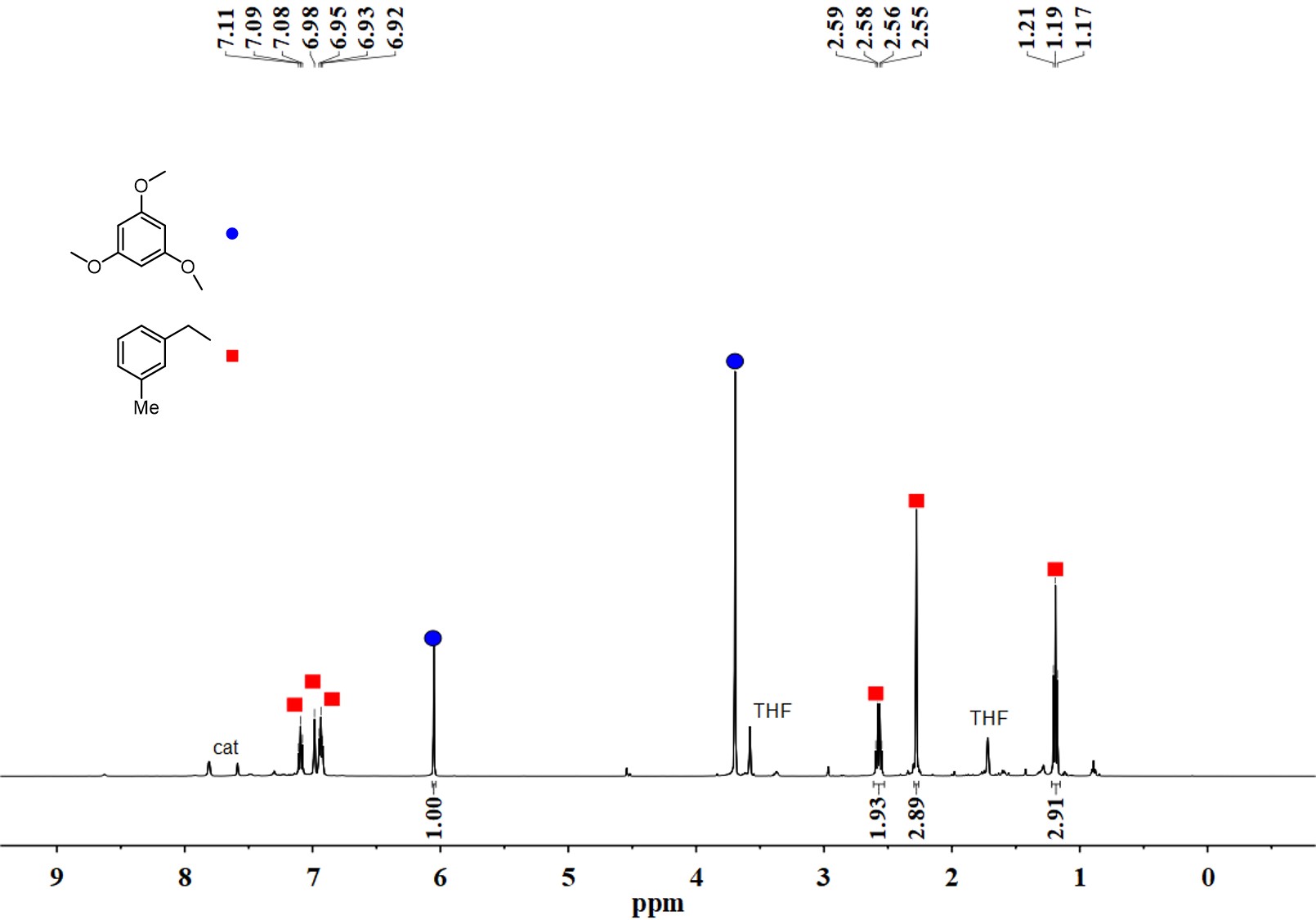


**Supplementary Figure 37.** ^1^H NMR (500 MHz, *d_8_*-THF) spectrum recorded for the catalytic reaction of **5h**.


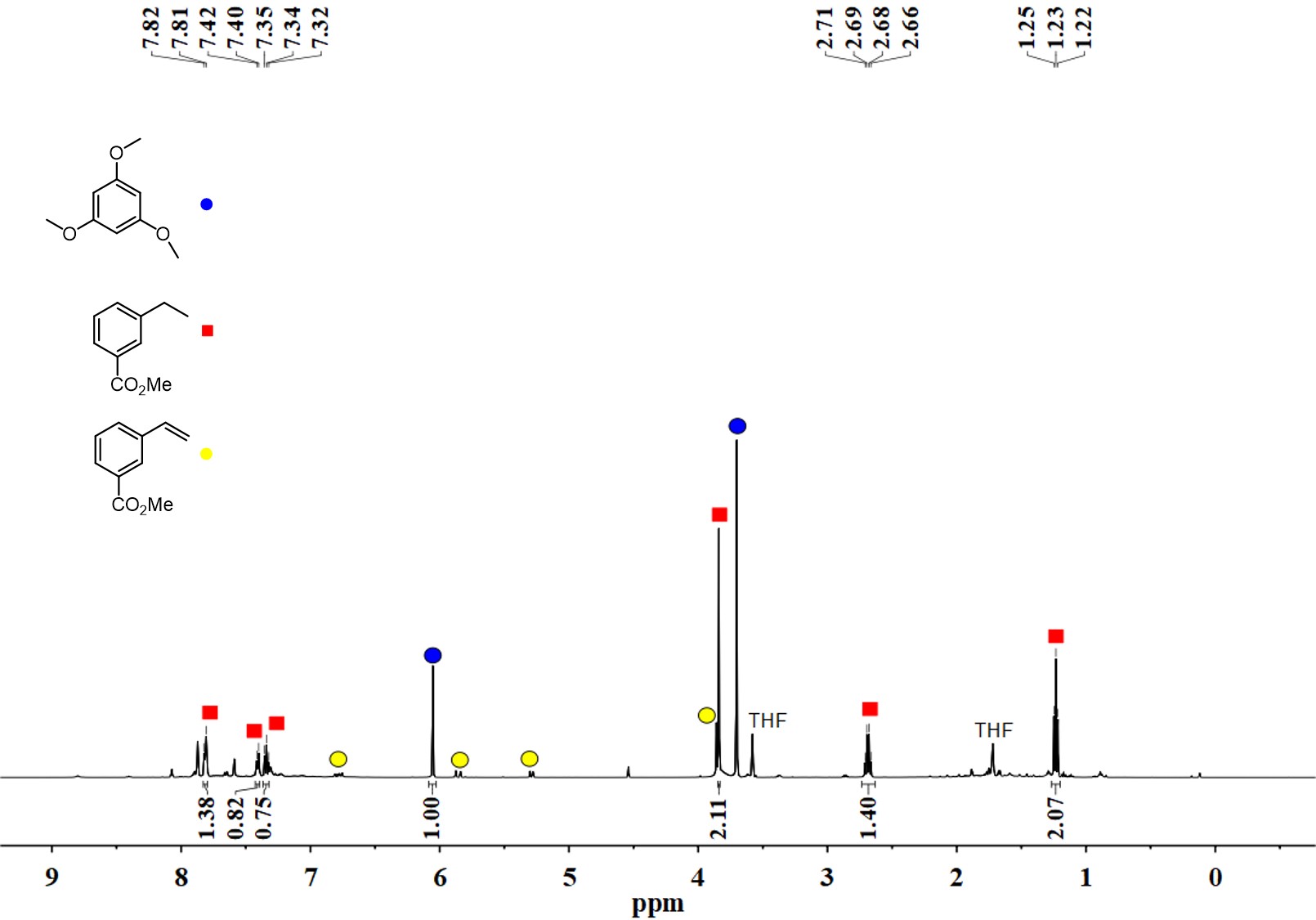


**Supplementary Figure 38.** ^1^H NMR (500 MHz, *d_8_*-THF) spectrum recorded for the catalytic reaction of **5i**.


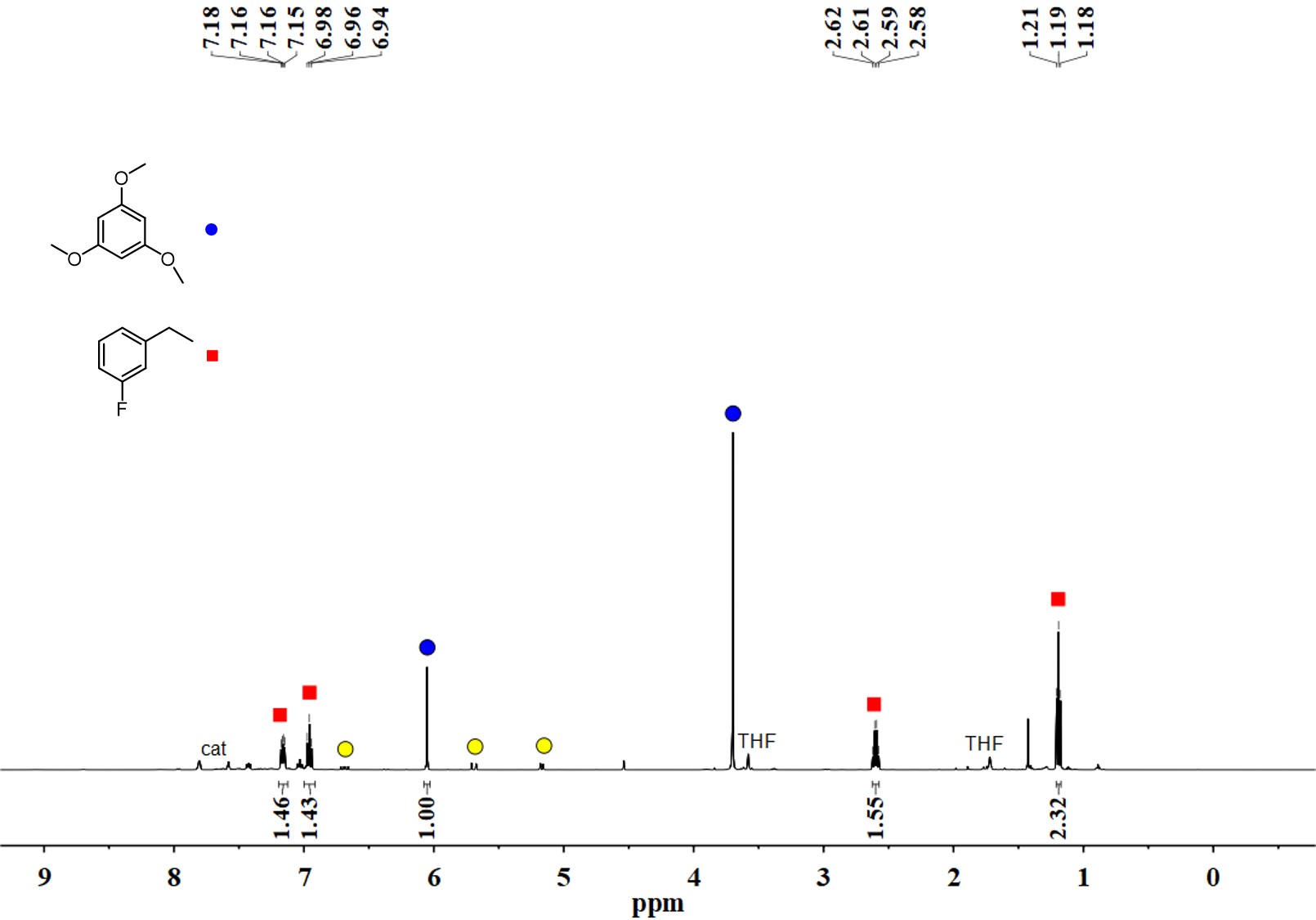


**Supplementary Figure 39.** ^1^H NMR (500 MHz, *d_8_*-THF) spectrum recorded for the catalytic reaction of **5j**.


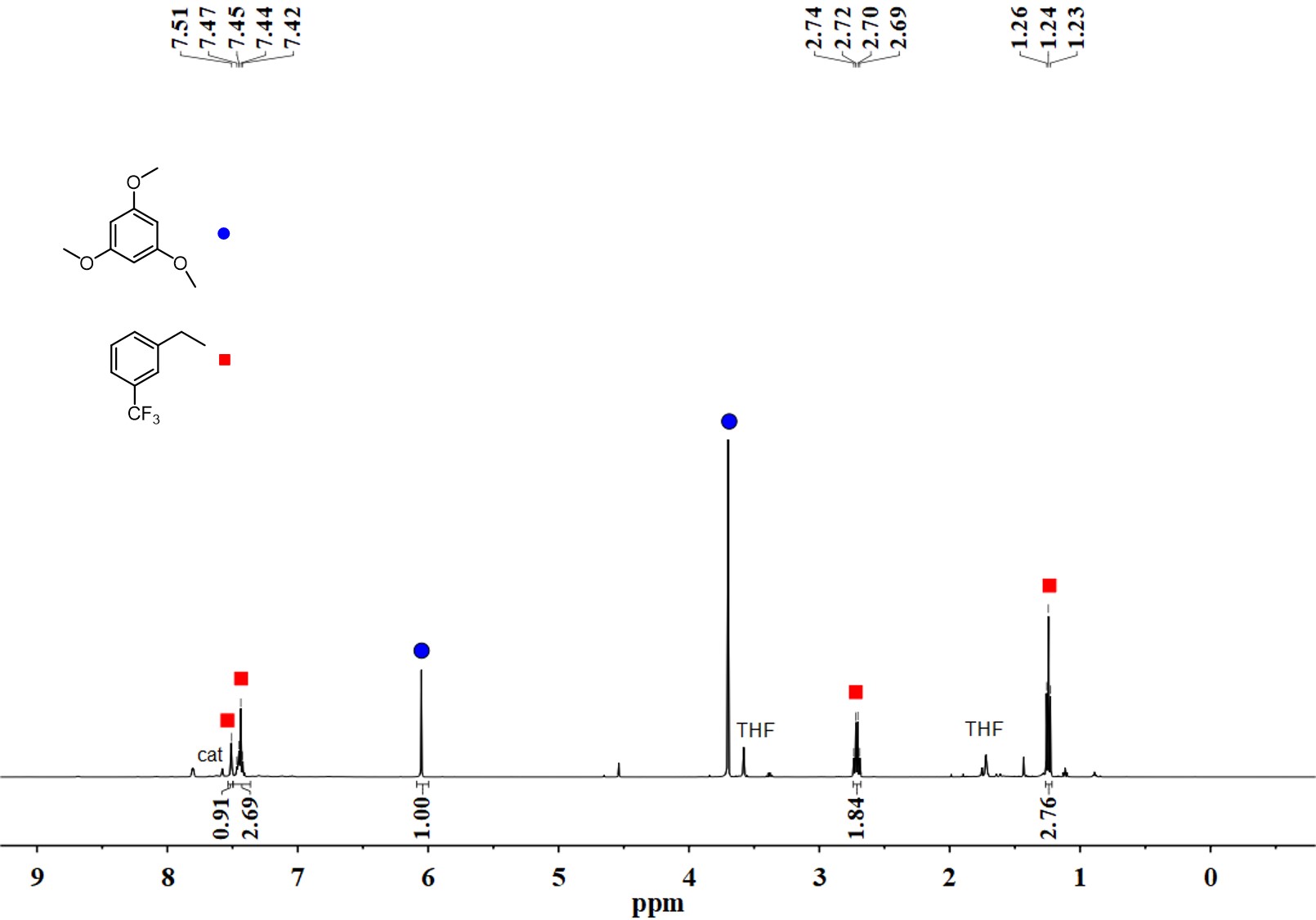


**Supplementary Figure 40.** ^1^H NMR (500 MHz, *d_8_*-THF) spectrum recorded for the catalytic reaction of **5k**.


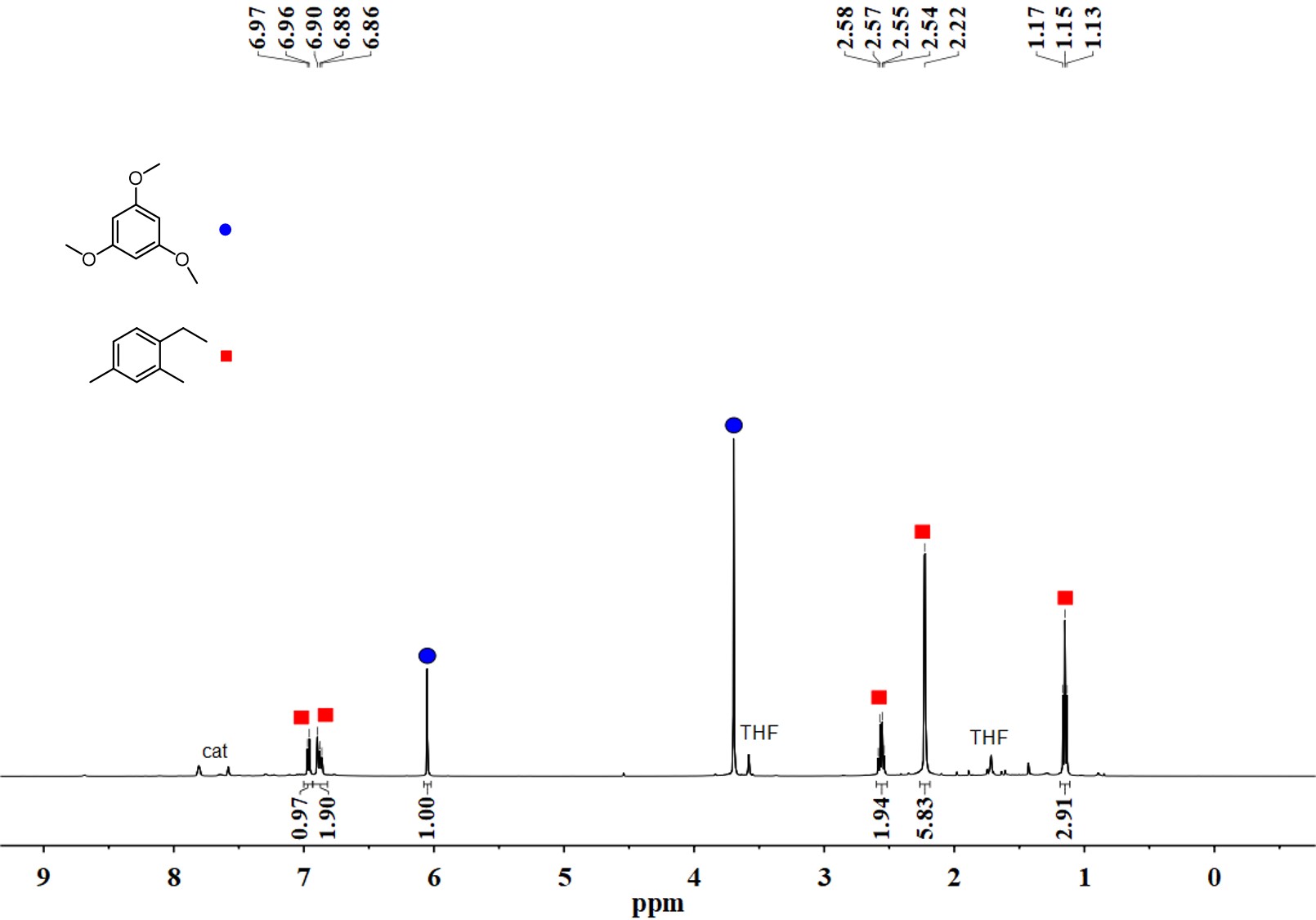


**Supplementary Figure 41.** ^1^H NMR (500 MHz, *d_8_*-THF) spectrum recorded for the catalytic reaction of **5l**.


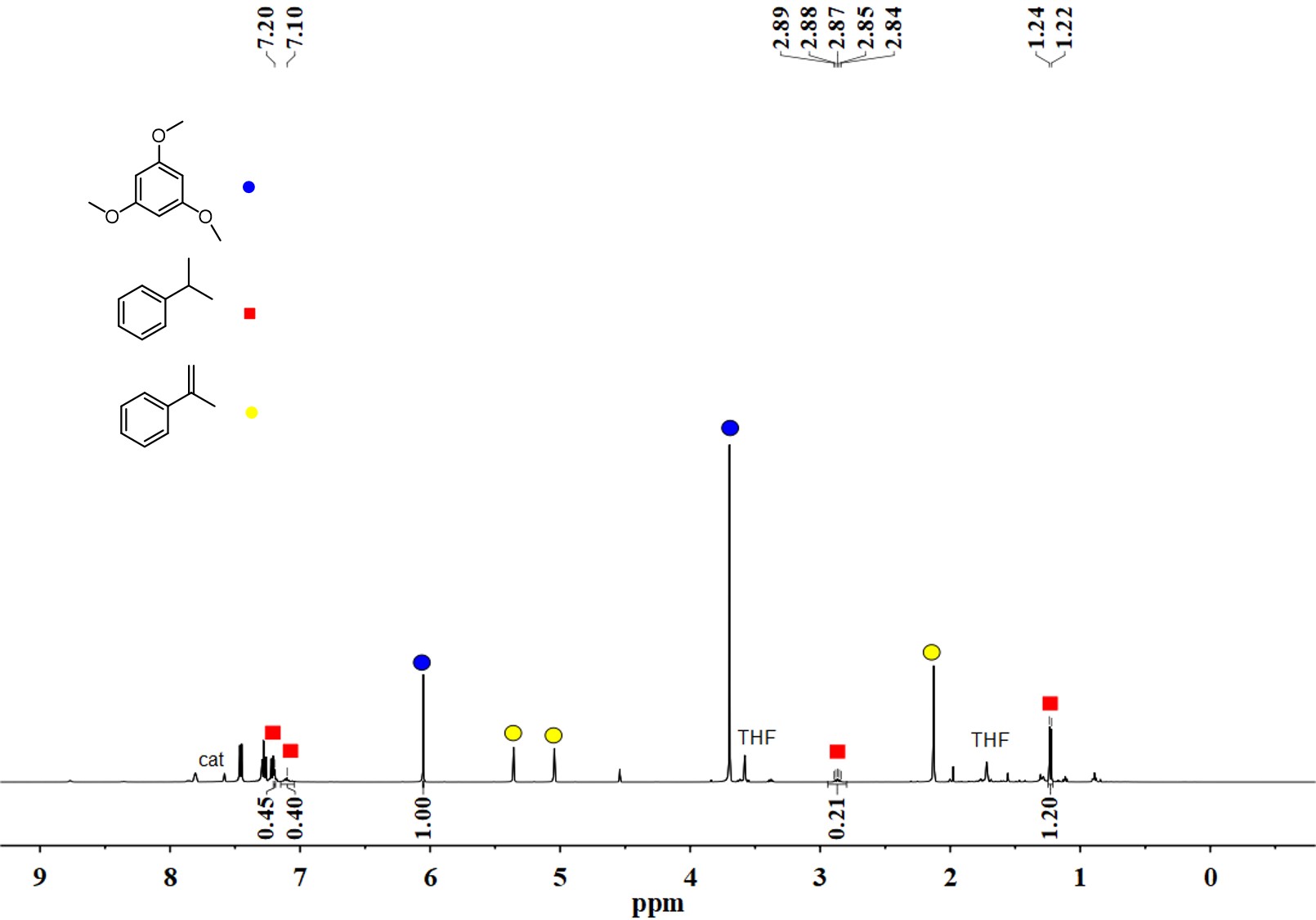


**Supplementary Figure 42.** ^1^H NMR (500 MHz, *d_8_*-THF) spectrum recorded for the catalytic reaction of **5m**.


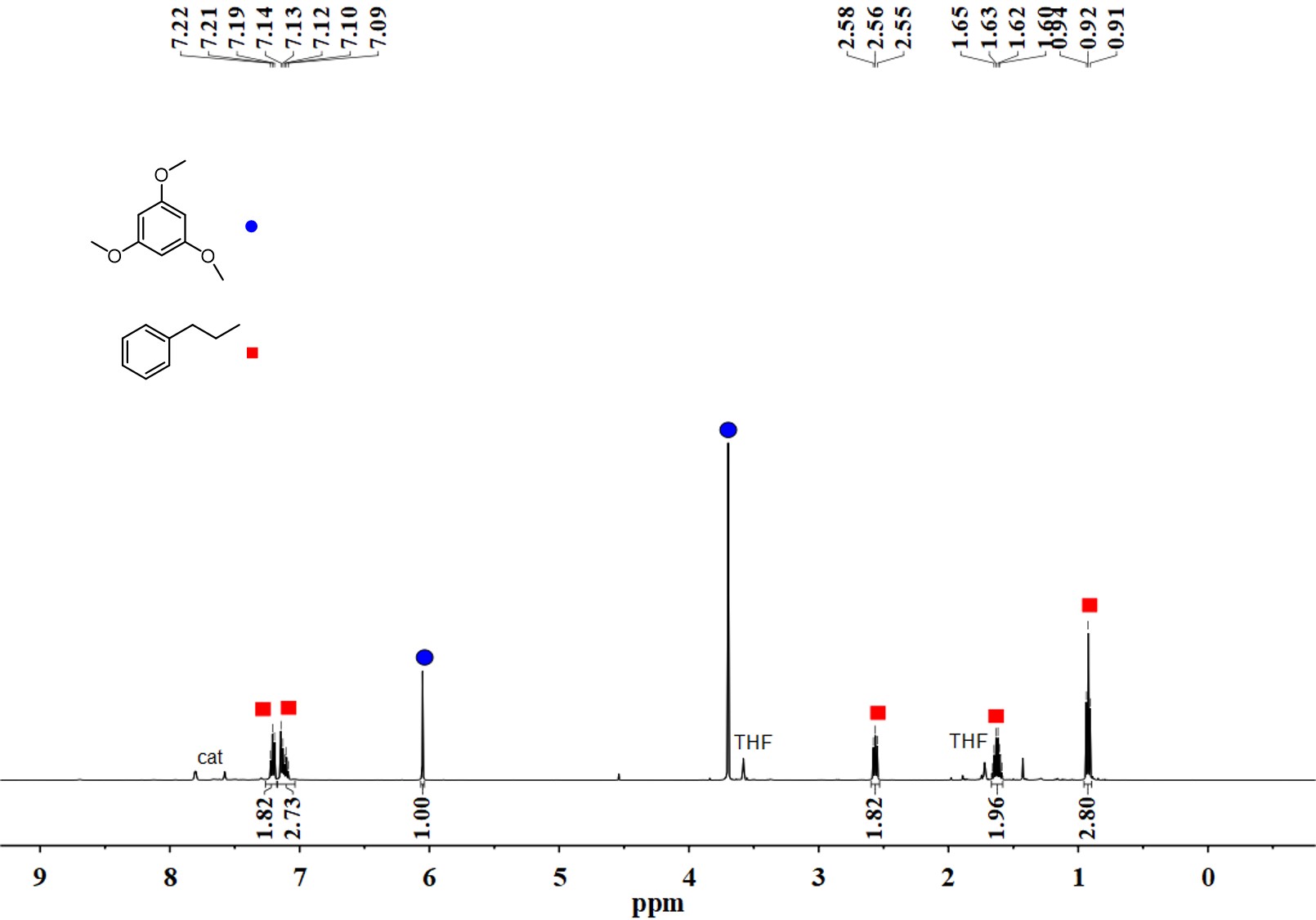


**Supplementary Figure 43.** ^1^H NMR (500 MHz, *d_8_*-THF) spectrum recorded for the catalytic reaction of **5n**.


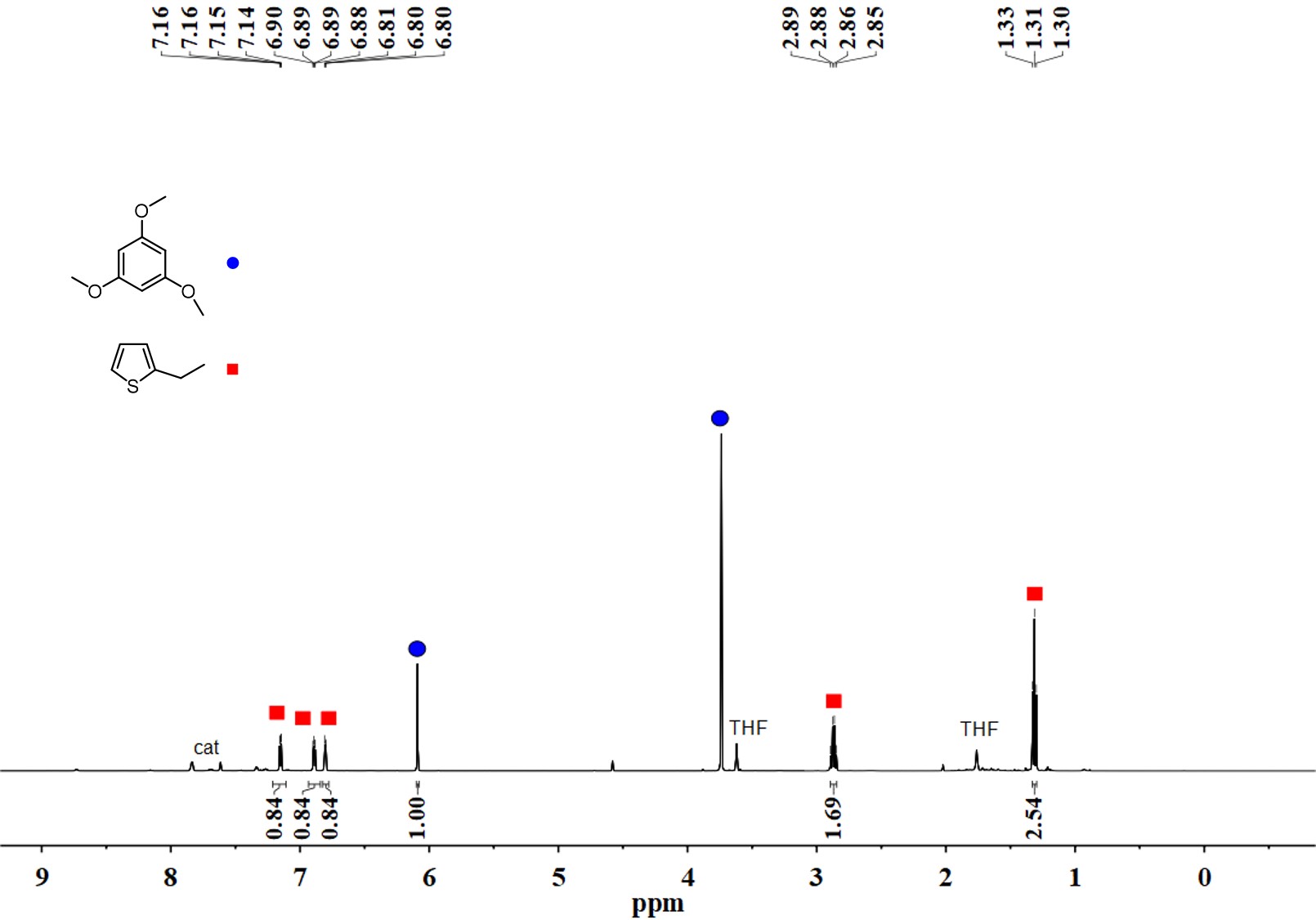


**Supplementary Figure 44.** ^1^H NMR (500 MHz, *d_8_*-THF) spectrum recorded for the catalytic reaction of **5o**.


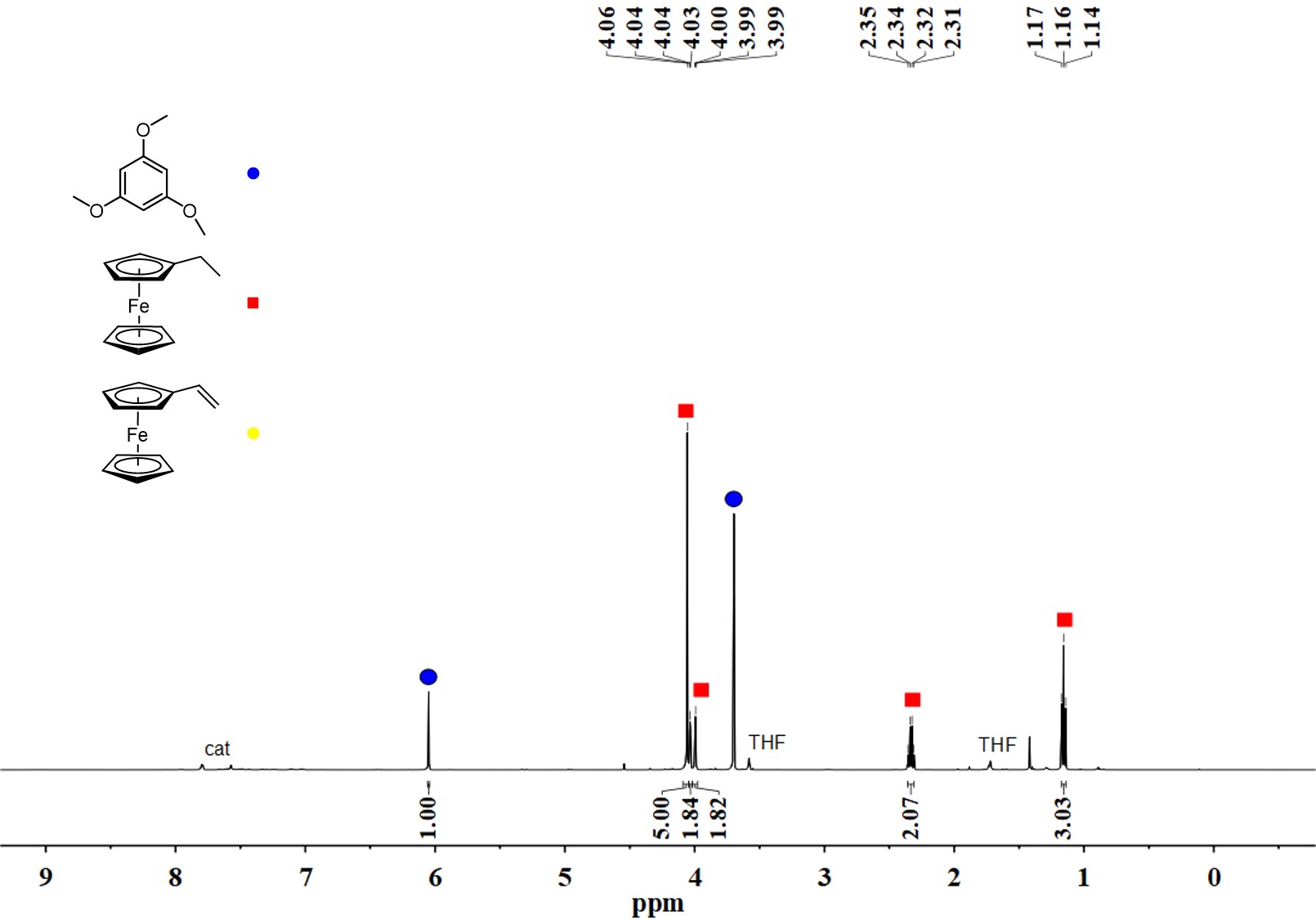


**Supplementary Figure 45.** ^1^H NMR (500 MHz, *d*_8_-THF) spectrum recorded for the catalytic reaction of **5p**.


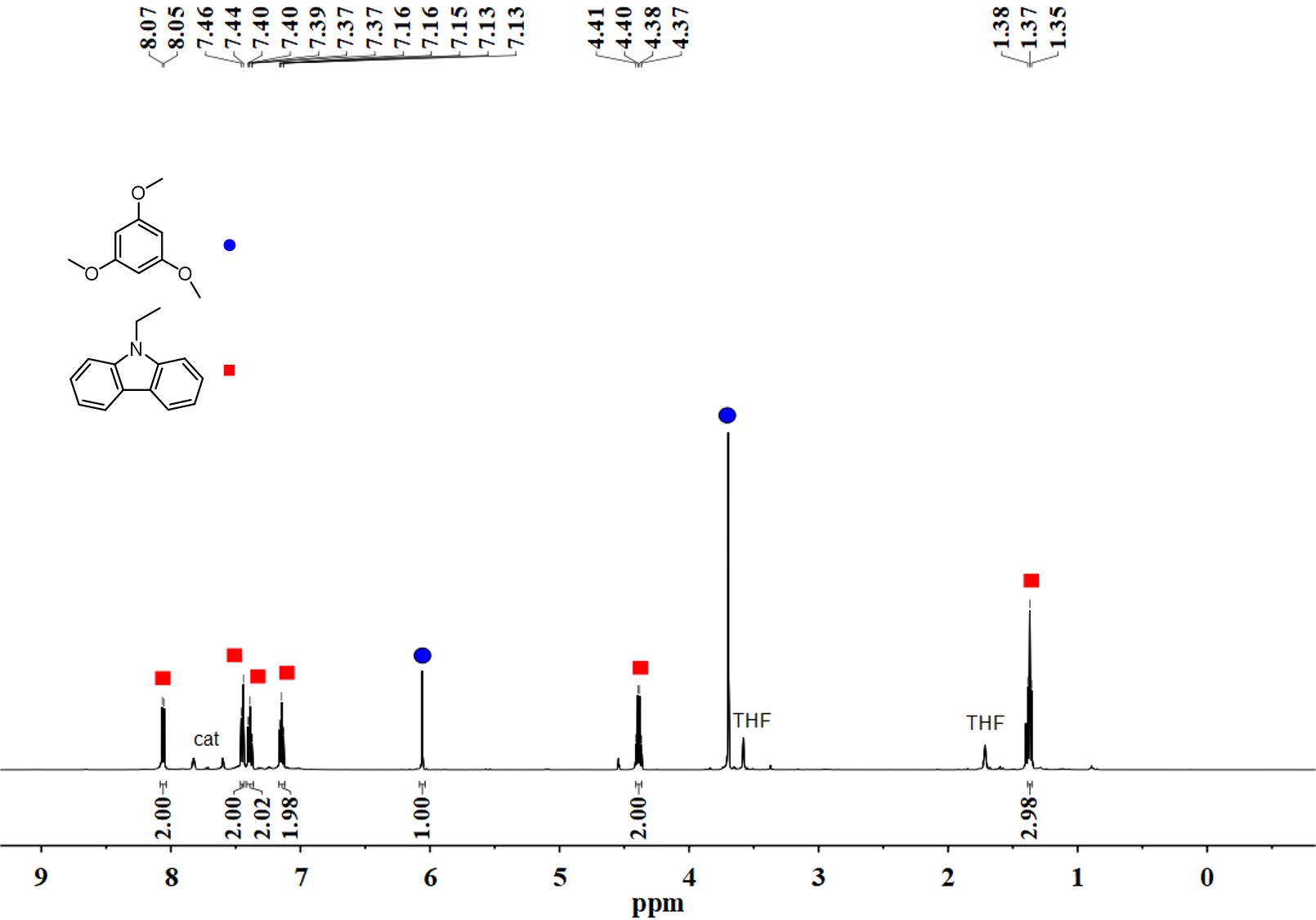


**Supplementary Figure 46.** ^1^H NMR (500 MHz, *d_8_*-THF) spectrum recorded for the catalytic reaction of **5q**.


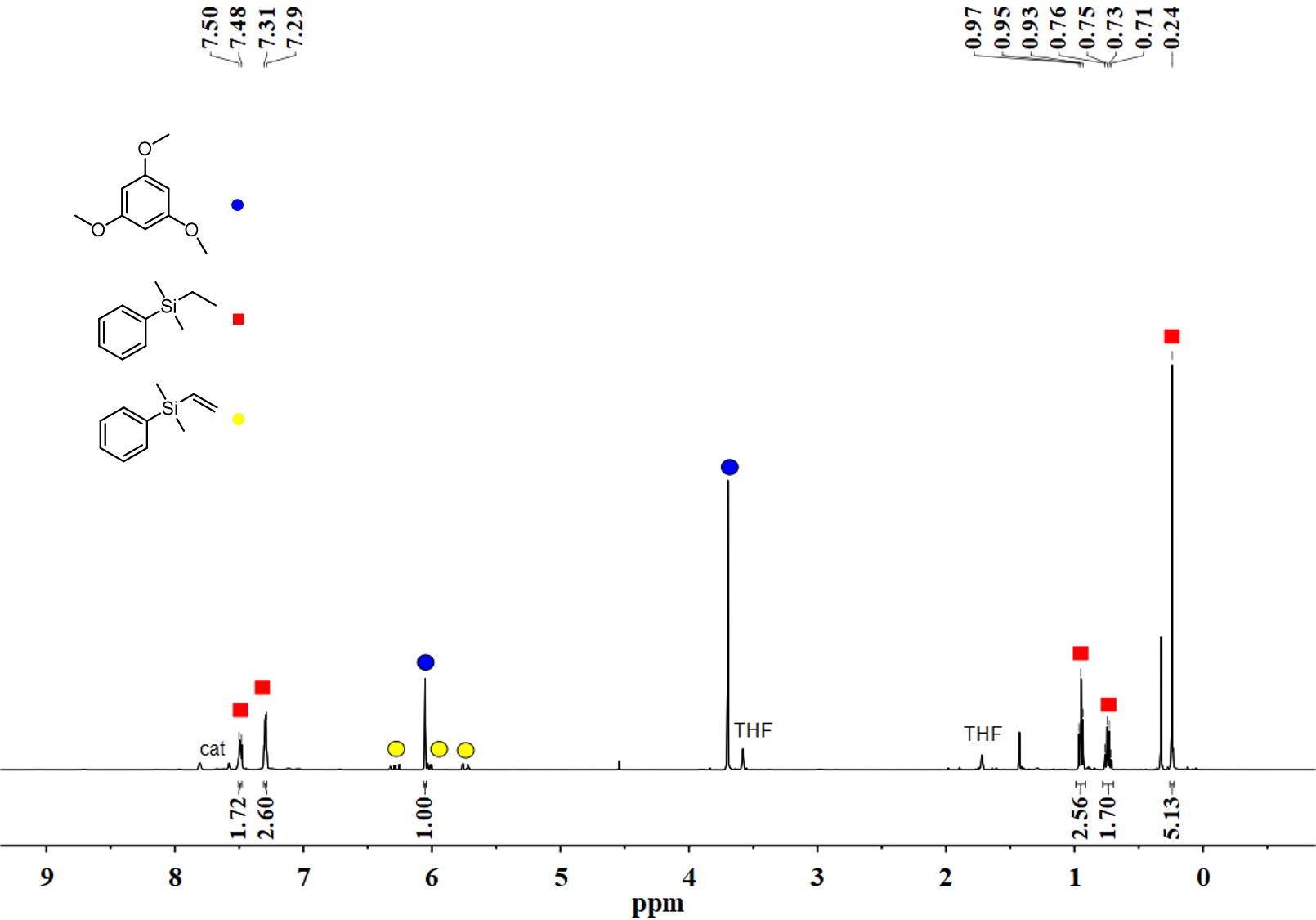


**Supplementary Figure 47.** ^1^H NMR (500 MHz, *d_8_*-THF) spectrum recorded for the catalytic reaction of **5r**.


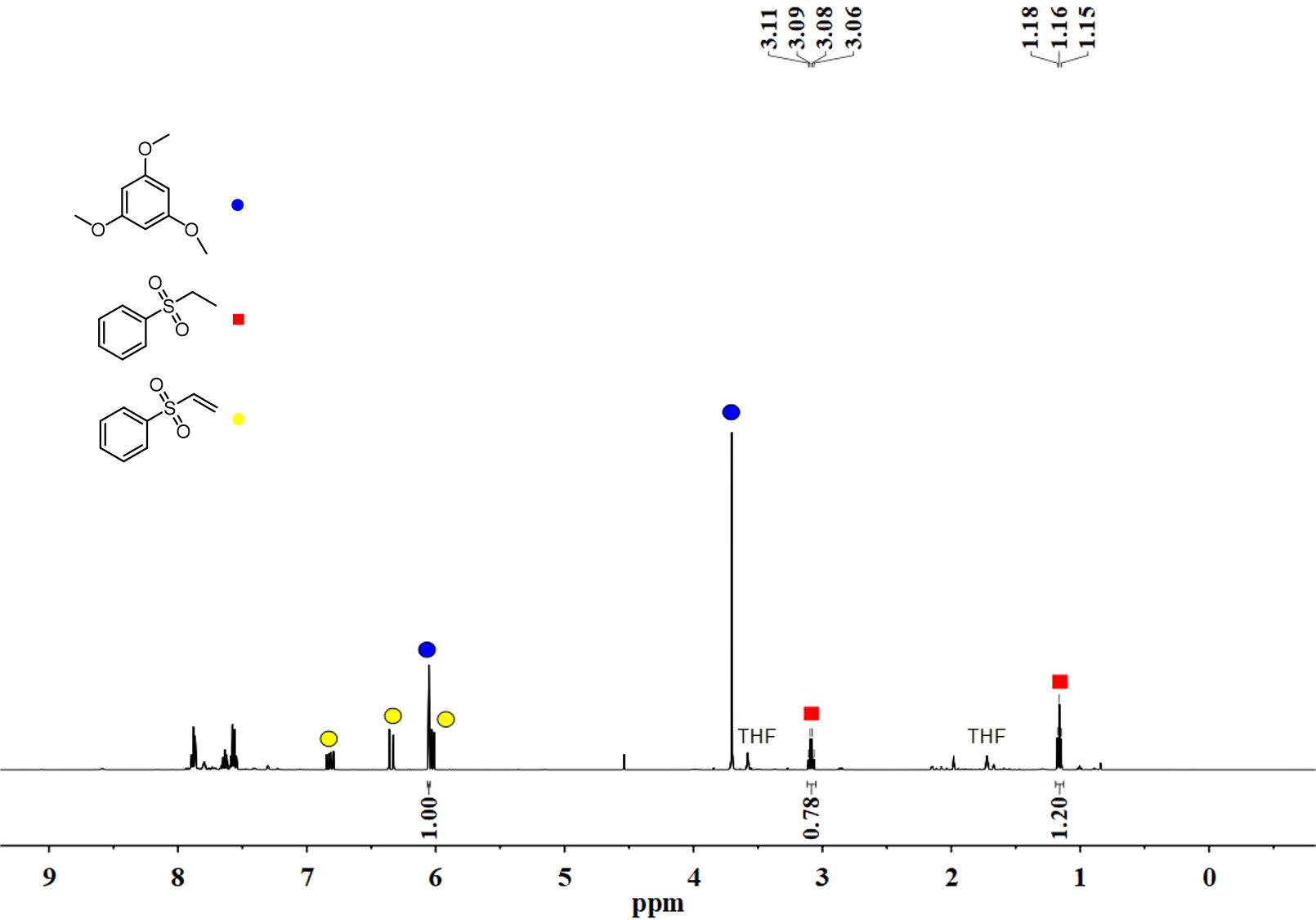


**Supplementary Figure 48**. ^1^H NMR (500 MHz, *d*_8_-THF) spectrum recorded for the catalytic reaction of **5s**.


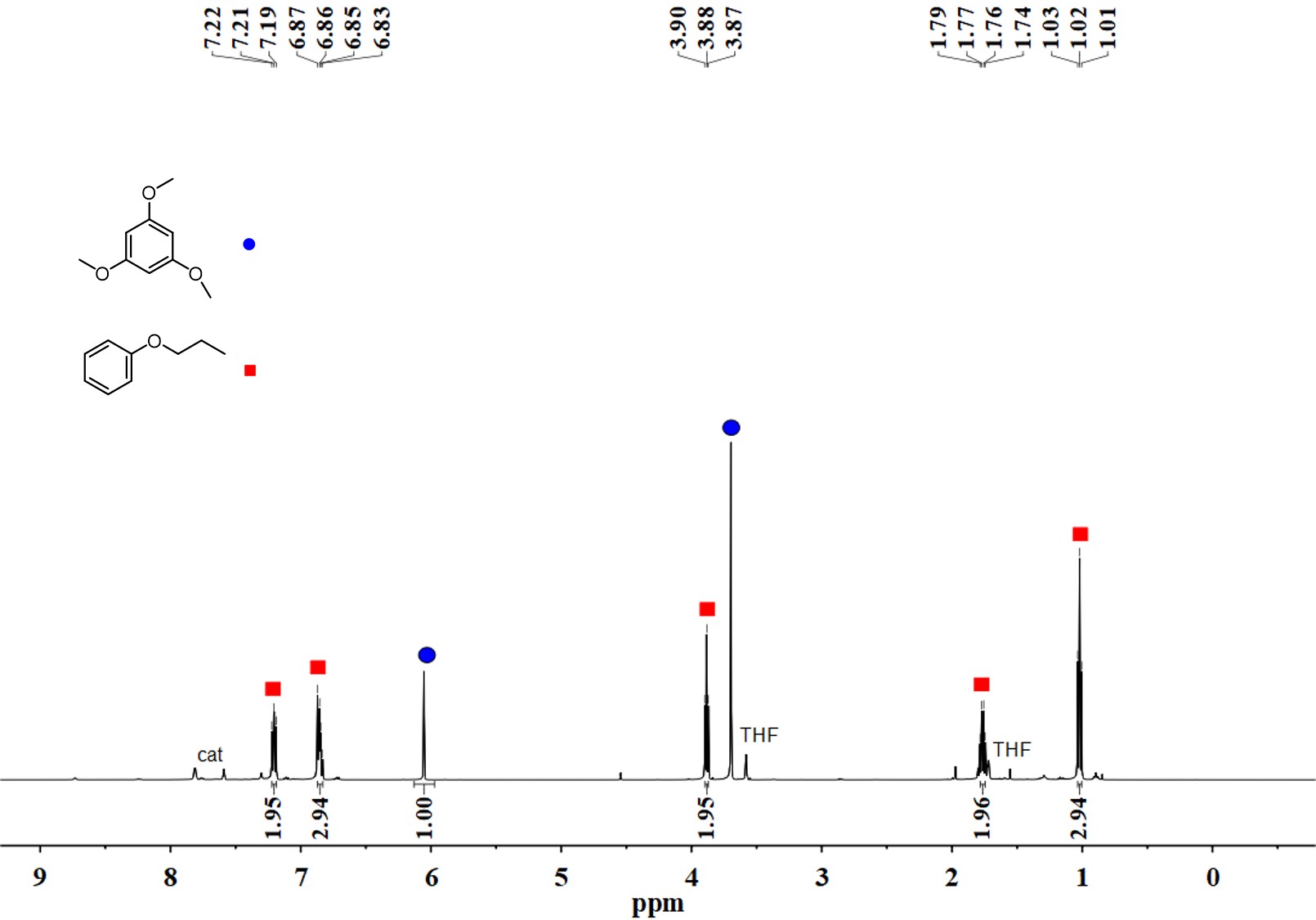


**Supplementary Figure 49.** ^1^H NMR (500 MHz, *d_8_*-THF) spectrum recorded for the catalytic reaction of **5t**.


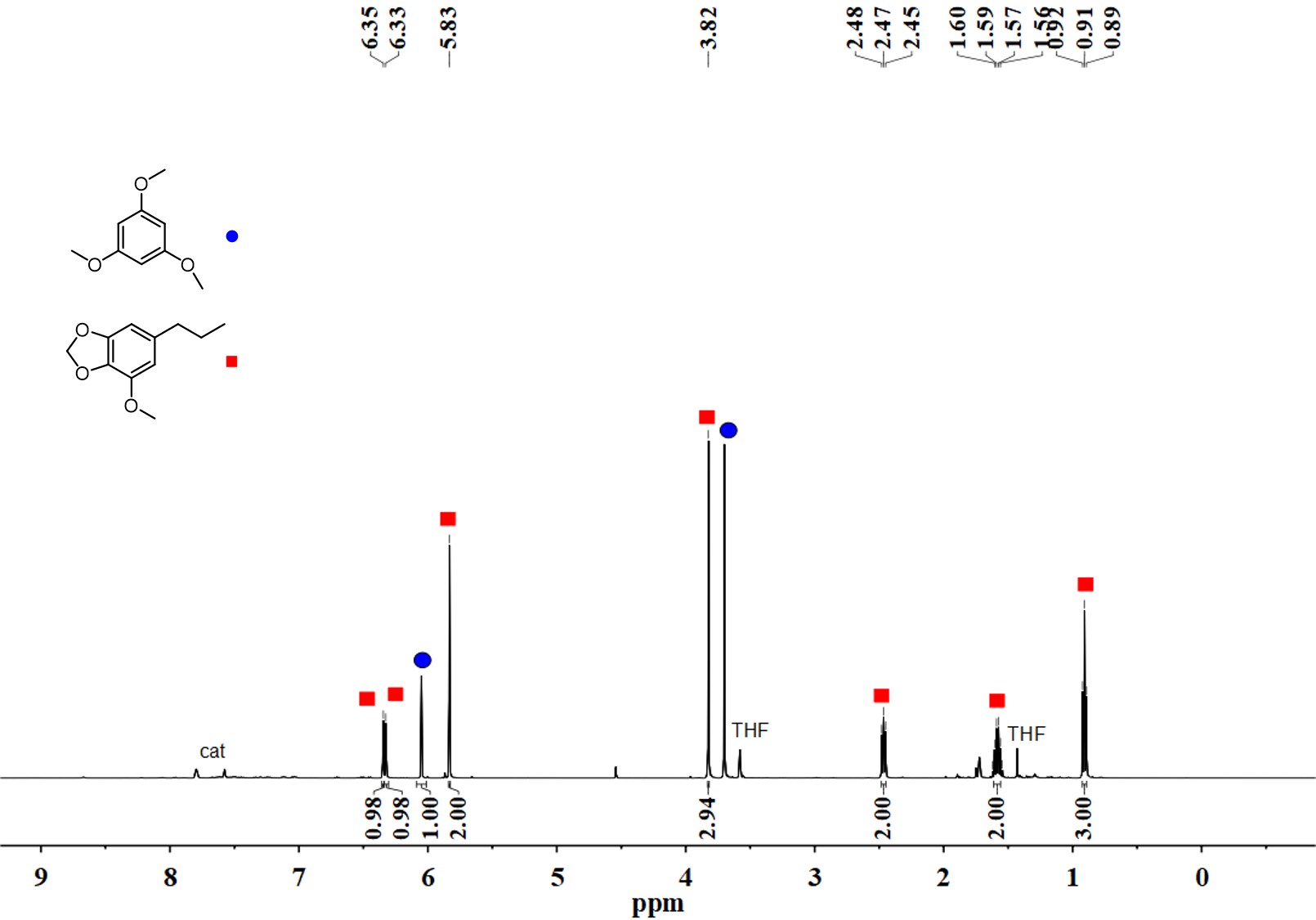


**Supplementary Figure 50.** ^1^H NMR (500 MHz, *d_8_*-THF) spectrum recorded for the catalytic reaction of **5u.**


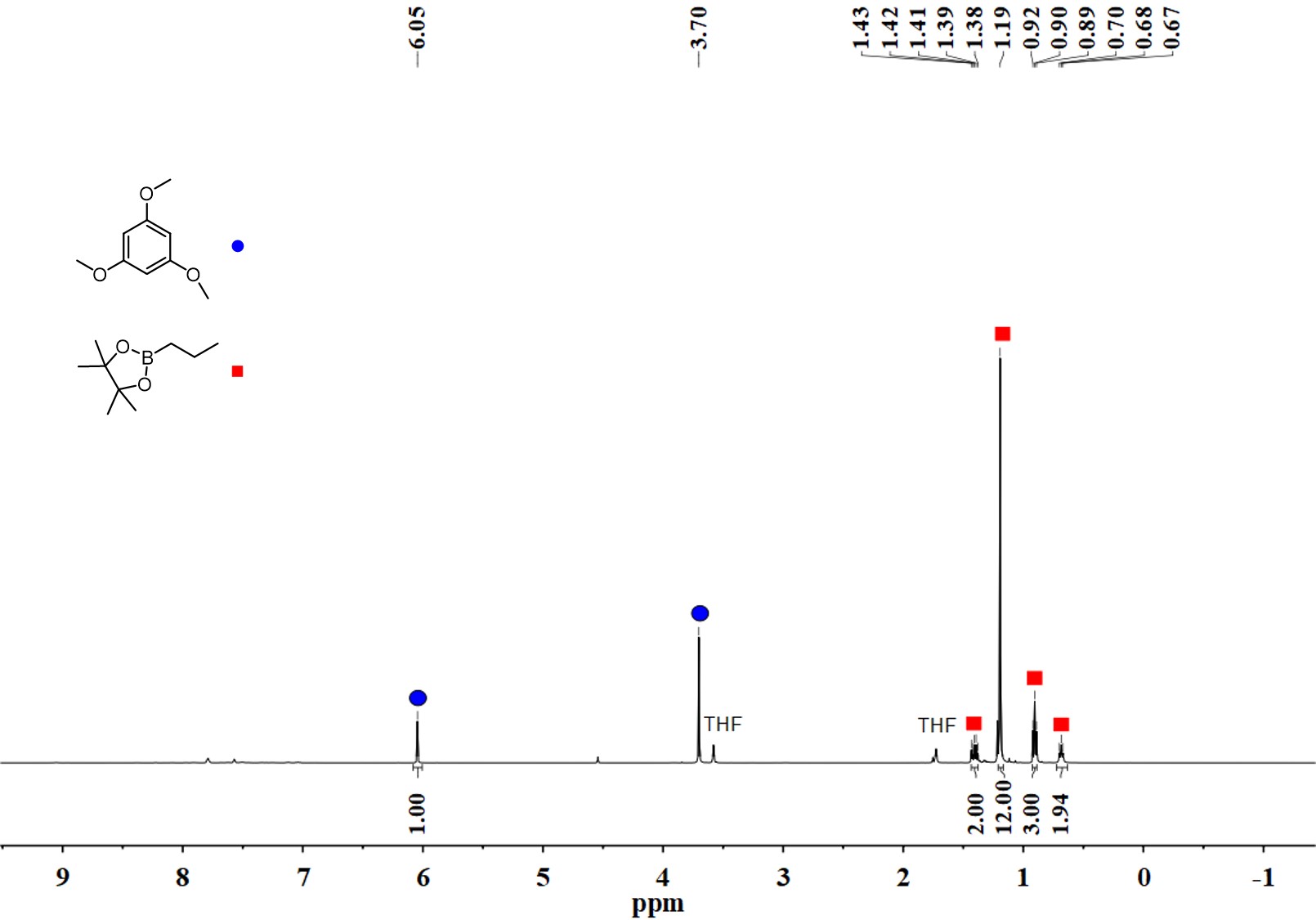


**Supplementary Figure 51.** ^1^H NMR (500 MHz, *d_8_*-THF) spectrum recorded for the catalytic reaction of **5v**.


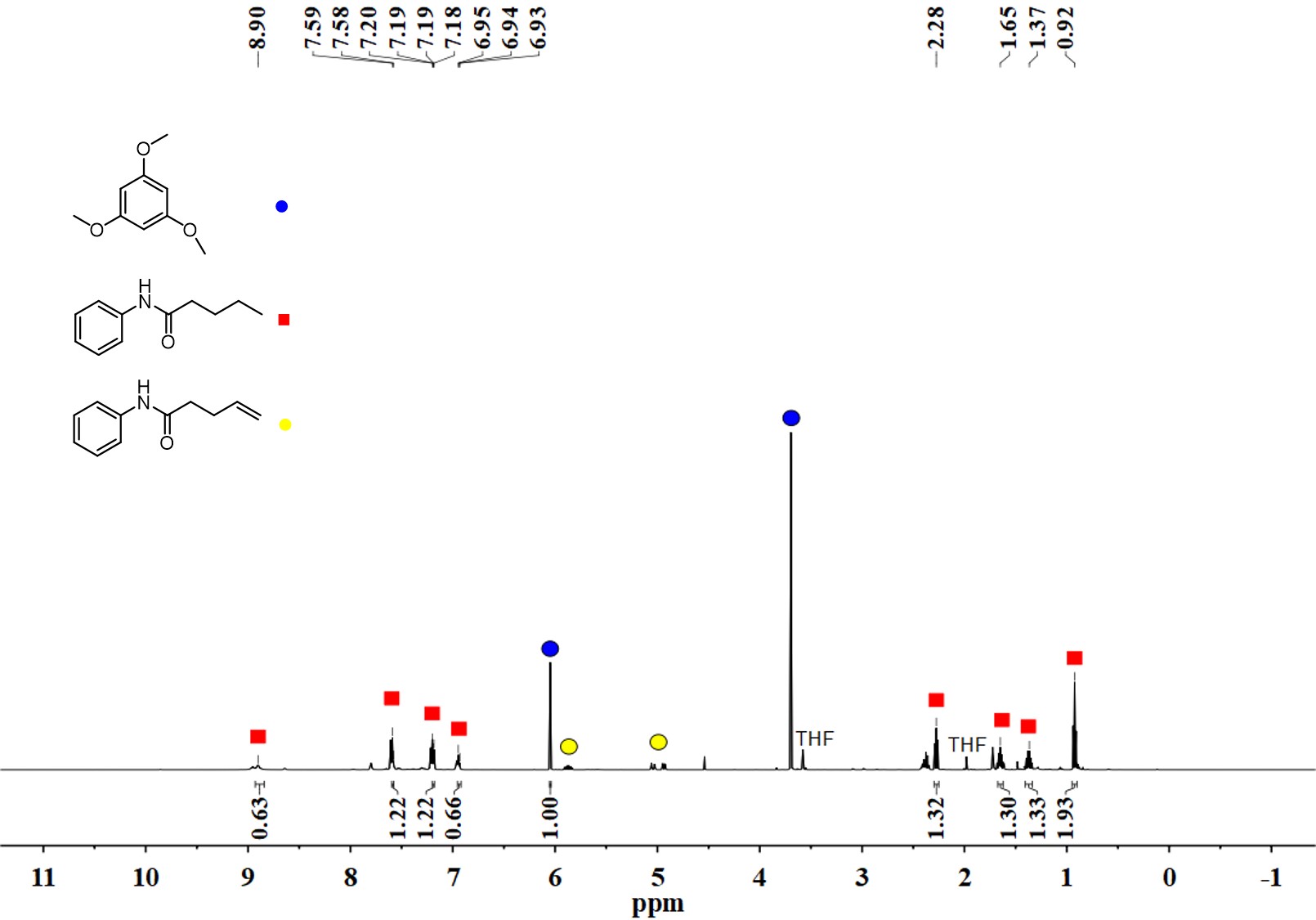


**Supplementary Figure 52.** ^1^H NMR (500 MHz, *d_8_*-THF) spectrum recorded for the catalytic reaction of **5w**.


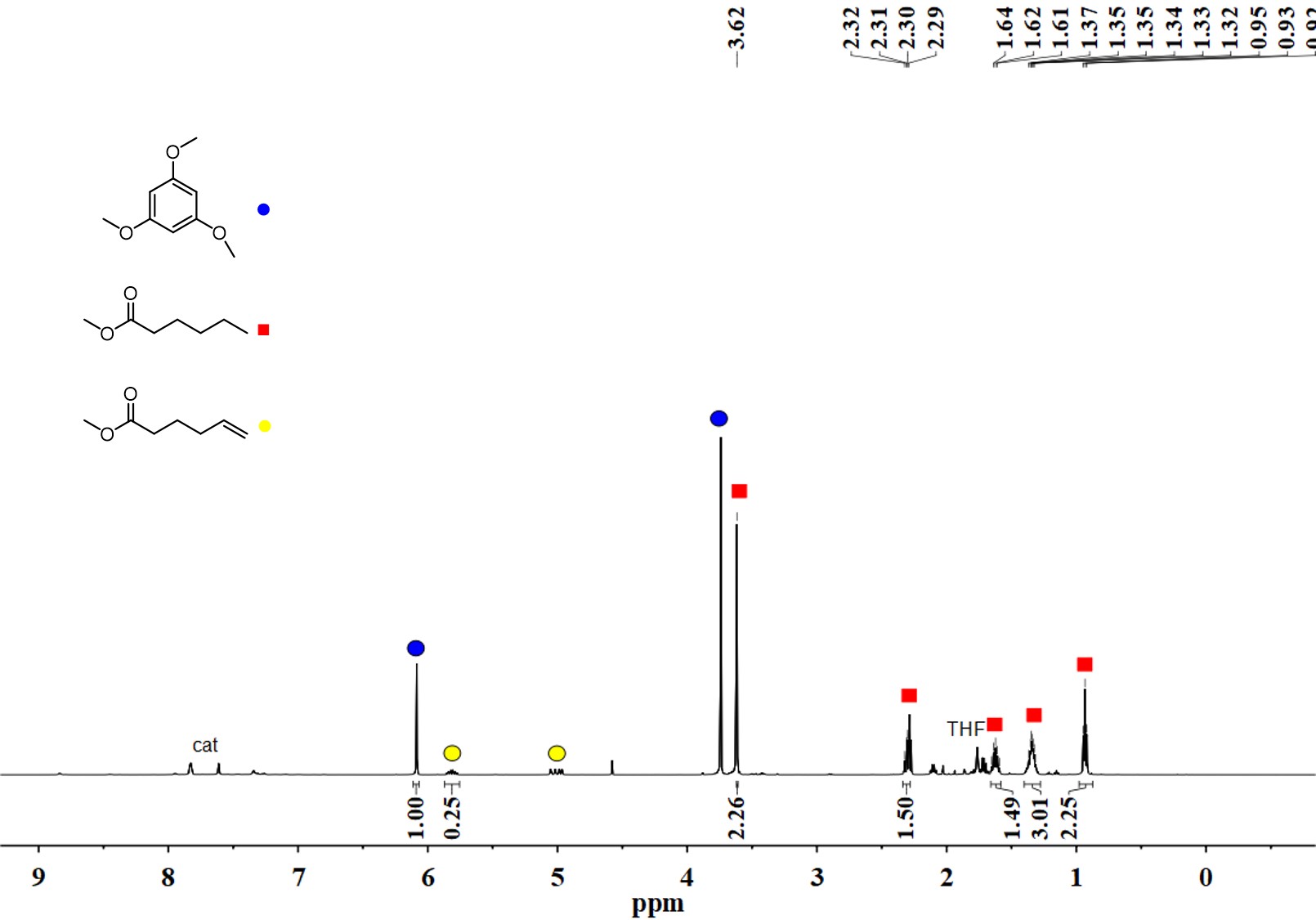


**Supplementary Figure 53.** ^1^H NMR (500 MHz, *d_8_*-THF) spectrum recorded for the catalytic reaction of **5x**.


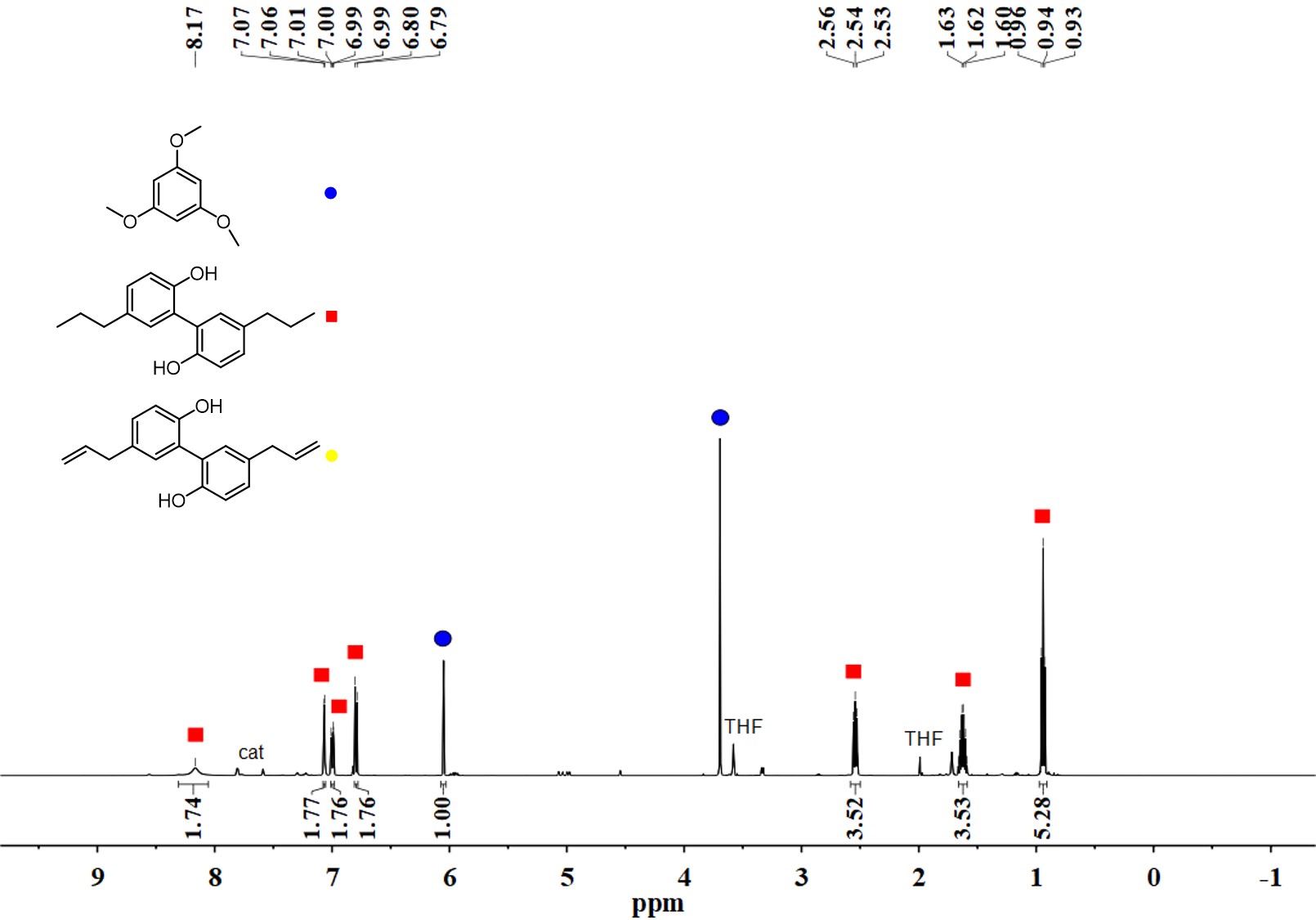


**Supplementary Figure 54.** ^1^H NMR (500 MHz, *d_8_*-THF) spectrum recorded for the catalytic reaction of **5y**.


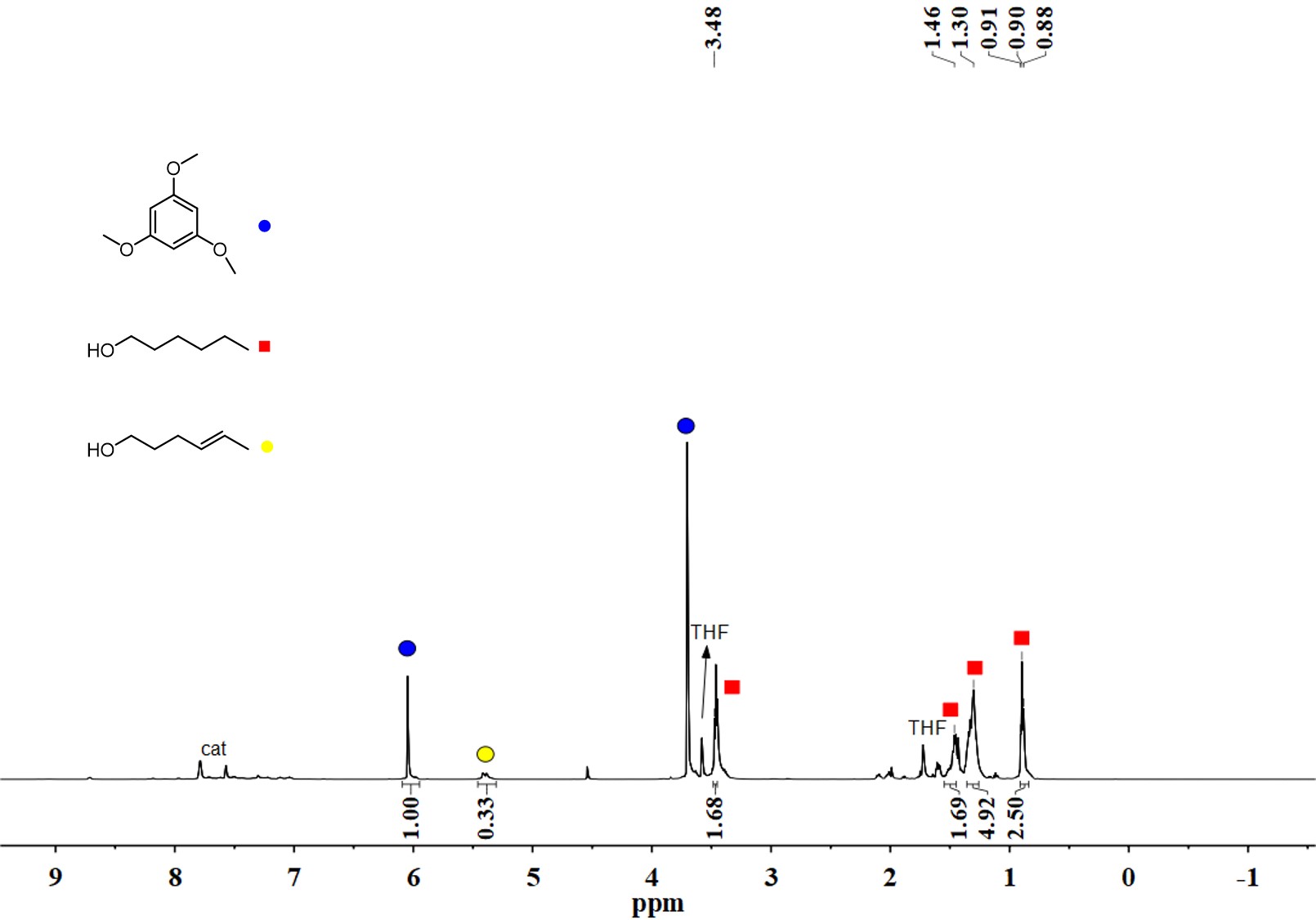


**Supplementary Figure 55.** ^1^H NMR (500 MHz, *d_8_*-THF) spectrum recorded for the catalytic reaction of **5z**.


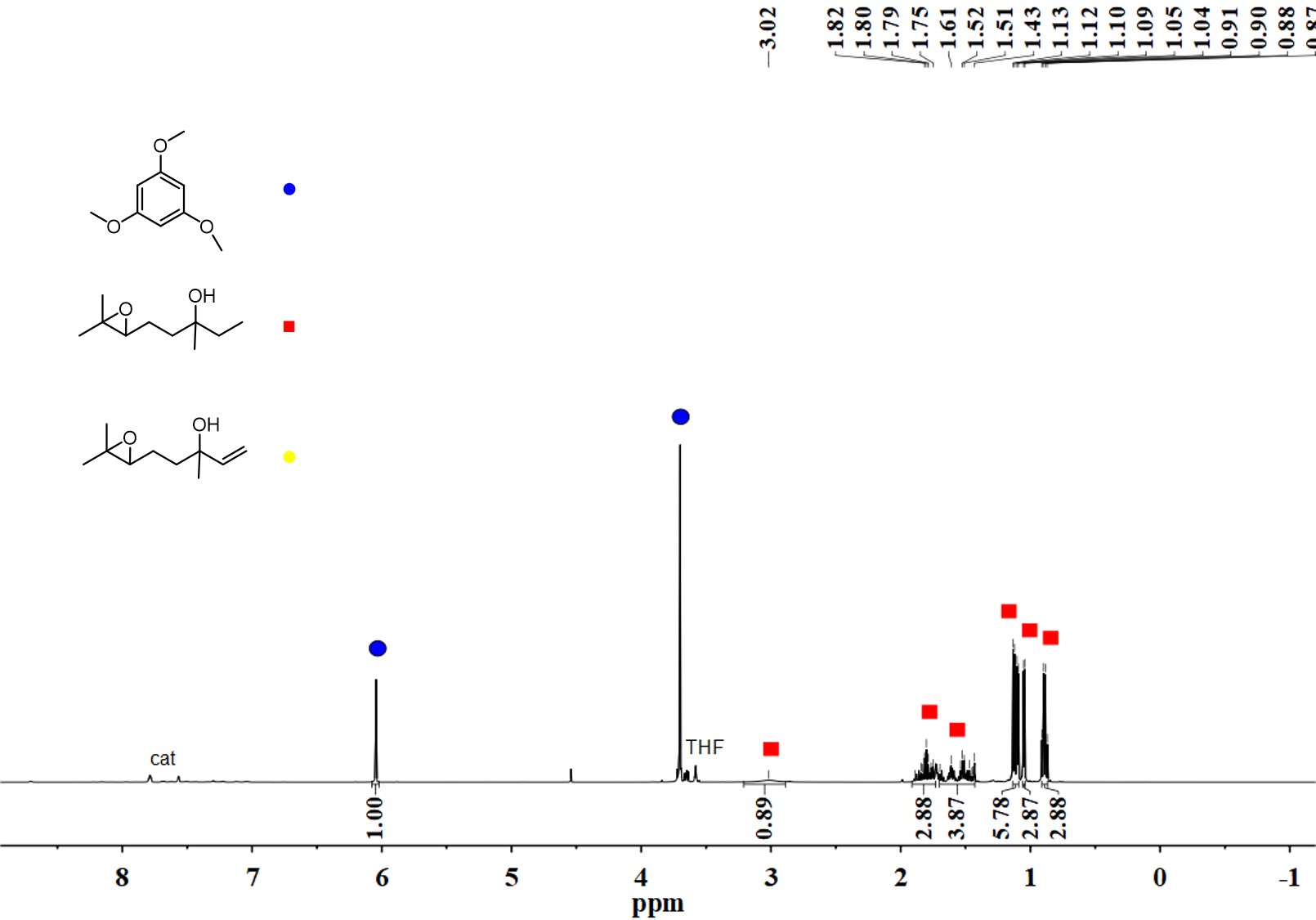


**Supplementary Figure 56.** ^1^H NMR (500 MHz, *d*_8_-THF) spectrum recorded for the catalytic reaction of **5aa**.


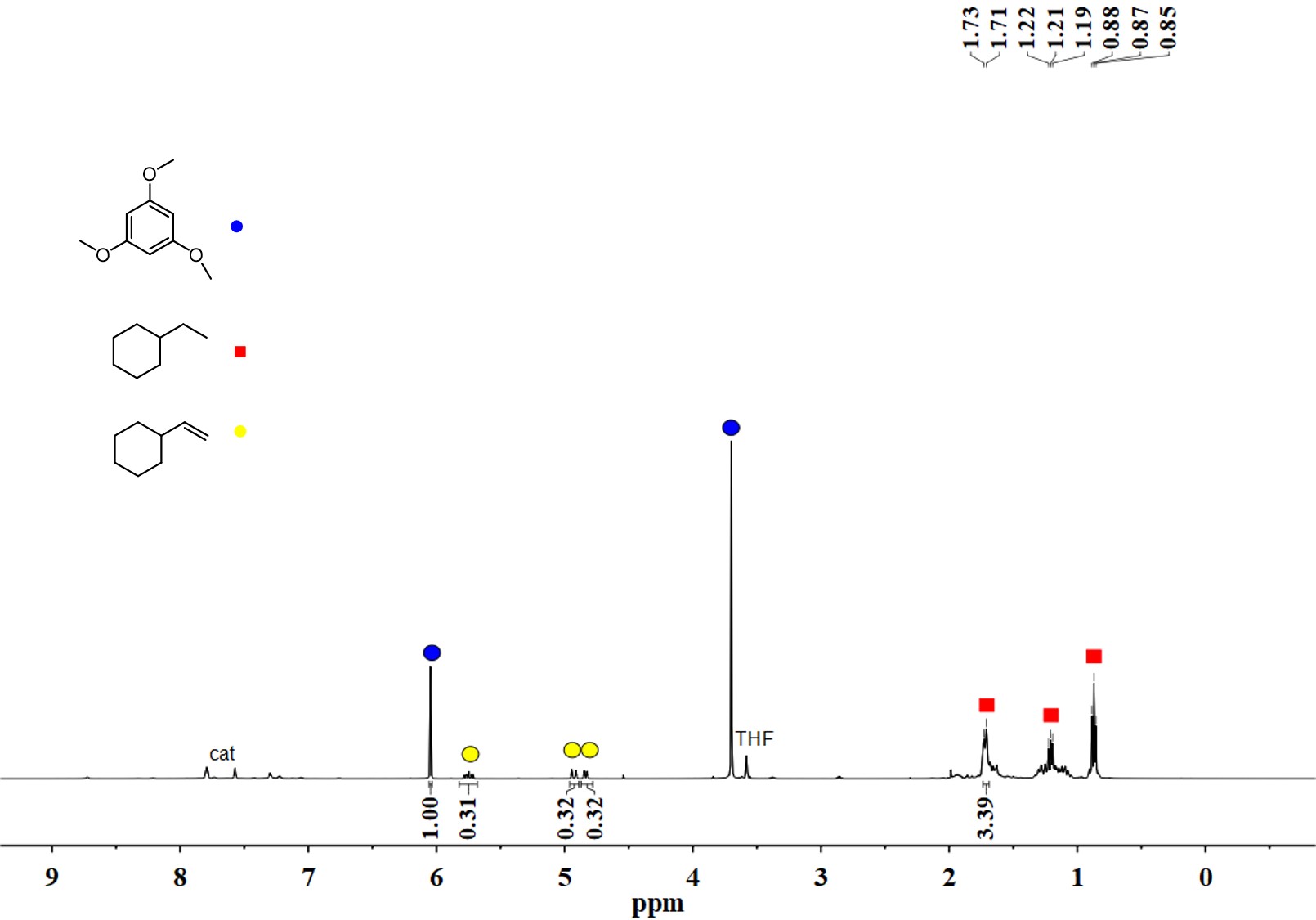


**Supplementary Figure 57.** ^1^H NMR (500 MHz, *d_8_*-THF) spectrum recorded for the catalytic reaction of **5ab**.


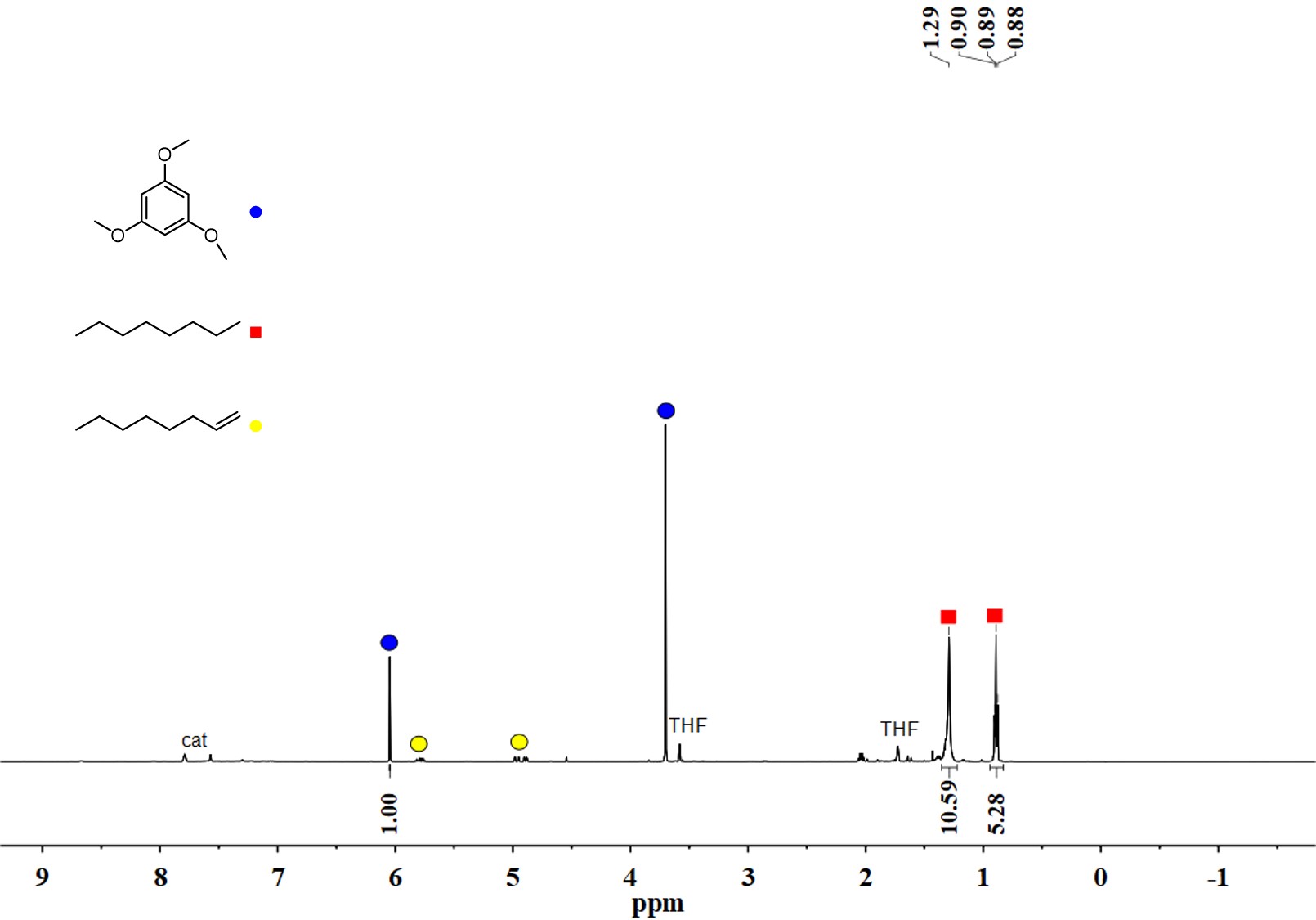


**Supplementary Figure 58.** ^1^H NMR (500 MHz, *d_8_*-THF) spectrum recorded for the catalytic reaction of **5ac**.


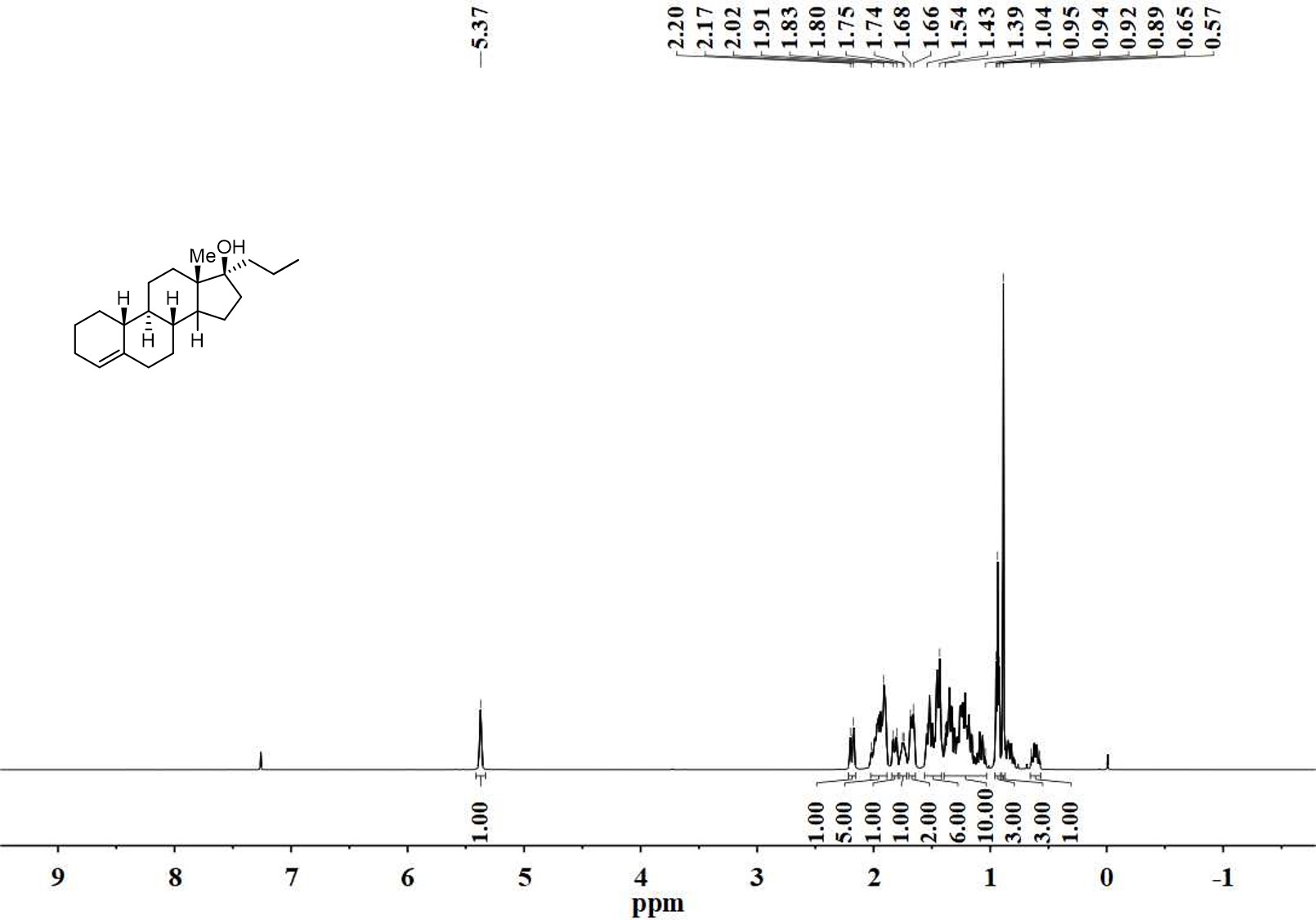


**Supplementary Figure 59.** ^1^H NMR (500 MHz, CDCl_3_) spectrum of **6ad**.


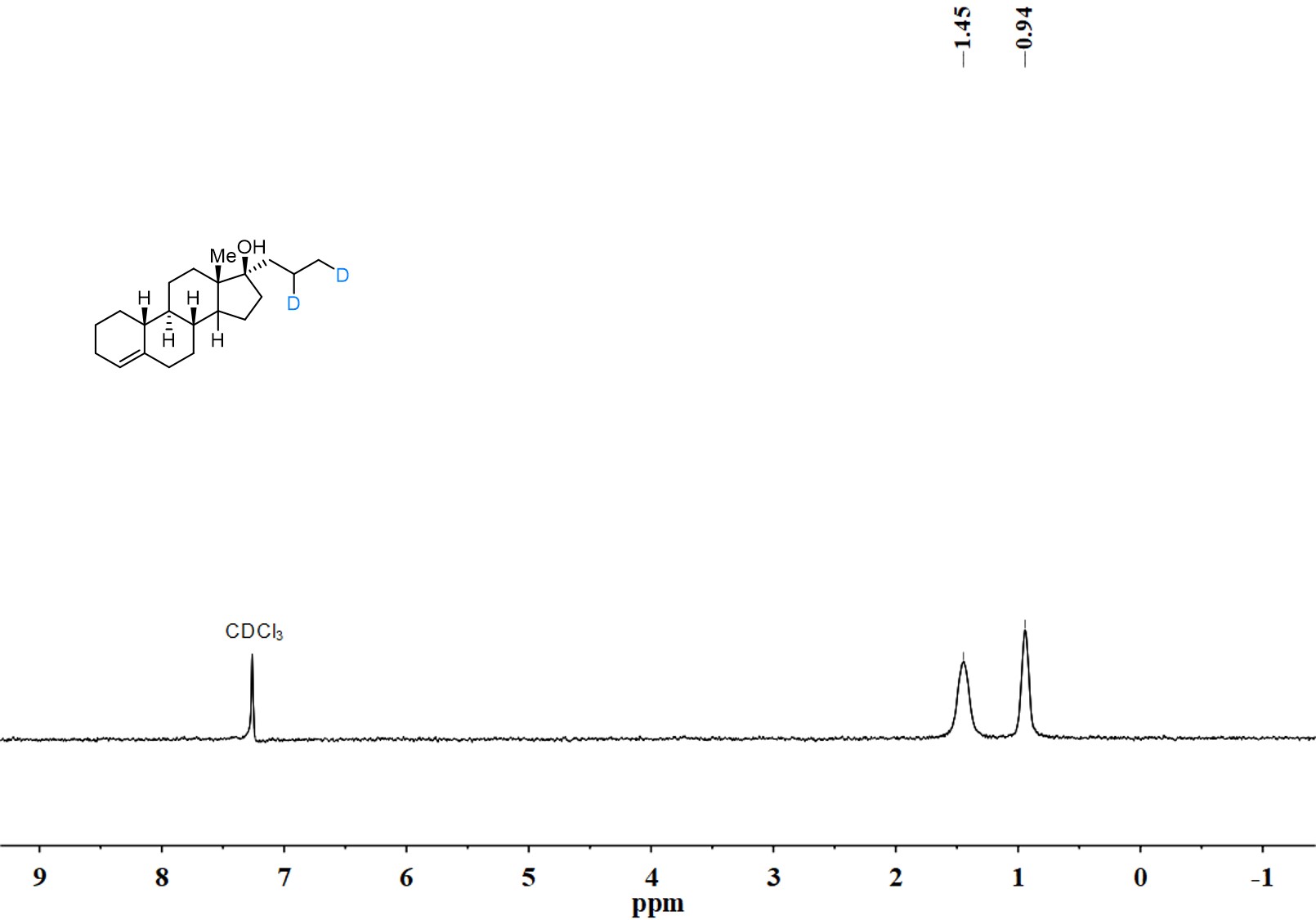


**Supplementary Figure 60.** ^2^H NMR (77 MHz, CHCl_3_) spectrum of *d*_2_-**6ad**.


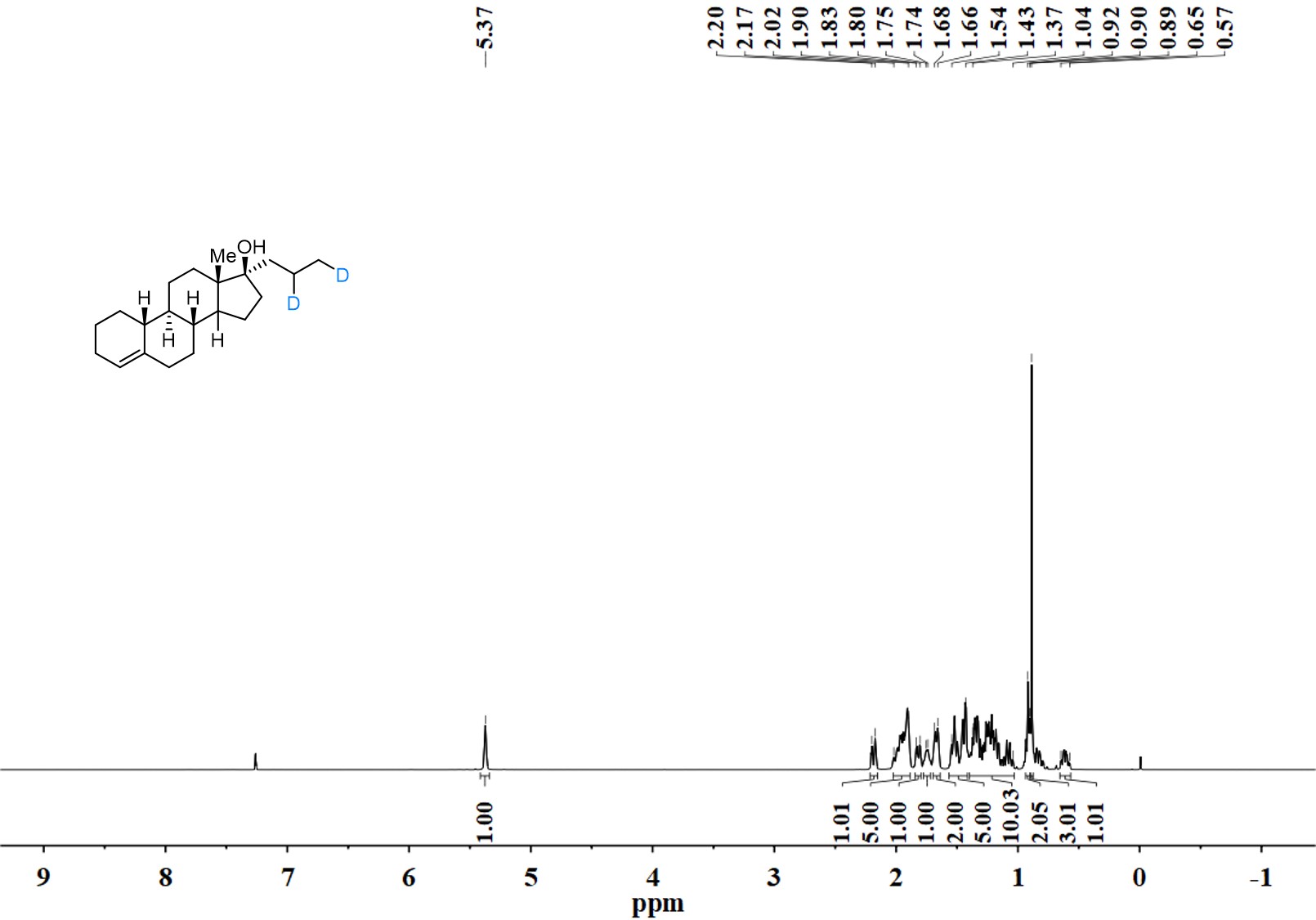


**Supplementary Figure 61.** ^1^H NMR (500 MHz, CDCl_3_) spectrum of *d*_2_-**6ad**.

### Crystal Data and Structure Refinement Parameters

**Supplementary Table 3.** Crystal data and structure refinement parameters for **1**-C_2_H_2_.

|  | **1-**C_2_H_2_ |
| --- | --- |
| CCDC | 2245911 |
| Empirical formula | C_30_H_31_MoPS |
| Formula weight | 550.52 |
| Temperature / K | 173.00(10) |
| Crystal system | triclinic |
| Space group | P-1 |
| *a /* Å | 10.5543(6) |
| *b /* Å | 10.7470(6) |
| *c /* Å | 12.3168(7) |
| α / ° | 70.783(5) |
| β / ° | 89.197(4) |
| γ / ° | 71.742(5) |
| Volume / Å^3^ | 1246.60(13) |
| Z | 2 |
| ρcalc g /cm^−3^ | 1.467 |
| μ / mm^−1^ | 5.803 |
| F(000) | 568.0 |
| Radiation | Cu Kα (λ = 1.54184) |
| 2Θ range for data collection / ° | 8.866 to 152.762 |
| Index ranges | -13 ≤ h ≤ 13,  -13 ≤ k ≤13,  -14 ≤ l ≤ 14 |
| Reflections collected | 12838 |
| Independent reflections | 4865[R_int_ = 0.0444, R_sigma_ = 0.0476] |
| Data/restraints/parameters | 4865/0/303 |
| Goodness-of-fit on F^2^ | 1.085 |
| Final R indexes [I>=2σ (I)] | R_1_ = 0.0321, wR_2_ = 0.0812 |
| Final R indexes [all data] | R_1_ = 0.0347, wR_2_ = 0.0832 |
| Largest diff. peak/hole / e Å^−3^ | 0.55/-0.68 |

**Supplementary Table 4.** Crystal data and structure refinement parameters for **2**.

|  | **2** |
| --- | --- |
| CCDC | 2245912 |
| Empirical formula | C_67_H_49_BF_24_MoNPS |
| Formula weight | 1493.85 |
| Temperature / K | 173.00(10) |
| Crystal system | triclinic |
| Space group | P-1 |
| *a /* Å | 13.1270(4) |
| *b /* Å | 14.2014(4) |
| *c /* Å | 18.2876(6) |
| α / ° | 106.014(3) |
| β / ° | 91.095(3) |
| γ / ° | 90.635(2) |
| Volume / Å^3^ | 3275.84(18) |
| Z | 2 |
| ρcalc g /cm^−3^ | 1.514 |
| μ / mm^−1^ | 0.367 |
| F(000) | 1504.0 |
| Radiation | Mo Kα (λ = 0.71073) |
| 2Θ range for data collection / ° | 4.254 to 52.744 |
| Index ranges | -16 ≤ h ≤ 16,  -17 ≤ k ≤17,  -22 ≤ l ≤ 22 |
| Reflections collected | 32884 |
| Independent reflections | 13239 [R_int_ = 0.0640, R_sigma_ = 0.0789] |
| Data/restraints/parameters | 13239/110/926 |
| Goodness-of-fit on F^2^ | 1.066 |
| Final R indexes [I>=2σ (I)] | R_1_ = 0.0447, wR_2_ = 0.1009 |
| Final R indexes [all data] | R_1_ = 0.0745, wR_2_ = 0.1104 |
| Largest diff. peak/hole / e Å^−3^ | 0.47/-0.76 |

**Supplementary Table 5.** Crystal data and structure refinement parameters for **3**.

|  | **3** |
| --- | --- |
| CCDC | 2245914 |
| Empirical formula | C_70_H_52_BF_24_MoPS |
| Formula weight | 1518.89 |
| Temperature / K | 170.00(10) |
| Crystal system | monoclinic |
| Space group | *C*2*/c* |
| *a /* Å | 38.4277(5) |
| *b /* Å | 13.2586(2) |
| *c /* Å | 27.5437(5) |
| α / ° | 90 |
| β / ° | 107.5853(17) |
| γ / ° | 90 |
| Volume / Å^3^ | 13377.6(4) |
| Z | 8 |
| ρcalc g /cm^−3^ | 1.508 |
| μ / mm^−1^ | 3.096 |
| F(000) | 6128.0 |
| Radiation | Cu Kα (λ = 1.54184) |
| 2Θ range for data collection / ° | 6.732 to 153.282 |
| Index ranges | -47 ≤ h ≤ 35,  -15 ≤ k ≤16,  -33 ≤ l ≤ 34 |
| Reflections collected | 43575 |
| Independent reflections | 12946 [R_int_ = 0.0469, R_sigma_ = 0.0494] |
| Data/restraints/parameters | 12946/0/888 |
| Goodness-of-fit on F^2^ | 1.037 |
| Final R indexes [I>=2σ (I)] | R_1_ = 0.0588, wR_2_ = 0.1523 |
| Final R indexes [all data] | R_1_ = 0.0753, wR_2_ = 0.1640 |
| Largest diff. peak/hole / e Å^−3^ | 1.05/-0.83 |

**Supplementary Table 6.** Crystal data and structure refinement parameters for **4**.

|  | **4** |
| --- | --- |
| CCDC | 2245913 |
| Empirical formula | C_67_H_53_BF_24_MoNPS |
| Formula weight | 1497.88 |
| Temperature / K | 293(2) |
| Crystal system | triclinic |
| Space group | P-1 |
| *a /* Å | 13.5601(4) |
| *b /* Å | 13.6161(4) |
| *c /* Å | 18.4085(4) |
| α / ° | 103.052(2) |
| β / ° | 91.375(2) |
| γ / ° | 91.485(2) |
| Volume / Å^3^ | 3308.39(16) |
| Z | 2 |
| ρcalc g /cm^−3^ | 1.504 |
| μ / mm^−1^ | 3.124 |
| F(000) | 1512.0 |
| Radiation | Cu Kα (λ = 1.54184) |
| 2Θ range for data collection / ° | 6.524 to 153.298 |
| Index ranges | -17 ≤ h ≤ 16,  -14 ≤ k ≤17,  -23 ≤ l ≤ 14 |
| Reflections collected | 35676 |
| Independent reflections | 13055[R_int_ = 0.0568, R_sigma_ = 0.0547] |
| Data/restraints/parameters | 13055/0/904 |
| Goodness-of-fit on F^2^ | 1.049 |
| Final R indexes [I>=2σ (I)] | R_1_ = 0.0856, wR_2_ = 0.2265 |
| Final R indexes [all data] | R_1_ = 0.0962, wR_2_ = 0.2359 |
| Largest diff. peak/hole / e Å^−3^ | 2.47/-1.14 |

### DFT Calculations

The reaction mechanism was computed using Gaussian 16, Revision B.01^21^ with M11L^22^ functionals. The def2svp^23^ basis set was used for all the light elements, whereas SDD basis set was used for molybdenum with effective core potential.^24,25^ All structures were optimized with PCM^26^ solvation (solvent=THF) under the standard conditions. Frequency analysis was then performed on each structure to confirm that it is either an intermediate or a transition state and to obtain thermodynamic data. To compute the chemical shifts of the hydrides in **4**, the structure of **4** was optimized using M11L functionals with PCM solvation (solvent=THF); the tzvp^27^ basis set was used for all the light elements, whereas SDD basis set was used for molybdenum with effective core potential; the nuclear magnetic shielding constants are computed using the GIAO (Gauge Including Atomic Orbital)^28^ method using the same level and referenced against tetramethylsilane computed at the same level.

**Supplementary Figure 62**. Alternative mechanism for catalytic alkene hydrogenation involving the dissociation of S-donor.

*Note 1*: The mechanism involving the dissociation of the thioether S-donor has a similar energetic span to that of the mechanism involving pyridine dissociation. However, the free energy differences between the off-cycle species and unsaturated active species are even greater for the reaction pathway involving S-donor dissociation.

*Note 2*: The computed energetic spans 20.8 (*for protio*) and 20.6 kcal (for *d2*) mol^-1^ for the KIE calculation were rounded from 20.754 and 20.649 kcal mol^-1^, respectively. The actual difference between the two is 0.11 kcal mol^-1^. The KIE is sensitive to the energy difference, i.e., the calculated KIE values using 0.20 and 0.11 kcal mol^-1^ are 0.71 and 0.83, respectively; the latter matches the experimental KIE (0.80) closely.

**Supplementary Figure 63**. Gibbs free energy diagram (in kcal mol^−1^, at 298 K, 1 M concentration, and 1 atmosphere pressure) for the Mo-catalyzed hydrogenation of styrene involving thioether dissociation described in Supplementary Figure 62.

*Note: The atomic coordinates of the optimized structures and the corresponding Gibbs free energies in Hartrees are provided in the source data file. For transition states, the imaginary frequencies are also provided.*

## Supplementary References

1. Hou, S.-F. et al. Cooperative molybdenum-thiolate reactivity for transfer hydrogenation of nitriles. *ACS Catal.* **10**, 380−390 (2020).
2. Tokmic, K., Markus, C. R., Zhu, L.& Fout, A. R. Well-defined cobalt(I) dihydrogen catalyst: experimental evidence for a Co(I)/Co(III) redox process in olefin hydrogenation. *J. Am. Chem. Soc.* **138**, 11907−11913 (2016).
3. Hu, Xiaoping. et al. Ligandless nickel-catalyzed transfer hydrogenation of alkenes and alkynes using water as the hydrogen donor. *Org. Chem. Front.* **6**, 2619–2623 (2019).
4. Carter, T. S., Guiet, L., Frank, D. J., West J. & Thomas, S. P. *Adv. Synth. Catal.* **355**, 880–884 (2013).
5. Takale, B. S., Thakore, R. R., Gao, E. S., Gallou, F. & Lipshutz, B. H. Environmentally responsible, safe, and chemoselective catalytic hydrogenation of olefins: ppm level Pd catalysis in recyclable water at room temperature. *Green Chem.* **22**, 6055−6061 (2020).
6. Osako, T., Torii, K., Tazawa, A. & Uozumi, Y. Continuous-flow hydrogenation of olefins and nitrobenzenes catalyzed by platinum nanoparticles dispersed in an amphiphilic polymer. *RSC Adv.* **5**, 45760−45766 (2015).
7. Holec, C., Hartrampf, U., Neufeld, K. & Pietruszka, J. P450 BM3-catalyzed regio- and stereoselective hydroxylation aiming at the synthesis of phthalides and isocoumarins. *ChemBioChem.* **18**, 676–684 (2017).
8. Krüger, T., Vorndran, K. & Linker, T. Regioselective arene functionalization: simple substitution of carboxylate by alkyl groups. *Chem. Eur. J.* **15**, 12082–12091 (2009).
9. Volkov, A.et al. Mild deoxygenation of aromatic ketones and aldehydes over Pd/C using polymethylhydrosiloxane as the reducing agent. *Angew. Chem. Int. Ed.* **54**, 5122–5126 (2015).
10. Eisch, J. J. & Dutta, S. Carbon−carbon bond formation in the surprising rearrangement of diorganylzirconium dialkoxides:  linear dimerization of terminal olefins. *Organometallics* **24**, 3355−3358 (2005).
11. Xu, X., Kehr, G., Daniliuc, C. G. & Erker, G. Stoichiometric reactions and catalytic hydrogenation with a reactive intramolecular Zr+/amine frustrated Lewis pair. *J. Am. Chem. Soc.* **137**, 4550−4557 (2015).
12. Manbeck, G. F. et al. Organosoluble copper clusters as precatalysts for carbon-heteroelement bond-forming reactions: microwave and conventional heating. *J. Org. Chem.* **70**, 244−250 (2005).
13. Linne, Y., Schönwald, A., Weißbach, S. & Kalesse, M. Desymmetrization of C2-symmetric bis(boronic esters) by zweifel olefinations. *Chem. Eur. J.* **26**, 7998–8002 (2020).
14. Zhou, W., Fan, W., Jiang, Q., Liang, Y.-F. & Jiao, N. Copper-catalyzed aerobic oxidative C–C bond cleavage of unstrained ketones with air and amines. *Org. Lett.* **17**, 2542–2545 (2015).
15. Yamamoto, N., Obora, Y. & Ishii, Y. Iridium-catalyzed oxidative methyl esterification of primary alcohols and diols with methanol. *J. Org. Chem.* **76**, 2937−2941 (2011).
16. Zhang, C. & Rao, Y. Weak coordination promoted regioselective oxidative coupling reaction for 2,2′-difunctional biaryl synthesis in hexafluoro-2-propanol. *Org. Lett.* **17**, 4456–4459 (2015).
17. Brett Runge, M., Mwangi, M. T. & Bowden, N. B. New selectivities from old catalysts. Occlusion of Grubbs’ catalysts in PDMS to change their reactions. *J. Organomet. Chem.* **691**, 5278−5288 (2006).
18. Yamazaki, S. An effective procedure for the synthesis of acid-sensitive epoxides: Use of 1-methylimidazole as the additive on methyltrioxorhenium-catalyzed epoxidation of alkenes with hydrogen peroxide. *Org. Biomol. Chem.* **8**, 2377−2385 (2010).
19. Ghosh, S. & Jagirdar, B. R. Synthesis of mesoporous iridium nanosponge: a highly active, thermally stable and efficient olefin hydrogenation catalyst. *Dalton Trans.* **46**, 11431–11439 (2017).
20. Gnaim, S. et al. Cobalt-electrocatalytic HAT for functionalization of unsaturated C–C bonds. *Nature* **605**, 687–695 (2022).
21. Frisch, M. J. et al. Gaussian 16 Revision B.01 (Gaussian, Inc. Wallingford CT, 2016).
22. Peverati, R. & Truhlar, D. G. M11-L: A local density functional that provides improved accuracy for electronic structure calculations in chemistry and physics. *J. Phys. Chem. Lett.* **3**, 117–124 (2012).
23. Weigend, F. & Ahlrichs, R., Balanced basis sets of split valence, triple zeta valence and quadruple zeta valence quality for H to Rn: design and assessment of accuracy. *Phys. Chem. Chem. Phys.* **7**, 3297–3305 (2005).
24. Dunning, T. H. & Hay, P. J. in Modern Theoretical Chemistry III, Vol. 3, (Eds: Schaefer, H. F.), Plenum, New York, 1977, pp. 1–28.
25. Andrae, D., Häußermann, U., Dolg, M., Stoll, H. & Preuß, H. Energy-adjustedab initio pseudopotentials for the second and third row transition elements. *Theor. Chem. Acc.* **7**, 123–141 (1990).
26. Tomasi, J., Mennucci, B. & Cammi, R. Quantum mechanical continuum solvation models. *Chem. Rev.* **105**, 2999–3094 (2005).
27. Schaefer, A., Huber, C., & Ahlrichs, R. Fully optimized contracted Gaussian-basis sets of triple zeta valence quality for atoms Li to Kr. *J. Chem. Phys.* **100**, 5829–5835 (1994).
28. Schreckenbach, G., Ziegler, T. Calculation of NMR shielding tensors using gauge-including atomic orbitals and modern density functional theory. *J. Phys. Chem.* **99**, 606–611 (1995).
